# Green and Facile Assembly of Diverse Fused *N*-Heterocycles Using Gold-Catalyzed Cascade Reactions in Water

**DOI:** 10.3390/molecules24050988

**Published:** 2019-03-11

**Authors:** Xiuwen Jia, Pinyi Li, Xiaoyan Liu, Jiafu Lin, Yiwen Chu, Jinhai Yu, Jiang Wang, Hong Liu, Fei Zhao

**Affiliations:** 1 Antibiotics Research and Re-evaluation Key Laboratory of Sichuan Province, Sichuan Industrial Institute of Antibiotics, Chengdu University, Chengdu 610052, China; jiaxiuwen2018@126.com (X.J.); pinyiLi19950206@126.com (P.L.); 19940826097@163.com (X.L.); siiakyb@139.com (Y.C.); 2 School of Biological Science and Technology, University of Jinan, Jinan 250022, China; bio_yujh@ujn.edu.cn; 3 State Key Laboratory of Drug Research and CAS Key Laboratory of Receptor Research, Shanghai Institute of Materia Medical, Chinese Academy of Sciences, Shanghai 201203, China; jwang@simm.ac.cn; 4 University of Chinese Academy of Sciences, Beijing 100049, China

**Keywords:** amine nucleophiles, alkynoic acids, cascade reaction, gold catalysis, fused *N*-heterocycles

## Abstract

The present study describes an AuPPh_3_Cl/AgSbF_6_-catalyzed cascade reaction between amine nucleophiles and alkynoic acids in water. This process proceeds in high step economy with water as the sole coproduct, and leads to the generation of two rings, together with the formation of three new bonds in a single operation. This green cascade process exhibits valuable features such as low catalyst loading, good to excellent yields, high efficiency in bond formation, excellent selectivity, great tolerance of functional groups, and extraordinarily broad substrate scope. In addition, this is the first example of the generation of an indole/thiophene/pyrrole/pyridine/naphthalene/benzene-fused N-heterocycle library through gold catalysis in water from readily available materials. Notably, the discovery of antibacterial molecules from this library demonstrates its high quality and potential for the identification of active pharmaceutical ingredients.

## 1. Introduction

Rapid advances in genomics and proteomics have resulted in the identification of an increasing number of novel therapeutic targets [1,2,3,4,5], and the existing compound libraries can no longer well meet the needs of drug screening. Therefore, it is highly demanding to develop robust synthetic methods to construct new compound libraries for drug discovery aimed at these targets [6,7,8]. Considering the structurally diverse targets in a wide “biological space”, high-throughput screening (HTS) of skeletally diverse compounds, which occupy a broader “chemical space”, can apparently enhance the hit rates [9,10]. In addition to skeletal diversity, drug-like properties of the compounds are equally important for the generation of high-quality compound libraries [11,12,13,14], which can increase the possibility of identifying drug-like hit compounds. As a result, privileged structures have received wide attention in drug discovery because they are widely found in natural and pharmaceutical products [15,16,17,18,19]. Although privileged substructure-based diversity-oriented synthesis (pDOS) provides a useful access to assemble compound libraries with high-quality [20,21,22,23,24,25,26], it is still challenging to develop efficient and practical approaches to generate a variety of molecular frameworks embedded with privileged structures, especially in a green and sustainable manner. With our interests to develop green and efficient protocols to synthesize valuable N-heterocycles [27,28,29,30,31], we herein construct a library of privileged substructure-based N-heterocycles with diverse scaffolds using gold-catalyzed cascade reactions in water. To the best of our knowledge, this is the first example of the generation of pDOS compound library encompassing skeletal diversity, molecular complexity, and drug-like properties through gold catalysis in water.

**Scheme 1:**
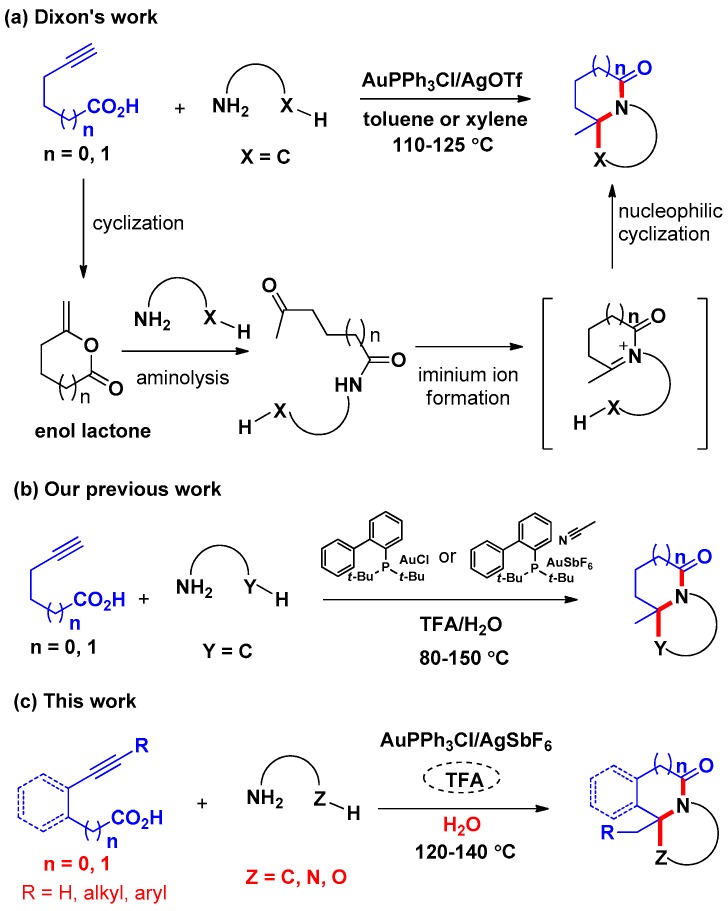
Gold-catalyzed tandem reactions between alkynoic acids and amine nucleophiles. (**a**). Dixon’s work. (**b**). Our previous work. (**c**). This work.

Alkynoic acids are extensively used to react with amine nucleophiles to furnish heterocyclic compounds because of the efficient cascade reaction developed by Dixon’s group [32], in which, an activated cyclic enol lactone species, which derives from alkynoic acids, is involved as the key intermediate (Scheme 1a). Dixon and co-workers disclosed linear aliphatic terminal alkynoic acids reacted smoothly with amine nucleophiles bearing a nucleophilic carbon atom in toluene or xylene to produce pyrrole- or indole-based heterocyclic frameworks catalyzed by AuPPh_3_Cl/AgOTf. It should be noted that, based on Dixon’s pioneering work, the reactions of alkynoic acids with amine nucleophiles in aprotic solvents such as toluene, xylene, 1,2-dichloroethane, dichloromethane, etc., have been well studied by Patil’s group as well as our group [33,34,35,36,37,38,39,40,41,42,43]. However, the reactivity of alkynoic acids and amine nucleophiles in the environmentally friendly, abundant, and cheap solvent—water—was seldom explored [44,45], mainly because the ring opening reaction of the activated enol lactone intermediate in water at elevated temperature may lead to the failure of the cascade reaction. This reason prevented researchers’ steps from investigating the nature of the cascade reaction in water. To date, only two successful examples in water were reported by our group [44,45], although the alkynoic acids were only limited to linear aliphatic terminal alkynoic acids, amine nucleophiles were only limited to amine nucleophiles carrying a nucleophilic carbon atom, and complicated Au catalysts were required (Scheme 1b). However, considering water often displays unique reactivity and selectivity which can’t be obtained in common organic solvents [46,47,48,49,50,51] and it is an environmentally benign solvent, we aim to develop a more general cascade process between various alkynoic acids and amine nucleophiles in water with great interest. Despite the possibility of hydrolysis of enol lactone intermediate in water at high temperature which may result in the failure of the cascade reaction, we hypothesize that a more general cascade process could also be achieved in water but with a proper catalytic system. In the present study, we develop a greener, and more general and efficient catalytic system (AuPPh_3_Cl/AgSbF_6_/CF_3_CO_2_H) in water, which tolerates a broader substrate scope of alkynoic acids as well as amine nucleophiles. As shown in Scheme 1c, not only linear aliphatic terminal and internal alkynoic acids but also cyclic aromatic terminal and internal alkynoic acids are well tolerated. In addition, extraordinarily broad amine nucleophiles bearing a nucleophilic carbon/nitrogen/oxygen atom turned out to be suitable substrates. Besides, the reaction mechanism in water was carefully checked and studied for the first time. Interestingly, when D_2_O was used as the reaction solvent, we observed the highly deuterated products generated from the H–D exchange between the reaction substrates/intermediates and D_2_O. Herein, we also present the library construction of skeletally diverse *N*-heterocycles embedded with privileged structures employing different alkynoic acids and various amine nucleophiles as the building blocks. To our delight, five antimicrobial compounds were identified from this library after biological evaluation. The production of *N*-heterocycles with diverse scaffolds and the discovery of active pharmaceutical ingredients (APIs) demonstrate the power of this method in both organic synthesis and medicinal chemistry.

## 2. Results and Discussion

4-pentynoic acid (**1a**) and tryptamine (**2a**) were employed as the model substrates to optimize the cascade reaction conditions (Table 1). Pleasingly, treatment of starting materials **1a** and **2a** in water without any catalyst at 100 °C for 24 h gave the desired product **SF1a**, albeit with a low yield (entry 1). Then various metal catalysts were screened to improve the reaction yield. A screening of Pd, Cu, Ni, and Mn complexes (entries 2–11), disclosed, at first, that Pd(II) sources, such as PdCl_2_(CH_3_CN)_2_ and Pd(PPh_3_)_2_Cl_2_, NiOAc and Mn(OAc)_2_, had little enhancement on the yield; while Pd(0) sources such as Pd(PPh_3_)_4_ and Pd_2_(dba)_3_; copper catalysts, such as Cu(OAc)_2_ and Cu(OH)_2_, and CuCl; and Ni(PPh_3_)_2_Cl inhibited this transformation. A subsequent survey of Ag, Ru, and Co catalysts (entries 12–16) revealed that Ag_2_CO_3_, AgOAc, [RuCl_2_(*p*-cym)]_2_, CoCl_2_, and Co(acac)_2_ could obviously increase the yield, affording the product **SF1a** in moderate yields (38–57%). Notably, a good yield (70%) was achieved with AuPPh_3_Cl (entry 17), and increasing the temperature to 120 °C was much more efficient in improving the yield as compared with extending the reaction time to 48 h (entries 18 and 19). Encouraged by this result, an investigation of other Au catalysts at 120 °C was carried out (entries 20–24). Among them, none displayed higher catalytic reactivity than AuPPh_3_Cl. To further improve the yield of the product, a series of silver salts, which were proved to be able to increase the catalytic reactivity of gold catalysts [52,53,54,55,56,57], were screened as the additives (entries 25–28). To our delight, AgSbF_6_ was found to be the best choice, with which product **SF1a** was obtained in 91% yield (entry 28). In addition, other typical organic solvents were also tested as the reaction solvents, and toluene, xylene, and DCE also turned out to be suitable solvents, in which similar high isolated yields were observed (See Supplementary Materials for details). However, considering water is more environmentally benign, we then decided to use water as the solvent to explore the substrate scope of this method. In this way, the optimal reaction conditions were identified using a catalytic system consisting of AuPPh_3_Cl/AgSbF_6_ in water at 120 °C for 24 h.

**Scheme 2:**
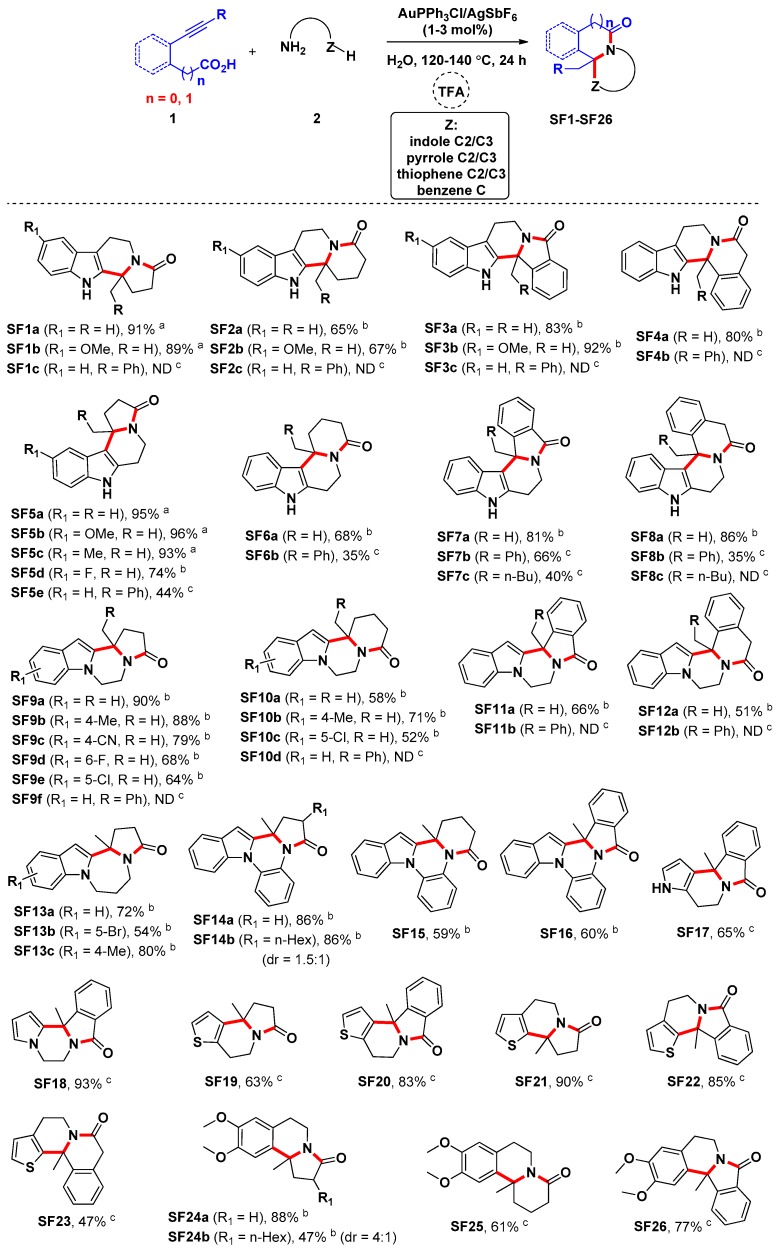
Generation of scaffold diversity. ^a^ Reaction conditions: alkynoic acids **1** (0.6 mmol), amine nucleophiles **2** (0.5 mmol), AuPPh_3_Cl/AgSbF_6_ (0.005 mmol), H_2_O (4.0 mL), and 120 °C, 24 h. ^b^ Reaction conditions: (i) alkynoic acids **1** (0.6 mmol), amine nucleophiles **2** (0.5 mmol), AuPPh_3_Cl/AgSbF_6_ (0.01 mmol), H_2_O (4.0 mL), 120 °C, 20 h; (ii) TFA (0.5 mmol) was added, and then 120 °C, 4 h. ^c^ Reaction conditions: (i) alkynoic acids **1** (0.6 mmol), amine nucleophiles **2** (0.5 mmol), AuPPh_3_Cl/AgSbF_6_ (0.015 mmol), H_2_O (4.0 mL), 140 °C, 20 h; (ii) TFA (0.5 mmol) was added, and then 140 °C, 4 h. ND = Not detected.

After determining the optimal reaction conditions, we then began to construct a high-quality library of privileged substructure-based *N*-heterocycles with diverse scaffolds. We first examined the generality of the process with various amine nucleophiles **2** containing a nucleophilic carbon on a heteroaromatic or aromatic ring. In general, this process tolerated a variety of amine nucleophiles **2** and alkynoic acids **1**, and 26 scaffolds embedded with privileged structures were furnished in good to high yields in water under optimal or modified reaction conditions (Scheme 2). For example, tryptamines reacted smoothly with terminal alkynoic acids such as 4-pentynoic acid under standard conditions to give products **SF1a** and **SF1b** in high yields. The reactions of tryptamines with other terminal alkynoic acids, such as 5-hexynoic acid, 2-ethynylbenzoic acid, and 2-(2-ethynylphenyl)acetic acid, also afforded the corresponding products (**SF2a**, **SF2b**, **SF3a**, **SF3b**, and **SF4a**) in good to high yields under a modified two-step one-pot process, in which CF_3_CO_2_H (TFA) was added to promote the iminium ion formation. In addition, internal alkynoic acids, such as 5-phenylpent-4-ynoic acid, 6-phenylhex-5-ynoic acid, 2-(phenylethynyl)benzoic acid, and 2-(2-(phenylethynyl)phenyl)acetic acid, were also tested as the substrates. Unfortunately, they all failed to react with tryptamines to give the desired products (**SF1c**, **SF2c**, **SF3c**, and **SF4b**) even under further improved conditions. Interestingly, 2-(1*H*-indol-2-yl)ethylamines could undergo this transformation with terminal alkynoic acids as well as internal alkynoic acids to yield the desired products **SF5**–**SF8** in 35–96% yields. It should be noted that no N1 ring closure products were observed when 2-(1*H*-indol-2-yl)ethylamines were used as the amine nucleophiles, suggesting excellent selectivity of this cascade process. This may be because the C3 nucleophilicity is stronger than N1 nucleophilicity [58]. Likewise, the reactivity of 2-(1*H*-indol-1-yl)ethanamines in this cascade reaction was very similar to that of tryptamines. They reacted well with terminal alkynoic acids while their reactions with internal alkynoic acids failed to give the desired products (**SF9**–**SF12**). Interestingly, the protocol was also compatible with 3-(1*H*-indol-1-yl)propan-1-amines, which furnished products **SF13** carrying a seven-membered ring in 54–80% yields. Similarly, products **SF14**–**SF16** were obtained in 59–86% yields when 2-(1*H*-indol-1-yl)anilines and alkynoic acids were subjected to the modified conditions. Notably, the indole-containing polycyclic frameworks, represented by compounds **SF1**–**SF16,** are regarded as valuable N-heterocycles considering their ubiquitous presence in biologically active molecules [59,60,61,62,63]. Subsequently, 2-(1*H*-pyrrol-2-yl)ethanamines, 2-(1*H*-pyrrol-1-yl)ethanamines, 2-(thiophen-2-yl)ethanamines, and 2-(thiophen-3-yl)ethanamines were employed as amine nucleophiles. Their reactions with diverse alkynoic acids took place successfully to provide pyrrole- or thiophene-fused compounds **SF17**–**SF23** in moderate to high yields, despite the fact that stronger conditions were required. In particular, 2-phenylethanamines with electron-donating substituents on the benzene ring were also well tolerated, leading to the formation of benzene-fused heterocyclic products **SF24**–**SF26** in yields ranging from 47 to 88%. It is also worth noting that excellent selectivity was achieved in the reactions of 2-(1*H*-pyrrol-2-yl)ethanamine, 2-(thiophen-3-yl)ethanamine or 2-phenylethanamines, even though two potential cyclization sites existed in the final step. It should be noted that compound **SF26** is the analog of tetrahydroberberines, which were extracted from the Chinese herb *Corydalis ambigua* and exhibited a broad range of biological activities [64,65].

**Scheme 3:**
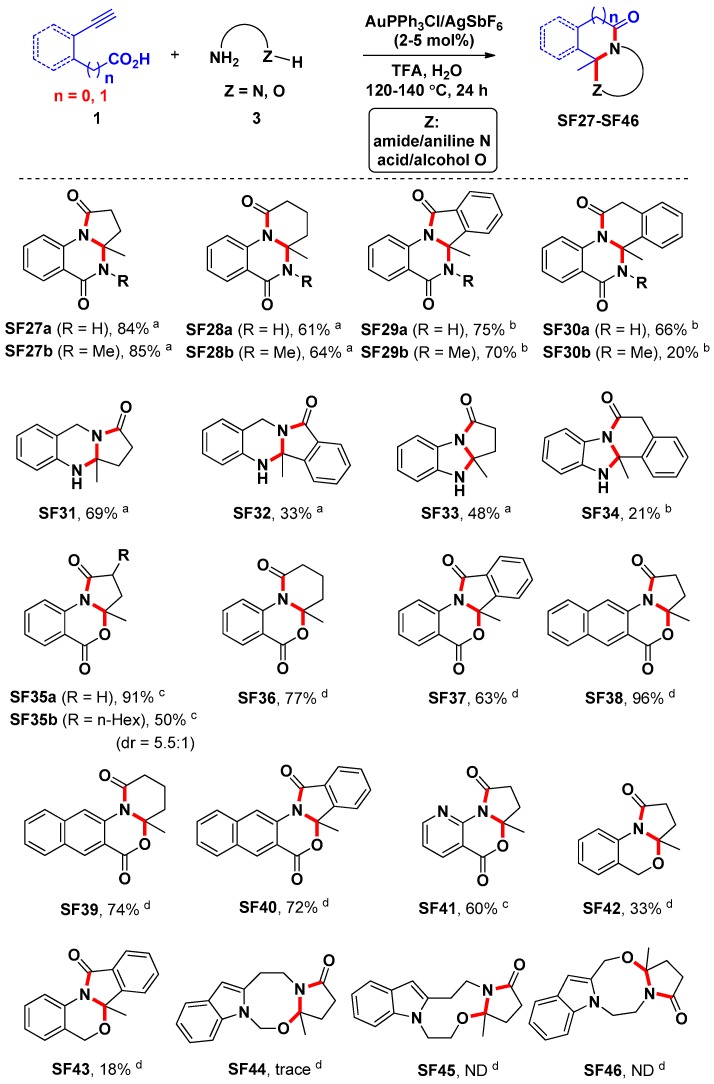
Generation of scaffold diversity. ^a^ Reactions conditions: (i) Alkynoic acids **1** (0.6 mmol), amine nucleophiles **3** (0.5 mmol), AuPPh_3_Cl/AgSbF_6_ (0.01 mmol), H_2_O (4.0 mL), 120 °C, 20 h; (ii) TFA (0.5 mmol) was added, and then 120 °C, 4 h. ^b^ The reaction was carried out at 140 °C. ^c^ The reaction was carried out with 5 mol% AuPPh_3_Cl/AgSbF_6_. ^d^ The reaction was performed with 5 mol% AuPPh_3_Cl/AgSbF_6_ at 140 °C. ND = Not detected.

To further broaden the substrate scope of this approach, amine nucleophiles **3** containing a nucleophilic heteroatom (Z = amide/aniline N, acid/alcohol O) were tested as substrates. Overall, this protocol was also applicable to diverse amine nucleophiles **3**, and 17 scaffolds were constructed with high efficiency (Scheme 3). For instance, the reactions of 2-aminobenzamides with various alkynoic acids worked successfully, affording benzene-fused polycyclic products **SF27**–**SF30** in moderate to high yields. Gratifyingly, this approach was compatible with 2-(aminomethyl)anilines and benzene-1,2-diamines, although the desired benzene-based heterocyclic products **SF31**–**SF34** were obtained in lower yields. Remarkably, in the case of substrates such as 2-aminobenzamides and 2-(aminomethyl)anilines, which contain two nitrogen atoms as the nucleophiles, the nitrogen atom with stronger nucleophilicity tended to attack the enol lactone intermediate and therefore the other nitrogen atom with weaker nucleophilicity attacked the iminium ion intermediate to selectively provide the corresponding products, while not in the reverse way. Besides, 2-aminobenzoic acids, 3-amino-2-naphthoic acids, or 2-aminonicotinic acids reacted with various alkynoic acids smoothly, producing the corresponding benzene-, naphthalene-, or pyridine-fused heterocyclic products **SF35**–**SF41** in yields of 50–96%. Surprisingly, 2-aminobenzyl alcohols were also found to be suitable substrates, which could undergo the cascade reaction with alkynoic acids to give the desired benzene-fused polycyclic products **SF42** and **SF43**, albeit with lower yields. In addition, we also tried to synthesize the indole-fused compounds **SF44**–**SF46** embedded with an eight- or nine-membered ring; unfortunately, we failed. This may be attributed to the low reactivities of the amine nucleophiles and the instability of the large rings in energetics.

**Scheme 4:**
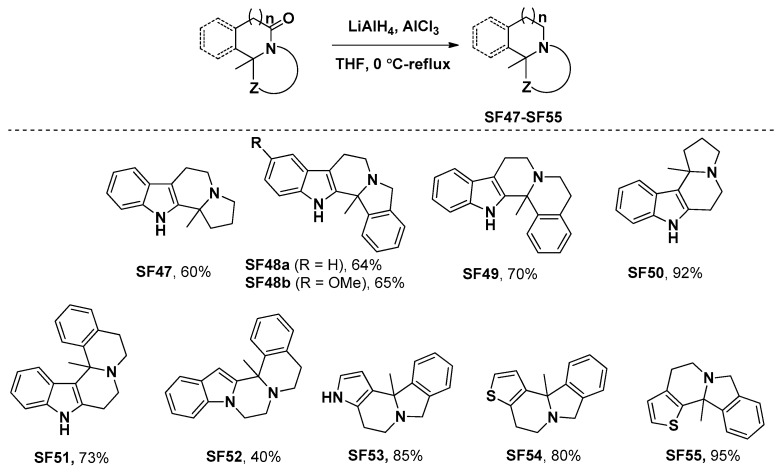
Derivatization of the target compounds in the library.

The diversity of the library can be further expanded by the derivatization of the target compounds. We herein introduce the derivatization of the target compounds based on the simple reduction of the carbonyl group, and the selected results are shown in Scheme 4. Nine different scaffolds represented by indole-, pyrrole-, or thiophene-based polycyclic compounds **SF47**–**SF55** containing a tertiary amine were produced conveniently through an easy reduction of the corresponding precursors with LiAlH_4_/AlCl_3_. Notably, these scaffolds are very similar to those found in natural and pharmaceutical agents [65]. We expect the screening of these compounds towards specific biological targets might lead to the identification of bioactive molecules.

Thus, using various amine nucleophiles and alkynoic acids as the building blocks, a library of privileged substructure-based *N*-heterocycles with diverse scaffolds was constructed through gold catalysis in a green and efficient manner. It is worth noting this cascade process constructs three new bonds together with two rings in one chemical process, suggesting the high efficiency of this cascade reaction in synthesizing nitrogen-containing heterocyclic compounds. Regarding the large occurrence of nitrogen-containing heterocyclic compounds in APIs [66,67,68], the method presented in this paper is prospective since it could provide an environmentally benign and useful platform for the preparation of diverse nitrogen-containing heterocyclic compounds.

To verify our expectation that this approach could provide useful scaffolds with attractive bioactivities, a bioactivity study of this library was carried out. An initial pharmacological study of these nitrogen-containing heterocyclic compounds led to the discovery of five antimicrobial compounds—**SF9d**, **SF29b**, **SF33**, **SF36**, and **SF41**. The minimal inhibitory concentration (MIC) results revealed that compound **SF36** displayed the most potent antibacterial activity against *S. aureus* strain, with a MIC_90_ value of 10–25 μg/mL (Table 2) (Time-kill assays and colony-forming unit studies of compounds **SF9d**, **SF29b**, **SF33**, **SF36** and **SF41** could be found in Supplementary Materials).

**Scheme 5:**
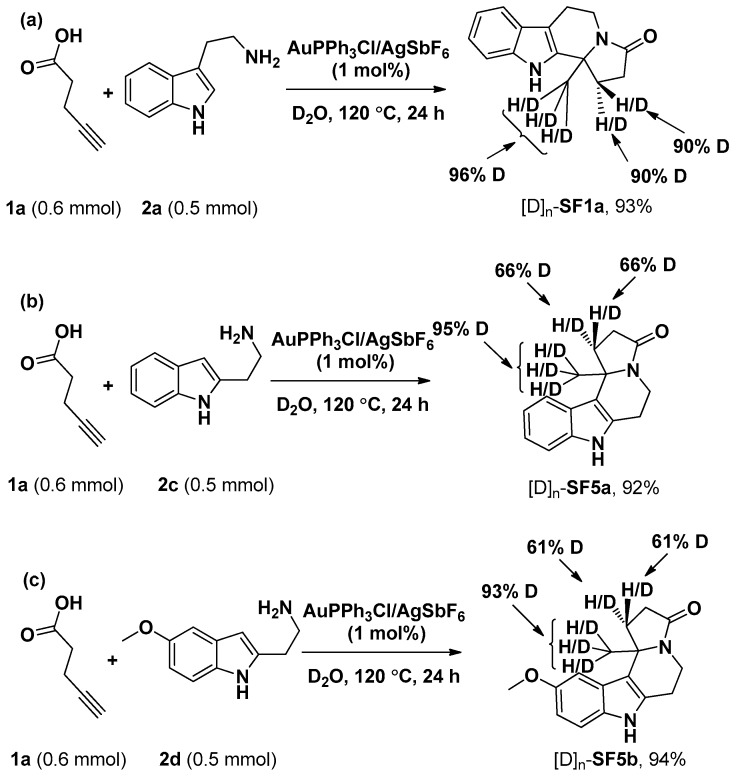
Deuterium-labeling experiments.; (**a**) the deuterium-labeling experiment of substrates **1a** and **2a** in D_2_O; (**b**) the deuterium-labeling experiment of substrates **1a** and **2c** in D_2_O; (**c**) the deuterium-labeling experiment of substrates **1a** and **2d** in D_2_O.

Mechanistic studies were carried out with deuterium-labeling experiments, and the hydrogens of the products were assigned at first by the analysis of the ^1^H-NMR, ^13^C-NMR, HSQC, HMBC, and ^1^H-^1^H COSY spectrum to confirm the deuterated positions (See Supplementary Materials for details). Interestingly, the reaction of 4-pentynoic acid **1a** with tryptamine **2a** in D_2_O under the standard conditions afforded the deuterated product [D]_n_-**SF1a**, not only at the methyl position but also at the β-position of the carbonyl (Scheme 5a). Specifically, a 96% deuteration at the methyl position and a similar deuteration (90%) of the two unequal hydrogens at the β-position of the carbonyl were observed. The reactions of 2-(1*H*-indol-2-yl)ethylamines with 4-pentynoic acid **1a** in D_2_O gave the similar results (Scheme 5b,c).

**Scheme 6:**
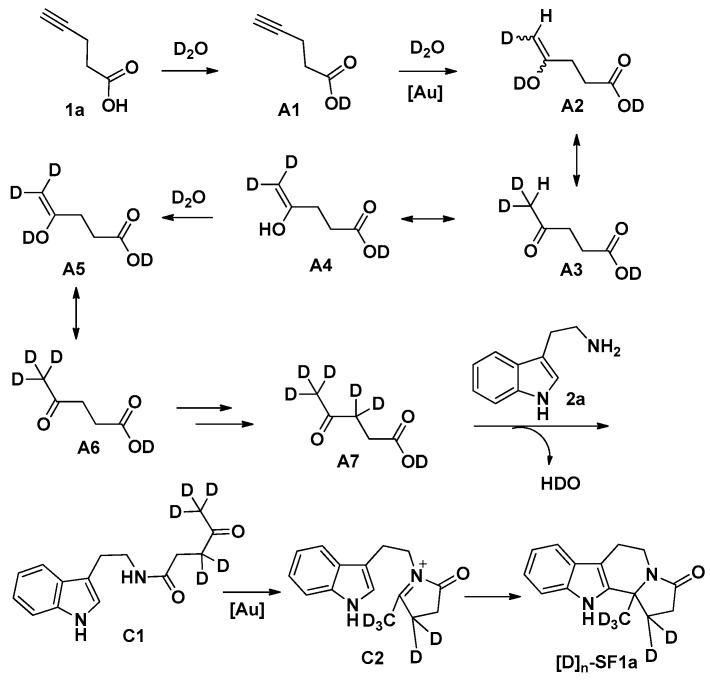
Hypothetic reaction pathway 1.

**Scheme 7:**
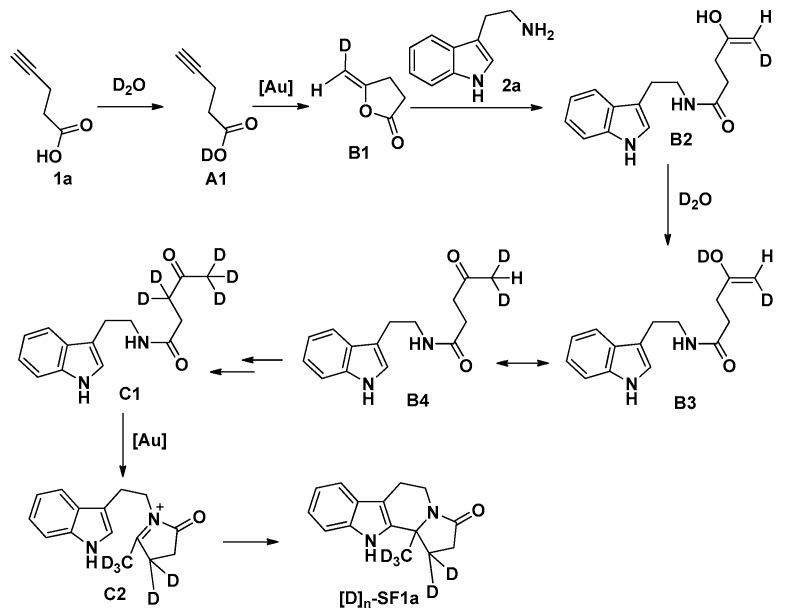
Hypothetic reaction pathway 2.

According to the results of deuterium-labeling experiments, we hypothesize two possible reaction pathways (Scheme 6 and Scheme 7). The reaction of with 4-pentynoic acid **1a** with tryptamine **2a** in the presence of Au catalyst in D_2_O is taken as the example to illustrate the reaction pathway. The first hypothetic reaction pathway (Scheme 6) may involve the gold-catalyzed hydration of carbon–carbon triple bond, which was observed in our previous work [69]. The H–D exchange between the carboxyl group of 4-pentynoic acid **1a** and D_2_O leads to the formation of intermediate **A1**. Gold-catalyzed addition of D_2_O to the carbon–carbon triple bond of intermediate **A1** produces intermediate **A2**, which undergoes two keto–enol tautomerizations to give intermediate **A4**. The subsequent H–D exchange between the hydroxyl group of intermediate **A4** and D_2_O affords intermediate **A5**, which undergoes keto–enol tautomerization again to give intermediate **A6**. The two acidic protons at the α-position of the carbonyl in intermediate **A6** undergo H–D exchange with D_2_O via keto–enol tautomerizations to yield intermediate **A7**. The following condensation between intermediate **A7** and tryptamine **2a**, the subsequent iminium ion formation, and the final cyclization achieve the product [D]_n_-**SF1a**. The second hypothetic reaction pathway is shown in Scheme 7; the H–D exchange between the carboxyl group of 4-pentynoic acid **1a** and D_2_O produces intermediate **A1**. Gold-catalyzed intramolecular cyclization of intermediate **A1** produces enol lactone intermediate **B1**, which is attacked by tryptamine **2a** to give intermediate **B2**. The subsequent H–D exchange between the hydroxyl group of intermediate **B2** and D_2_O yields intermediate **B3**, which undergoes enol–keto tautomerization to provide intermediate **B4**. Similarly, the three acidic protons at the α-position of the carbonyl in intermediate **B4** undergo H–D exchange with D_2_O via keto–enol tautomerizations to yield intermediate **C1**, which is converted into the product [D]_n_-**SF1a** via an iminium ion formation/cyclization sequence.

**Scheme 8:**
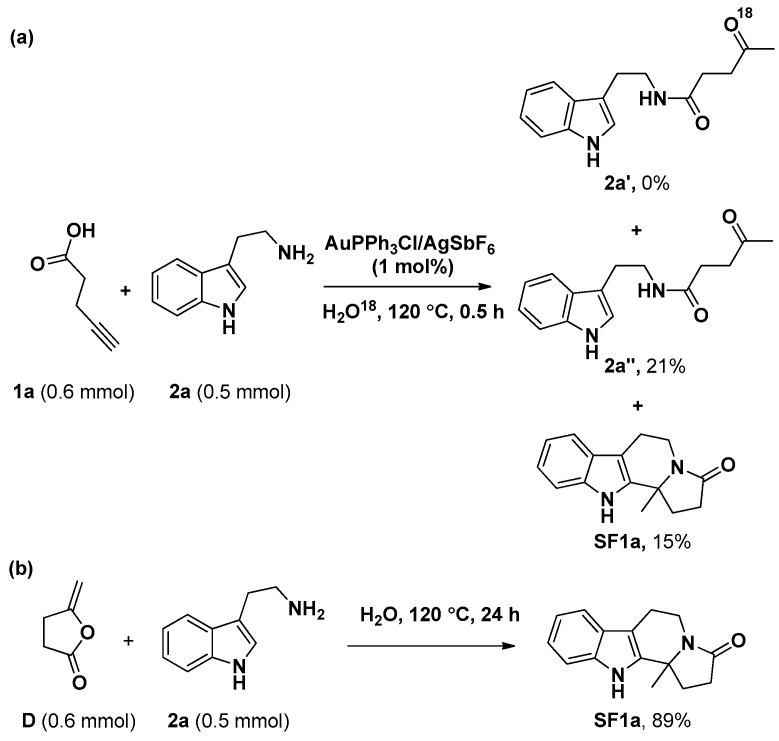
Mechanistic study experiments. (**a**) the mechanistic experiment of substrates **1a** and **2a** in H_2_O^18^; (**b**) the mechanistic experiment of substrates **D** and **2a** without catalyst/additive.

To further verify the reaction mechanism, the reaction of 4-pentynoic acid **1a** and tryptamine **2a** under gold catalysis in O^18^-labeled water was carried out first. This reaction in H_2_O^18^ was stopped after 0.5 h to track the reaction intermediates. As shown in Scheme 8a, apart from the remaining starting materials, intermediate **2a″** and the product **SF1a** were obtained in 21% and 15% yield, respectively. While the O^18^-labeled intermediate **2a′** was not observed. This result clearly indicates the hydration of alkyne moiety, which is proposed in Scheme 6, is not involved. By contrast, the commercially purchased enol lactone **D** reacted smoothly with tryptamine **2a** without the gold catalyst (Scheme 8b). This result shows that the enol lactone species **B1**, which is proposed in Scheme 7, is likely to be the key intermediate.

**Scheme 9:**
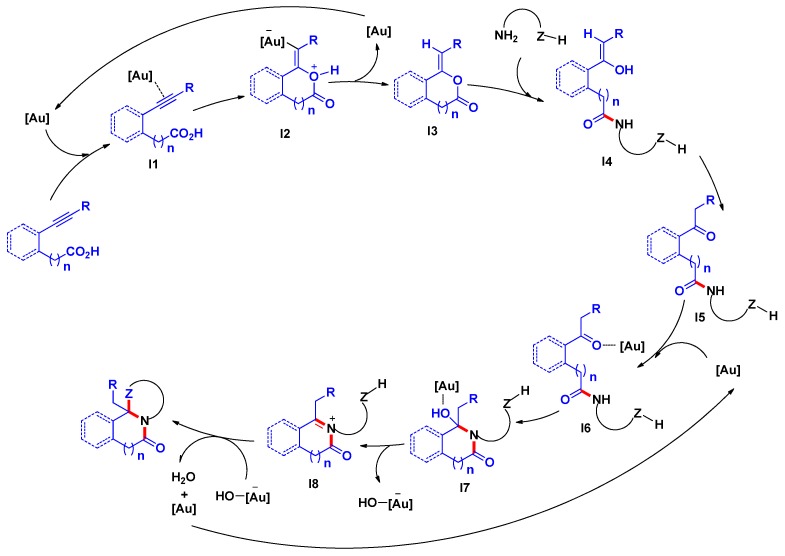
A final proposed reaction mechanism.

On the basis of the above results of mechanistic experiments, a final proposed reaction mechanism is outlined in Scheme 9. The proposed mechanism commences with the coordination of the gold catalyst to the carbon–carbon triple bond of alkynoic acids to produce intermediate **I1**. The subsequent intramolecular *exo* cyclization of **I1** yields intermediate **I2**. The following protodemetalation of intermediate **I2** takes place to produce the enol lactone species **I3** with the regeneration of the catalyst. Then intermediate **I3** undergoes aminolysis by amine nucleophiles to give intermediate **I4**, which tautomerizes to produce intermediate **I5**. Intermediate **I5** is converted into the iminium ion **I8** under the catalysis of the gold catalyst. The final nucleophilic cyclization of intermediate **I8** affords the desired products with the release of the gold catalyst. The stronger nucleophilicity of amine nucleophiles compared to that of H_2_O results in the aminolysis of enol lactone **I3** by amine nucleophiles instead of hydrolysis by H_2_O. It should be noted that the reaction solvent H_2_O participates in this cascade reaction via the H–H exchange with the carboxyl group of alkynoic acids, the hydroxyl group of intermediate **I4**, and the α hydrogen atoms of the carbonyl group of intermediate **I5**, as demonstrated by deuterium-labeling experiments. Besides, TFA could promote this reaction by accelerating the formation of iminium ion **I8**.

## 3. Materials and Methods

### 3.1. General Information

If not otherwise specified, the starting materials were obtained from commercial sources and used directly without purification. Analytical thin-layer chromatography (TLC): HSGF 254 (0.15–0.2 mm thickness). Detection under UV light at 254 nm. Column chromatography: Separations were carried out on silica gel FCP 200–300. Yields refer to isolated compounds. Melting point apparatus: a micro melting point apparatus, values are uncorrected. Nuclear magnetic resonance (NMR) apparatus: a Brucker instrument. Chemical shifts (δ) are given in ppm. Proton coupling patterns were recorded as singlet (s), doublet (d), triplet (t), quartet (q), and multiplet (m). LRMS (low-resolution mass) and HRMS (high-resolution mass) were measured on a spectrometer with an electrospray ionization (ESI) source.

### 3.2. General Procedure for the Preparation of Compounds SF1a, SF1b, SF5a, SF5b, and SF5c

A suspension of alkynoic acids **1** (0.6 mmol), amine nucleophiles **2** (0.5 mmol), and AuPPh_3_Cl/AgSbF_6_ (0.005 mmol) in H_2_O (4.0 mL) was stirred at 120 °C for 24 h. At ambient temperature, saturated Na_2_CO_3_ solution (25.0 mL) was added to the reaction mixture. The resulting mixture was then extracted with ethyl acetate (3 × 15 mL). The combined organic layers were washed with brine, and dried over Na_2_SO_4_. After filtration and removal of the solvents in vacuo, the crude product was purified by flash chromatography on silica gel to provide the desired product.

*11b-Methyl-5,6,11,11b-tetrahydro-1H-indolizino[8,7-b]indol-3(2H)-one* (**SF1a**): white solid (109.7 mg, yield 91%), mp 260–261 °C. ^1^H-NMR (500 MHz, DMSO-*d*_6_) δ 1.54 (s, 3H), 2.07–1.99 (m, 1H), 2.32–2.20 (m, 2H), 2.66–2.55 (m, 2H), 2.75–2.67 (m, 1H), 3.11–3.00 (m, 1H), 4.25–4.16 (m, 1H), 7.01–6.94 (m, 1H), 7.10-7.02 (m, 1H), 7.32 (d, *J* = 8.0 Hz, 1H), 7.39 (d, *J* = 7.8 Hz, 1H), 11.06 (s, 1H); ^13^C-NMR (100 MHz, DMSO-*d*_6_) δ 171.9 (CO), 139.0 (C, Ar), 135.9 (C, Ar), 126.3 (C, Ar), 121.0 (CH, Ar), 118.6 (CH, Ar), 118.0 (CH, Ar), 111.1 (CH, Ar), 104.7 (C, Ar), 58.9 (C), 34.3 (CH_2_), 32.6 (CH_2_), 30.1 (CH_2_), 25.0 (CH_3_), 20.9 (CH_2_); ESI-LRMS *m*/*z*: 241 [M + H]^+^; ESI-HRMS *m*/*z* calcd for M + H^+^ 241.1335, found: 241.1331. The characterization data is in accordance with that reported in [32]. 

*8-Methoxy-11b-methyl-5,6,11,11b-tetrahydro-1H-indolizino[8,7-b]indol-3(2H)-one* (**SF1b**): yellow oil (120.2 mg, yield 89%). ^1^H-NMR (500 MHz, DMSO-*d*_6_) δ 1.53 (s, 3H), 2.07–1.97 (m, 1H), 2.31–2.20 (m, 2H), 2.64–2.52 (m, 2H), 2.73–2.64 (m, 1H), 3.10–2.98 (m, 1H), 3.74 (s, 3H), 4.24–4.15 (m, 1H), 6.70 (dd, *J* = 8.7, 2.4 Hz, 1H), 6.89 (d, *J* = 2.3 Hz, 1H), 7.20 (d, *J* = 8.7 Hz, 1H), 10.88 (s, 1H); ^13^C-NMR (125 MHz, DMSO-*d*_6_) δ 171.9 (CO), 153.2 (C, Ar), 139.7 (C, Ar), 130.9 (C, Ar), 126.6 (C, Ar), 111.7 (CH, Ar), 110.8 (CH, Ar), 104.6 (C, Ar), 100.2 (CH, Ar), 58.9 (C), 55.4 (OCH_3_), 34.3 (CH_2_), 32.6 (CH_2_), 30.1 (CH_2_), 25.0 (CH_3_), 21.0 (CH_2_); ESI-LRMS *m*/*z*: 271 [M + H]^+^; ESI-HRMS *m*/*z* calcd for M + H^+^ 271.1441, found: 271.1437. The characterization data is in accordance with that reported in [43].

*11c-Methyl-5,6,7,11c-tetrahydro-1H-indolizino[7,8-b]indol-3(2H)-one* (**SF5a**): yellow solid (114.2 mg, yield 95%), mp 96–97 °C. ^1^H-NMR (500 MHz, DMSO-*d*_6_) δ 1.54 (s, 3H), 2.02–1.92 (m, 1H), 2.27–2.17 (m, 1H), 2.54–2.47 (m, 1H), 2.65–2.54 (m, 1H), 2.81–2.67 (m, 2H), 3.16–3.04 (m, 1H), 4.27–4.18 (m, 1H), 7.00–6.92 (m, 1H), 7.08–7.00 (m, 1H), 7.30 (d, *J* = 8.0 Hz, 1H), 7.47 (d, *J* = 7.8 Hz, 1H), 10.90 (s, 1H); ^13^C-NMR (100 MHz, DMSO-*d*_6_) δ 171.5 (CO), 135.9 (C, Ar), 130.5 (C, Ar), 123.9 (C, Ar), 120.5 (CH, Ar), 118.6 (CH, Ar), 117.9 (CH, Ar), 115.3 (C, Ar), 111.1 (CH, Ar), 59.2 (C), 33.2 (CH_2_), 33.2 (CH_2_), 30.2 (CH_2_), 25.0 (CH_3_), 22.8 (CH_2_); ESI-LRMS *m*/*z*: 241 [M + H]^+^; ESI-HRMS *m*/*z* calcd for M + H^+^ 241.1335, found: 241.1332. The characterization data is in accordance with that reported in [43].

*10-Methoxy-11c-methyl-5,6,7,11c-tetrahydro-1H-indolizino[7,8-b]indol-3(2H)-one* (**SF5b**): pale yellow solid (129.5 mg, yield 96%), mp 193–194 °C. ^1^H-NMR (500 MHz, DMSO-*d*_6_) δ 1.54 (s, 3H), 2.01–1.91 (m, 1H), 2.27–2.18 (m, 1H), 2.64–2.52 (m, 2H), 2.79–2.65 (m, 2H), 3.14–3.03 (m, 1H), 3.77 (s, 3H), 4.25–4.15 (m, 1H), 6.69 (dd, *J* = 8.7, 2.4 Hz, 1H), 6.93 (d, *J* = 2.3 Hz, 1H), 7.19 (d, *J* = 8.7 Hz, 1H), 10.72 (s, 1H); ^13^C-NMR (100 MHz, DMSO-*d*_6_) δ 171.5 (CO), 153.1 (C, Ar), 131.3 (C, Ar), 131.0 (C, Ar), 124.2 (C, Ar), 115.1 (C, Ar), 111.7 (CH, Ar), 109.8 (CH, Ar), 100.6 (CH, Ar), 59.2 (C), 55.5 (OCH_3_), 33.2 (CH_2_), 33.0 (CH_2_), 30.1 (CH_2_), 24.8 (CH_3_), 22.9 (CH_2_); ESI-LRMS *m*/*z*: 271 [M + H]^+^; ESI-HRMS *m*/*z* calcd for M + H^+^ 271.1441, found: 271.1437. The characterization data is in accordance with that reported in [43].

*10,11c-Dimethyl-5,6,7,11c-tetrahydro-1H-indolizino[7,8-b]indol-3(2H)-one* (**SF5c**): pale yellow oil (118.4 mg, yield 93%). ^1^H-NMR (500 MHz, DMSO-*d*_6_) δ 1.53 (s, 3H), 2.02–1.91 (m, 1H), 2.29–2.17 (m, 1H), 2.38 (s, 3H), 2.50–2.46 (m, 1H), 2.64–2.54 (m, 1H), 2.80–2.65 (m, 2H), 3.15–3.03 (m, 1H), 4.26–4.15 (m, 1H), 6.86 (dd, *J* = 8.2, 1.0 Hz, 1H), 7.18 (d, *J* = 8.2 Hz, 1H), 7.25 (s, 1H), 10.75 (s, 1H); ^13^C-NMR (125 MHz, DMSO-*d*_6_) δ 171.5 (CO), 134.2 (C, Ar), 130.5 (C, Ar), 127.1 (C, Ar), 124.1 (C, Ar), 122.0 (CH, Ar), 117.6 (CH, Ar), 114.8 (C, Ar), 110.8 (CH, Ar), 59.2 (C), 33.3 (CH_2_), 33.2 (CH_2_), 30.2 (CH_2_), 25.0 (CH_3_), 22.8 (CH_2_), 21.3 (CH_3_); ESI-LRMS *m*/*z*: 255 [M + H]^+^; ESI-HRMS *m*/*z* calcd for M + H^+^ 255.1492, found: 255.1489. The characterization data is in accordance with that reported in [43].

### 3.3. General Procedure for the Preparation of Compounds ***SF1c**, **SF2**–**SF4**, **SF5d**, **SF5e*** and ***SF6**–**SF46***

A suspension of alkynoic acids **1** (0.6 mmol), amine nucleophiles **2** or **3** (0.5 mmol), and AuPPh_3_Cl/AgSbF_6_ (with the amount indicated) in H_2_O (4.0 mL) was stirred at the temperature indicated for 20 h. Then the reaction mixture was cooled to room temperature, and CF_3_COOH (0.5 mmol) was added, and the resulting mixture was stirred for another 4 h at the temperature indicated. At ambient temperature, saturated Na_2_CO_3_ solution (25.0 mL) was added to the reaction mixture. The resulting mixture was then extracted with ethyl acetate (3 × 15 mL). The combined organic layers were washed with brine, and dried over Na_2_SO_4_. After filtration and removal of the solvents in vacuo, the crude product was purified by flash chromatography on silica gel to yield the desired product.

*12b-Methyl-1,2,3,6,7,12b-hexahydroindolo[2,3-a]quinolizin-4(12H)-one* (**SF2a**): white solid (82.6 mg, yield 65%), mp 255–256 °C. ^1^H-NMR (500 MHz, DMSO-*d*_6_) δ 1.60 (s, 3H), 1.80–1.68 (m, 2H), 1.97–1.86 (m, 1H), 2.32–2.21 (m, 1H), 2.45–2.32 (m, 2H), 2.61–2.53 (m, 1H), 2.69–2.62 (m, 1H), 2.99–2.87 (m, 1H), 4.90–4.81 (m, 1H), 7.00–6.93 (m, 1H), 7.10–7.02 (m, 1H), 7.32 (d, *J* = 8.0 Hz, 1H), 7.39 (d, *J* = 7.8 Hz, 1H), 10.92 (s, 1H); ^13^C-NMR (125 MHz, DMSO-*d*_6_) δ 167.9 (CO), 139.7 (C, Ar), 136.0 (C, Ar), 126.2 (C, Ar), 120.9 (CH, Ar), 118.5 (CH, Ar), 117.9 (CH, Ar), 111.1 (CH, Ar), 105.8 (C, Ar), 56.4 (C), 35.6 (CH_2_), 34.8 (CH_2_), 31.8 (CH_2_), 25.3 (CH_3_), 21.0 (CH_2_), 16.3 (CH_2_); ESI-LRMS *m*/*z*: 255 [M + H]^+^; ESI-HRMS *m*/*z* calcd for M + H^+^ 255.1492, found: 255.1488. The characterization data is in accordance with that reported in [43].

*9-Methoxy-12b-methyl-1,2,3,6,7,12b-hexahydroindolo[2,3-a]quinolizin-4(12H)-one* (**SF2b**): pale yellow solid (95.4 mg, yield 67%), mp 190–191 °C. ^1^H-NMR (400 MHz, DMSO-*d*_6_) δ 1.59 (s, 3H), 1.79–1.66 (m, 2H), 1.97–1.84 (m, 1H), 2.42–2.21 (m, 3H), 2.66–2.51 (m, 2H), 2.99–2.86 (m, 1H), 3.74 (s, 3H), 4.90–4.79 (m, 1H), 6.70 (dd, *J* = 8.7, 2.4 Hz, 1H), 6.89 (d, *J* = 2.4 Hz, 1H), 7.20 (d, *J* = 8.7 Hz, 1H), 10.73 (s, 1H); ^13^C-NMR (125 MHz, DMSO-*d*_6_) δ 167.9 (CO), 153.2 (C, Ar), 140.4 (C, Ar), 131.0 (C, Ar), 126.5 (C, Ar), 111.7 (CH, Ar), 110.7 (CH, Ar), 105.7 (C, Ar), 100.1 (CH, Ar), 56.4 (C), 55.4 (OCH_3_), 35.6 (CH_2_), 34.8 (CH_2_), 31.8 (CH_2_), 25.4 (CH_3_), 21.1 (CH_2_), 16.3 (CH_2_); ESI-LRMS *m*/*z*: 285 [M + H]^+^; ESI-HRMS *m*/*z* calcd for M + H^+^ 285.1598, found: 285.1593. The characterization data is in accordance with that reported in [43].

*13b-Methyl-7,8,13,13b-tetrahydro-5H-benzo[1,2]indolizino[8,7-b]indol-5-one* (**SF3a**): white solid (119.4 mg, yield 83%), mp 283–284 °C. ^1^H-NMR (400 MHz, DMSO-*d*_6_) δ 1.86 (s, 3H), 2.75–2.63 (m, 1H), 2.85–2.75 (m, 1H), 3.47–3.36 (m, 1H), 4.59–4.47 (m, 1H), 7.03–6.93 (m, 1H), 7.15–7.05 (m, 1H), 7.44–7.34 (m, 2H), 7.58–7.49 (m, 1H), 7.79–7.68 (m, 2H), 8.32 (d, *J* = 7.9 Hz, 1H), 11.35 (s, 1H); ^13^C-NMR (100 MHz, DMSO-*d*_6_) δ 167.2 (CO), 149.3 (C, Ar), 136.2 (C, Ar), 135.2 (C, Ar), 132.2 (CH, Ar), 130.3 (C, Ar), 128.6 (CH, Ar), 126.0 (C, Ar), 123.2 (CH, Ar), 122.8 (CH, Ar), 121.6 (CH, Ar), 118.9 (CH, Ar), 118.3 (CH, Ar), 111.2 (CH, Ar), 106.3 (C, Ar), 62.0 (C), 35.4 (CH_2_), 25.9 (CH_3_), 21.4 (CH_2_); ESI-LRMS *m*/*z*: 289 [M + H]^+^; ESI-HRMS *m*/*z* calcd for M + H^+^ 289.1335, found: 289.1330. The characterization data is in accordance with that reported in [43].

*10-Methoxy-13b-methyl-7,8,13,13b-tetrahydro-5H-benzo[1,2]indolizino[8,7-b]indol-5-one* (**SF3b**): white solid (146.9 mg, yield 92%), mp 164–165 °C. ^1^H-NMR (400 MHz, DMSO-*d*_6_) δ 1.84 (s, 3H), 2.72–2.60 (m, 1H), 2.81–2.73 (m, 1H), 3.45–3.35 (m, 1H), 3.73 (s, 3H), 4.57–4.45 (m, 1H), 6.73 (dd, *J* = 8.7, 2.1 Hz, 1H), 6.89 (d, *J* = 2.1 Hz, 1H), 7.26 (d, *J* = 8.7 Hz, 1H), 7.58–7.47 (m, 1H), 7.77–7.67 (m, 2H), 8.29 (d, *J* = 7.9 Hz, 1H), 11.18 (s, 1H); ^13^C-NMR (125 MHz, DMSO-*d*_6_) δ 167.2 (CO), 153.3 (C, Ar), 149.4 (C, Ar), 135.8 (C, Ar), 132.2 (CH, Ar), 131.2 (C, Ar), 130.2 (C, Ar), 128.6 (CH, Ar), 126.3 (C, Ar), 123.2 (CH, Ar), 122.8 (CH, Ar), 111.9 (CH, Ar), 111.5 (CH, Ar), 106.2 (C, Ar), 100.3 (CH, Ar), 62.0 (C), 55.4 (OCH_3_), 35.5 (CH_2_), 26.0 (CH_3_), 21.5 (CH_2_); ESI-LRMS *m*/*z*: 319 [M + H]^+^; ESI-HRMS *m*/*z* calcd for M + H^+^ 319.1441, found: 319.1435. The characterization data is in accordance with that reported in [43].

*14b-Methyl-8,9,14,14b-tetrahydroindolo[2′,3′:3,4]pyrido[2,1-a]isoquinolin-6(5H)-one* (**SF4a**): pale yellow solid (120.7 mg, yield 80%), mp 137–138 °C. ^1^H-NMR (400 MHz, DMSO-*d*_6_) δ 1.84 (s, 3H), 2.50–2.41 (m, 1H), 2.86–2.75 (m, 1H), 2.96–2.86 (m, 1H), 3.63 (d, *J* = 19.4 Hz, 1H), 4.08 (d, *J* = 19.2 Hz, 1H), 4.96–4.85 (m, 1H), 7.08–6.98 (m, 1H), 7.18–7.12 (m, 1H), 7.24–7.18 (m, 1H), 7.31–7.24 (m, 2H), 7.52–7.43 (m, 3H), 11.56 (s, 1H); ^13^C-NMR (100 MHz, DMSO-*d*_6_) δ169.0 (CO), 139.9 (C, Ar), 136.1 (C, Ar), 135.1 (C, Ar), 132.3 (C, Ar), 127.8 (CH, Ar), 127.5 (CH, Ar), 126.4 (CH, Ar), 126.0 (C, Ar), 124.1 (CH, Ar), 121.4 (CH, Ar), 118.8 (CH, Ar), 118.2 (CH, Ar), 111.3 (CH, Ar), 109.2 (C, Ar), 60.9 (C), 38.0 (CH_2_), 37.9 (CH_2_), 26.2 (CH_3_), 21.0 (CH_2_); ESI-LRMS *m*/*z*: 303 [M + H]^+^; ESI-HRMS *m*/*z* calcd for M + H^+^ 303.1492, found: 303.1487. The characterization data is in accordance with that reported in [43].

*10-Fluoro-11c-methyl-5,6,7,11c-tetrahydro-1H-indolizino[7,8-b]indol-3(2H)-one* (**SF5d**): pale yellow solid (95.1 mg, yield 74%), mp 104–105 °C. ^1^H-NMR (400 MHz, DMSO-*d*_6_) δ 1.52 (s, 3H), 1.99–1.87 (m, 1H), 2.28–2.16 (m, 1H), 2.64–2.51 (m, 2H), 2.83–2.67 (m, 2H), 3.16–3.01 (m, 1H), 4.28–4.15 (m, 1H), 6.93–6.82 (m, 1H), 7.34–7.21 (m, 2H), 11.00 (s, 1H); ^13^C-NMR (100 MHz, DMSO-*d*_6_) δ 171.5 (CO), 156.7 (d, *J_C–F_* = 231.1 Hz, CF, Ar), 132.9 (C, Ar), 132.5 (C, Ar), 124.0 (d, *J_C–F_* = 10.1 Hz, C, Ar), 115.7 (d, *J_C–F_* = 4.5 Hz, C, Ar), 111.9 (d, *J_C–F_* = 9.9 Hz, CH, Ar), 108.3 (d, *J_C–F_* = 25.8 Hz, CH, Ar), 102.9 (d, *J_C–F_* = 23.4 Hz, CH, Ar), 59.0 (C), 33.1 (CH_2_), 32.9 (CH_2_), 30.1 (CH_2_), 24.8 (CH_3_), 22.9 (CH_2_); ESI-LRMS *m*/*z*: 259 [M + H]^+^; ESI-HRMS *m*/*z* calcd for M + H^+^ 259.1241, found: 259.1238. The characterization data is in accordance with that reported in [43].

*11c-Benzyl-5,6,7,11c-tetrahydro-1H-indolizino[7,8-b]indol-3(2H)-one* (**SF5e**): pale yellow oil (69.4 mg, yield 44%). ^1^H-NMR (500 MHz, CDCl_3_) δ 1.78–1.65 (m, 1H), 2.22–2.09 (m, 2H), 2.72–2.60 (m, 2H), 2.97–2.85 (m, 1H), 3.08–2.98 (m, 1H), 3.21 (d, *J* = 13.9 Hz, 1H), 3.30 (d, *J* = 13.9 Hz, 1H), 4.50 (dd, *J* = 12.9, 6.4 Hz, 1H), 7.12–7.06 (m, 2H), 7.22–7.13 (m, 2H), 7.26–7.23 (m, 3H), 7.35 (d, *J* = 8.0 Hz, 1H), 7.52 (d, *J* = 7.6 Hz, 1H), 8.33 (s, 1H); ^13^C-NMR (125 MHz, CDCl_3_) δ 174.3 (CO), 136.7 (C, Ar), 136.2 (C, Ar), 130.6 (C, Ar), 130.2 (2 × CH, Ar), 128.5 (2 × CH, Ar), 127.0 (CH, Ar), 124.4 (C, Ar), 121.8 (CH, Ar), 119.9 (CH, Ar), 118.5 (CH, Ar), 115.9 (C, Ar), 111.3 (CH, Ar), 63.8 (C), 44.6 (CH_2_), 34.3 (CH_2_), 31.6 (CH_2_), 31.0 (CH_2_), 23.1 (CH_2_); ESI-LRMS *m*/*z*: 317 [M + H]^+^; ESI-HRMS *m*/*z* calcd for M + H^+^ 317.1648, found: 317.1651. The characterization data is in accordance with that reported in [43].

*12c-Methyl-1,2,3,6,7,12c-hexahydroindolo[3,2-a]quinolizin-4(8H)-one* (**SF6a**): white solid (86.8 mg, yield 68%), mp 132–133 °C. ^1^H-NMR (400 MHz, DMSO-*d*_6_) δ 1.63 (s, 3H), 1.77–1.65 (m, 2H), 2.01–1.85 (m, 1H), 2.32–2.19 (m, 1H), 2.43–2.32 (m, 1H), 2.78–2.60 (m, 3H), 3.01–2.89 (m, 1H), 4.92–4.80 (m, 1H), 6.99–6.90 (m, 1H), 7.07–6.99 (m, 1H), 7.29 (d, *J* = 8.0 Hz, 1H), 7.51 (d, *J* = 7.8 Hz, 1H), 10.91 (s, 1H); ^13^C-NMR (125 MHz, DMSO-*d*_6_) δ 168.1 (CO), 136.1 (C, Ar), 131.9 (C, Ar), 124.0 (C, Ar), 120.3 (CH, Ar), 118.6 (CH, Ar), 118.5 (CH, Ar), 115.7 (C, Ar), 111.1 (CH, Ar), 57.0 (C), 35.6 (CH_2_), 34.7 (CH_2_), 31.9 (CH_2_), 25.1 (CH_3_), 23.3 (CH_2_), 16.5 (CH_2_); ESI-LRMS *m*/*z*: 255 [M + H]^+^; ESI-HRMS *m*/*z* calcd for M + H^+^ 255.1492, found: 255.1488. The characterization data is in accordance with that reported in [43].

*12c-Benzyl-1,2,3,6,7,12c-hexahydroindolo[3,2-a]quinolizin-4(8H)-one* (**SF6b**): pale yellow oil (57.9 mg, yield 35%). ^1^H-NMR (400 MHz, DMSO-*d*_6_) δ 1.52–1.41 (m, 1H), 1.68–1.54 (m, 1H), 1.90–1.78 (m, 1H), 2.24–2.05 (m, 2H), 2.61–2.53 (m, 1H), 2.81–2.64 (m, 3H), 3.24 (d, *J* = 13.6 Hz, 1H), 3.41 (d, *J* = 13.5 Hz, 1H), 4.85–4.74 (m, 1H), 6.94–6.86 (m, 1H), 7.06–6.96 (m, 3H), 7.23–7.14 (m, 3H), 7.29 (d, *J* = 8.0 Hz, 1H), 7.33 (d, *J* = 7.9 Hz, 1H), 10.95 (s, 1H); ^13^C-NMR (150 MHz, DMSO-*d*_6_) δ 169.2 (CO), 137.8 (C, Ar), 136.0 (C, Ar), 133.0 (C, Ar), 130.4 (2 × CH, Ar), 127.9 (2 × CH, Ar), 126.4 (CH, Ar), 124.3 (C, Ar), 120.2 (CH, Ar), 119.0 (CH, Ar), 118.5 (CH, Ar), 114.2 (C, Ar), 111.1 (CH, Ar), 60.9 (C), 44.3 (CH_2_), 35.0 (CH_2_), 33.9 (CH_2_), 31.7 (CH_2_), 23.0 (CH_2_), 16.3 (CH_2_); ESI-LRMS *m*/*z*: 331 [M + H]^+^; ESI-HRMS *m*/*z* calcd for M + H^+^ 331.1805, found: 331.1812. The characterization data is in accordance with that reported in [43].

*13b-Methyl-6,7-dihydro-5H-benzo[1,2]indolizino[7,8-b]indol-9(13bH)-one* (**SF7a**): pale yellow solid (116.4 mg, yield 81%), mp 235–236 °C. ^1^H-NMR (500 MHz, DMSO-*d*_6_) δ 1.90 (s, 3H), 2.80–2.70 (m, 1H), 2.91–2.80 (m, 1H), 3.45–3.37 (m, 1H), 4.56–4.46 (m, 1H), 7.12–7.03 (m, 2H), 7.34–7.26 (m, 1H), 7.52–7.44 (m, 1H), 7.69–7.64 (m, 1H), 7.71 (d, *J* = 7.5 Hz, 1H), 8.12–8.03 (m, 1H), 8.27 (d, *J* = 7.8 Hz, 1H), 11.10 (s, 1H); ^13^C-NMR (125 MHz, DMSO-*d*_6_) δ 167.3 (CO), 151.4 (C, Ar), 135.9 (C, Ar), 132.3 (C, Ar), 132.0 (CH, Ar), 130.2 (C, Ar), 128.1 (CH, Ar), 124.3 (C, Ar), 123.2 (CH, Ar), 123.1 (CH, Ar), 120.7 (CH, Ar), 119.0 (CH, Ar), 119.0 (CH, Ar), 111.6 (C, Ar), 111.3 (CH, Ar), 63.6 (C), 34.6 (CH_2_), 25.7 (CH_3_), 23.4 (CH_2_); ESI-LRMS *m*/*z*: 289 [M + H]^+^; ESI-HRMS *m*/*z* calcd for M + H^+^ 289.1335, found: 289.1330. The characterization data is in accordance with that reported in [43].

*13b-Benzyl-6,7-dihydro-5H-benzo[1,2]indolizino[7,8-b]indol-9(13bH)-one* (**SF7b**): white solid (121.1 mg, yield 66%), mp 281–282 °C. ^1^H-NMR (500 MHz, DMSO-*d*_6_) δ 2.91–2.72 (m, 2H), 3.49–3.38 (m, 1H), 3.57 (d, *J* = 13.8 Hz, 1H), 3.85 (d, *J* = 13.9 Hz, 1H), 4.53 (dd, *J* = 13.0, 5.6 Hz, 1H), 6.95–6.87 (m, 2H), 7.08–6.98 (m, 3H), 7.20–7.08 (m, 2H), 7.39–7.30 (m, 2H), 7.46 (d, *J* = 7.4 Hz, 1H), 7.66–7.58 (m, 1H), 8.32 (d, *J* = 7.8 Hz, 1H), 8.43 (d, *J* = 7.8 Hz, 1H), 11.14 (s, 1H); ^13^C-NMR (125 MHz, DMSO-*d*_6_) δ 167.7 (CO), 148.8 (C, Ar), 135.9 (C, Ar), 135.7 (C, Ar), 132.8 (C, Ar), 131.4 (CH, Ar), 131.0 (C, Ar), 130.0 (2 × CH, Ar), 127.8 (CH, Ar), 127.3 (2 × CH, Ar), 126.2 (CH, Ar), 124.4 (C, Ar), 123.9 (CH, Ar), 122.6 (CH, Ar), 120.7 (CH, Ar), 119.4 (CH, Ar), 119.1 (CH, Ar), 111.3 (CH, Ar), 111.1 (C, Ar), 67.1 (C), 42.7 (CH_2_), 34.8 (CH_2_), 23.4 (CH_2_); ESI-LRMS *m*/*z*: 365 [M + H]^+^; ESI-HRMS *m*/*z* calcd for M + H^+^ 365.1648, found: 365.1649. The characterization data is in accordance with that reported in [43].

*13b-Pentyl-6,7-dihydro-5H-benzo[1,2]indolizino[7,8-b]indol-9(13bH)-one* (**SF7c**): pale yellow oil (68.7 mg, yield 40%). ^1^H-NMR (400 MHz, CDCl_3_) δ 0.77 (t, *J* = 6.7 Hz, 3H), 0.89–0.83 (m, 1H), 1.20–1.02 (m, 5H), 2.24–2.10 (m, 1H), 2.76–2.58 (m, 2H), 3.13–2.98 (m, 1H), 3.38–3.24 (m, 1H), 4.77 (dd, *J* = 13.1, 6.1 Hz, 1H), 7.23–7.12 (m, 2H), 7.31 (d, *J* = 7.8 Hz, 1H), 7.45–7.38 (m, 1H), 7.62–7.54 (m, 1H), 7.85 (d, *J* = 7.5 Hz, 1H), 7.99 (d, *J* = 7.7 Hz, 1H), 8.06 (d, *J* = 7.7 Hz, 1H), 8.23 (s, 1H); ^13^C-NMR (150 MHz, CDCl_3_) δ 169.4 (CO), 149.6 (C, Ar), 136.1 (C, Ar), 132.0 (CH, Ar), 131.9 (C, Ar), 131.8 (C, Ar), 128.1 (CH, Ar), 124.9 (C, Ar), 123.9 (CH, Ar), 122.9 (CH, Ar), 121.7 (CH, Ar), 120.0 (CH, Ar), 119.4 (CH, Ar), 113.5 (C, Ar), 111.4 (CH, Ar), 67.4 (C), 38.2 (CH_2_), 35.3 (CH_2_), 31.8 (CH_2_), 24.1 (CH_2_), 23.1 (CH_2_), 22.5 (CH_2_), 14.1 (CH_3_); ESI-LRMS *m*/*z*: 345 [M + H]^+^; ESI-HRMS *m*/*z* calcd for M + H^+^ 345.1961, found: 345.1966. The characterization data is in accordance with that reported in [43].

*14c-Methyl-8,9,10,14c-tetrahydroindolo[3′,2’:3,4]pyrido[2,1-a]isoquinolin-6(5H)-one* (**SF8a**): yellow solid (129.8 mg, yield 86%), mp 250–251 °C. ^1^H-NMR (400 MHz, DMSO-*d*_6_) δ 1.89 (s, 3H), 2.72–2.60 (m, 1H), 2.84–2.73 (m, 1H), 3.02–2.89 (m, 1H), 3.62 (d, *J* = 19.4 Hz, 1H), 4.10 (d, *J* = 19.2 Hz, 1H), 4.95–4.86 (m, 1H), 7.15–7.04 (m, 3H), 7.30–7.19 (m, 2H), 7.44–7.38 (m, 2H), 7.59 (d, *J* = 7.6 Hz, 1H), 11.26 (s, 1H); ^13^C-NMR (100 MHz, DMSO-*d*_6_) δ 169.3 (CO), 141.4 (C, Ar), 136.0 (C, Ar), 134.5 (C, Ar), 132.4 (C, Ar), 127.7 (CH, Ar), 127.1 (CH, Ar), 126.2 (C, Ar), 126.1 (CH, Ar), 124.7 (CH, Ar), 120.5 (CH, Ar), 120.2 (CH, Ar), 119.2 (CH, Ar), 111.5 (CH, Ar), 111.2 (C, Ar), 61.5 (C), 38.2 (CH_2_), 36.9 (CH_2_), 25.7 (CH_3_), 23.3 (CH_2_); ESI-LRMS *m*/*z*: 303 [M + H]^+^; ESI-HRMS *m*/*z* calcd for M + H^+^ 303.1492, found: 303.1486. The characterization data is in accordance with that reported in [43].

*14c-Benzyl-8,9,10,14c-tetrahydroindolo[3’,2’:3,4]pyrido[2,1-a]isoquinolin-6(5H)-one* (**SF8b**): pale yellow oil (66.5 mg, yield 35%). ^1^H-NMR (500 MHz, CDCl_3_) δ 1.84–1.73 (m, 1H), 2.48–2.38 (m, 1H), 2.67–2.56 (m, 1H), 3.40 (d, *J* = 13.8 Hz, 1H), 3.96–3.76 (m, 3H), 4.79 (dd, *J* = 12.7, 4.3 Hz, 1H), 6.80–6.71 (m, 2H), 7.08–7.00 (m, 2H), 7.16–7.08 (m, 2H), 7.28–7.20 (m, 2H), 7.33–7.28 (m, 2H), 7.50–7.42 (m, 1H), 7.66 (d, *J* = 7.9 Hz, 1H), 7.96–7.88 (m, 1H), 8.36 (s, 1H); ^13^C-NMR (125 MHz, CDCl_3_) δ 170.6 (CO), 141.0 (C, Ar), 136.4 (C, Ar), 136.1 (C, Ar), 135.7 (C, Ar), 132.3 (C, Ar), 130.4 (2 × CH, Ar), 128.2 (2 × CH, Ar), 128.0 (CH, Ar), 127.7 (CH, Ar), 127.3 (C, Ar), 126.9 (CH, Ar), 126.8 (CH, Ar), 126.0 (CH, Ar), 121.6 (CH, Ar), 121.4 (CH, Ar), 120.5 (CH, Ar), 111.7 (CH, Ar), 109.9 (C, Ar), 66.5 (C), 45.0 (CH_2_), 39.1 (CH_2_), 38.7 (CH_2_), 23.3 (CH_2_); ESI-LRMS *m*/*z*: 379 [M + H]^+^; ESI-HRMS *m*/*z* calcd for M + H^+^ 379.1805, found: 379.1815. The characterization data is in accordance with that reported in [43].

*12b-Methyl-1,5,6,12b-tetrahydropyrrolo[2’,1’:3,4]pyrazino[1,2-a]indol-3(2H)-one* (**SF9a**): white solid (107.8 mg, yield 90%), mp 121–122 °C. ^1^H-NMR (400 MHz, DMSO-*d*_6_) δ 1.59 (s, 3H), 2.33–2.17 (m, 2H), 2.47–2.36 (m, 1H), 2.65–2.53 (m, 1H), 3.49–3.38 (m, 1H), 3.81–3.68 (m, 1H), 4.34–4.22 (m, 2H), 6.34 (s, 1H), 7.08–7.01 (m, 1H), 7.15–7.09 (m, 1H), 7.38 (d, *J* = 8.1 Hz, 1H), 7.51 (d, *J* = 7.7 Hz, 1H); ^13^C-NMR (125 MHz, DMSO-*d*_6_) δ 172.1 (CO), 142.1 (C, Ar), 135.2 (C, Ar), 127.6 (C, Ar), 120.7 (CH, Ar), 119.9 (CH, Ar), 119.8 (CH, Ar), 109.6 (CH, Ar), 95.5 (CH, Ar), 58.6 (C), 40.9 (CH_2_), 34.3 (CH_2_), 33.6 (CH_2_), 29.7 (CH_2_), 27.2 (CH_3_); ESI-LRMS *m*/*z*: 241 [M + H]^+^; ESI-HRMS *m*/*z* calcd for M + H^+^ 241.1335, found: 241.1330. The characterization data is in accordance with that reported in [43].

*11,12b-Dimethyl-1,5,6,12b-tetrahydropyrrolo[2’,1’:3,4]pyrazino[1,2-a]indol-3(2H)-one* (**SF9b**): pale yellow oil (111.3 mg, yield 88%). ^1^H-NMR (300 MHz, CDCl_3_) δ 1.65 (s, 3H), 2.49–2.37 (m, 3H), 2.54 (s, 3H), 2.73–2.57 (m, 1H), 3.47–3.32 (m, 1H), 3.96–3.81 (m, 1H), 4.20 (dd, *J* = 11.7, 4.7 Hz, 1H), 4.52 (dd, *J* = 13.6, 5.1 Hz, 1H), 6.30 (s, 1H), 6.98–6.89 (m, 1H), 7.15–7.09 (m, 2H); ^13^C-NMR (100 MHz, CDCl_3_) δ 173.2 (CO), 141.1 (C, Ar), 135.4 (C, Ar), 130.0 (C, Ar), 127.9 (C, Ar), 121.8 (CH, Ar), 120.7 (CH, Ar), 106.8 (CH, Ar), 94.8 (CH, Ar), 59.5 (C), 41.3 (CH_2_), 35.0 (CH_2_), 34.3 (CH_2_), 30.4 (CH_2_), 27.8 (CH_3_), 18.8 (CH_3_); ESI-LRMS *m*/*z*: 255 [M + H]^+^; ESI-HRMS *m*/*z* calcd for M + H^+^ 255.1492, found: 255.1488. The characterization data is in accordance with that reported in [45].

*12b-Methyl-3-oxo-1,2,3,5,6,12b-hexahydropyrrolo[2’,1’:3,4]pyrazino[1,2-a]indole-11-carbonitrile* (**SF9c**): yellow oil (104.3 mg, yield 79%). ^1^H-NMR (300 MHz, CDCl_3_) δ 1.66 (s, 3H), 2.54–2.32 (m, 3H), 2.73–2.59 (m, 1H), 3.49–3.33 (m, 1H), 4.02–3.88 (m, 1H), 4.26 (dd, *J* = 11.7, 4.8 Hz, 1H), 4.56 (dd, *J* = 13.7, 5.2 Hz, 1H), 6.52 (s, 1H), 7.26–7.19 (m, 1H), 7.52–7.44 (m, 2H); ^13^C-NMR (125 MHz, CDCl_3_) δ 173.1 (CO), 144.6 (C, Ar), 135.4 (C, Ar), 129.7 (C, Ar), 125.7 (CH, Ar), 121.2 (CH, Ar), 118.7 (CN), 113.9 (CH, Ar), 102.8 (C, Ar), 95.6 (CH, Ar), 59.3 (C), 41.5 (CH_2_), 34.8 (CH_2_), 33.9 (CH_2_), 30.2 (CH_2_), 27.6 (CH_3_); ESI-LRMS *m*/*z*: 266 [M + H]^+^; ESI-HRMS *m*/*z* calcd for M + H^+^ 266.1288, found: 266.1282. The characterization data is in accordance with that reported in [45].

*9-Fluoro-12b-methyl-1,5,6,12b-tetrahydropyrrolo[2’,1’:3,4]pyrazino[1,2-a]indol-3(2H)-one* (**SF9d**): pale yellow solid (43.6 mg, yield 68%), mp 71–72 °C. ^1^H-NMR (500 MHz, DMSO-*d*_6_) δ 1.58 (s, 3H), 2.30–2.18 (m, 2H), 2.44–2.36 (m, 1H), 2.63–2.52 (m, 1H), 3.49–3.38 (m, 1H), 3.77–3.67 (m, 1H), 4.32–4.22 (m, 2H), 6.36 (s, 1H), 6.94–6.86 (m, 1H), 7.26 (dd, *J* = 10.2, 2.2 Hz, 1H), 7.50 (dd, *J* = 8.6, 5.4 Hz, 1H); ^13^C-NMR (125 MHz, DMSO-*d*_6_) δ 172.1 (CO), 158.5 (d, *J_C–F_* = 234.3 Hz, CF, Ar), 142.9 (d, *J_C–F_* = 3.7 Hz, C, Ar), 135.2 (d, *J_C–F_* = 12.5 Hz, C, Ar), 124.2 (C, Ar), 120.9 (d, *J_C–F_* = 10.0 Hz, CH, Ar), 108.1 (d, *J_C–F_* = 24.3 Hz, CH, Ar), 96.3 (d, *J_C–F_* = 26.3 Hz, CH, Ar), 95.6 (CH, Ar), 58.6 (C), 41.1 (CH_2_), 34.3 (CH_2_), 33.5 (CH_2_), 29.6 (CH_2_), 27.3 (CH_3_); ESI-LRMS *m*/*z*: 259 [M + H]^+^; ESI-HRMS *m*/*z* calcd for M + H^+^ 259.1241, found: 259.1238.

*10-Chloro-12b-methyl-1,5,6,12b-tetrahydropyrrolo[2’,1’:3,4]pyrazino[1,2-a]indol-3(2H)-one* (**SF9e**): pale yellow oil (87.5 mg, yield 64%). ^1^H-NMR (300 MHz, CDCl_3_) δ 1.63 (s, 3H), 2.52–2.32 (m, 3H), 2.71–2.56 (m, 1H), 3.45–3.32 (m, 1H), 3.94–3.81 (m, 1H), 4.17 (dd, *J* = 11.6, 4.6 Hz, 1H), 4.52 (dd, *J* = 13.7, 5.0 Hz, 1H), 6.23 (s, 1H), 7.20–7.11 (m, 2H), 7.52 (s, 1H); ^13^C-NMR (100 MHz, CDCl_3_) δ 173.1 (CO), 143.0 (C, Ar), 134.1 (C, Ar), 129.1 (C, Ar), 126.1 (C, Ar), 121.8 (CH, Ar), 119.9 (CH, Ar), 110.1 (CH, Ar), 96.0 (CH, Ar), 59.4 (C), 41.3 (CH_2_), 34.8 (CH_2_), 34.0 (CH_2_), 30.2 (CH_2_), 27.7 (CH_3_); ESI-LRMS *m*/*z*: 277 ([M + H]^+^, Cl^37^), 275 ([M + H]^+^, Cl^35^); ESI-HRMS *m*/*z* calcd for M + H^+^ 275.0946, found: 275.0943. The characterization data is in accordance with that reported in [45].

*13b-Methyl-2,3,6,7-tetrahydro-1H-pyrido[2’,1’:3,4]pyrazino[1,2-a]indol-4(13bH)-one* (**SF10a**): white solid (73.5 mg, yield 58%), mp 113–114 °C. ^1^H-NMR (500 MHz, DMSO-*d*_6_) δ 1.67 (s, 3H), 1.78–1.69 (m, 1H), 1.98–1.88 (m, 1H), 2.07–1.99 (m, 1H), 2.42–2.26 (m, 3H), 3.40–3.25 (m, 1H), 3.81–3.70 (m, 1H), 4.33–4.24 (m, 1H), 4.95–4.85 (m, 1H), 6.34 (s, 1H), 7.06–7.01 (m, 1H), 7.14–7.07 (m, 1H), 7.37 (d, *J* = 8.1 Hz, 1H), 7.50 (d, *J* = 7.8 Hz, 1H); ^13^C-NMR (125 MHz, DMSO-*d*_6_) δ 168.1 (CO), 142.4 (C, Ar), 135.1 (C, Ar), 127.7 (C, Ar), 120.6 (CH, Ar), 119.8 (CH, Ar), 119.7 (CH, Ar), 109.4 (CH, Ar), 95.2 (CH, Ar), 56.7 (C), 41.2 (CH_2_), 36.6 (CH_2_), 34.5 (CH_2_), 31.7 (CH_2_), 28.2 (CH_3_), 16.9 (CH_2_); ESI-LRMS *m*/*z*: 255 [M + H]^+^; ESI-HRMS *m*/*z* calcd for M + H^+^ 255.1492, found: 255.1488. The characterization data is in accordance with that reported in [43].

*12,13b-Dimethyl-2,3,6,7-tetrahydro-1H-pyrido[2’,1’:3,4]pyrazino[1,2-a]indol-4(13bH)-one* (**SF10b**): colorless oil (95.4 mg, yield 71%). ^1^H-NMR (300 MHz, CDCl_3_) δ 1.73 (s, 3H), 2.06–1.86 (m, 2H), 2.26–2.11 (m, 1H), 2.50–2.32 (m, 2H), 2.65–2.51 (m, 4H), 3.39–3.23 (m, 1H), 4.01–3.87 (m, 1H), 4.22–4.10 (m, 1H), 5.16 (dd, *J* = 13.7, 4.5 Hz, 1H), 6.26 (s, 1H), 6.99–6.88 (m, 1H), 7.18–7.07 (m, 2H); ^13^C-NMR (100 MHz, CDCl_3_) δ 169.5 (CO), 141.4 (C, Ar), 135.2 (C, Ar), 129.9 (C, Ar), 127.9 (C, Ar), 121.6 (CH, Ar), 120.5 (CH, Ar), 106.6 (CH, Ar), 94.3 (CH, Ar), 57.4 (C), 41.7 (CH_2_), 37.5 (CH_2_), 35.3 (CH_2_), 32.2 (CH_2_), 28.9 (CH_3_), 18.8 (CH_3_), 17.4 (CH_2_); ESI-LRMS *m*/*z*: 269 [M + H]^+^; ESI-HRMS *m*/*z* calcd for M + H^+^ 269.1648, found: 269.1645. The characterization data is in accordance with that reported in [45].

*11-Chloro-13b-methyl-2,3,6,7-tetrahydro-1H-pyrido[2’,1’:3,4]pyrazino[1,2-a]indol-4(13bH)-one* (**SF10c**): pale yellow oil (75.5 mg, yield 52%). ^1^H-NMR (300 MHz, CDCl_3_) δ 1.70 (s, 3H), 2.20–1.83 (m, 3H), 2.63–2.27 (m, 3H), 3.37–3.22 (m, 1H), 3.98–3.86 (m, 1H), 4.14 (dd, *J* = 11.6, 4.2 Hz, 1H), 5.16 (dd, *J* = 13.8, 4.2 Hz, 1H), 6.18 (s, 1H), 7.22–7.09 (m, 2H), 7.55–7.48 (m, 1H); ^13^C-NMR (100 MHz, CDCl_3_) δ 169.4 (CO), 143.4 (C, Ar), 134.0 (C, Ar), 129.1 (C, Ar), 126.0 (C, Ar), 121.6 (CH, Ar), 119.8 (CH, Ar), 110.0 (CH, Ar), 95.5 (CH, Ar), 57.3 (C), 41.7 (CH_2_), 37.4 (CH_2_), 35.1 (CH_2_), 32.1 (CH_2_), 28.7 (CH_3_), 17.3 (CH_2_); ESI-LRMS *m*/*z*: 291 ([M + H]^+^, Cl^37^), 289 ([M + H]^+^, Cl^35^); ESI-HRMS *m*/*z* calcd for M + H^+^ 289.1102, found: 289.1099. The characterization data is in accordance with that reported in [45].

*13b-Methyl-6,7-dihydroisoindolo[1’,2’:3,4]pyrazino[1,2-a]indol-9(13bH)-one* (**SF11a**): pale yellow solid (95.6 mg, yield 66%), mp 149–150 °C. ^1^H-NMR (400 MHz, DMSO-*d*_6_) δ 1.91 (s, 3H), 3.90–3.75 (m, 2H), 4.45–4.31 (m, 1H), 4.68–4.57 (m, 1H), 6.84 (s, 1H), 7.08–7.00 (m, 1H), 7.17–7.09 (m, 1H), 7.38 (d, *J* = 8.1 Hz, 1H), 7.59–7.48 (m, 2H), 7.80–7.69 (m, 2H), 8.24 (d, *J* = 7.6 Hz, 1H); ^13^C-NMR (100 MHz, DMSO-*d*_6_) δ 166.6 (CO), 150.3 (C, Ar), 137.8 (C, Ar), 135.2 (C, Ar), 132.8 (CH, Ar), 129.9 (C, Ar), 128.7 (CH, Ar), 127.3 (C, Ar), 123.1 (2 × CH, Ar), 121.2 (CH, Ar), 120.0 (2 × CH, Ar), 109.9 (CH, Ar), 97.8 (CH, Ar), 61.4 (C), 41.1 (CH_2_), 34.2 (CH_2_), 28.2 (CH_3_); ESI-LRMS *m*/*z*: 289 [M + H]^+^; ESI-HRMS *m*/*z* calcd for M + H^+^ 289.1335, found: 289.1332. The characterization data is in accordance with that reported in [43].

*15b-Methyl-8,9-dihydro-5H-indolo[2’,1’:3,4]pyrazino[2,1-a]isoquinolin-6(15bH)-one* (**SF12a**): yellow solid (77.6 mg, yield 51%), mp 235–236 °C. ^1^H-NMR (400 MHz, DMSO-*d*_6_) δ 1.89 (s, 3H), 3.20–3.08 (m, 1H), 3.68 (d, *J* = 19.5 Hz, 1H), 3.81–3.72 (m, 1H), 4.10 (d, *J* = 19.4 Hz, 1H), 4.40–4.31 (m, 1H), 4.99–4.90 (m, 1H), 6.83 (s, 1H), 7.15–7.08 (m, 1H), 7.23–7.15 (m, 2H), 7.33–7.25 (m, 2H), 7.44–7.38 (m, 2H), 7.67 (d, *J* = 7.7 Hz, 1H); ^13^C-NMR (125 MHz, DMSO-*d*_6_) δ 168.3 (CO), 138.6 (C, Ar), 136.9 (C, Ar), 136.5 (C, Ar), 132.1 (C, Ar), 128.0 (CH, Ar), 127.6 (CH, Ar), 127.2 (C, Ar), 126.5 (CH, Ar), 124.2 (CH, Ar), 121.4 (CH, Ar), 120.3 (CH, Ar), 119.9 (CH, Ar), 109.7 (CH, Ar), 101.4 (CH, Ar), 61.8 (C), 42.8 (CH_2_), 37.7 (CH_2_), 37.4 (CH_2_), 28.6 (CH_3_); ESI-LRMS *m*/*z*: 303 [M + H]^+^; ESI-HRMS *m*/*z* calcd for M + H^+^ 303.1492, found: 303.1487. The characterization data is in accordance with that reported in [43].

*13b-Methyl-5,6,7,13b-tetrahydro-1H-pyrrolo[2’,1’:3,4][1,4]diazepino[1,2-a]indol-3(2H)-one* (**SF13a**): colorless oil (91.6 mg, yield 72%). ^1^H-NMR (400 MHz, CDCl_3_) δ 1.72 (s, 3H), 2.14–1.88 (m, 3H), 2.53–2.42 (m, 2H), 2.80–2.70 (m, 1H), 3.17–3.04 (m, 1H), 4.20 (ddd, *J* = 14.8, 9.1, 2.5 Hz, 1H), 4.56–4.33 (m, 2H), 6.45 (s, 1H), 7.13–7.06 (m, 1H), 7.24–7.19 (m, 1H), 7.30 (d, *J* = 8.3 Hz, 1H), 7.57 (d, *J* = 7.8 Hz, 1H); ^13^C-NMR (100 MHz, CDCl_3_) δ 174.2 (CO), 142.5 (C, Ar), 137.7 (C, Ar), 126.9 (C, Ar), 122.0 (CH, Ar), 120.7 (CH, Ar), 119.9 (CH, Ar), 109.0 (CH, Ar), 99.6 (CH, Ar), 62.4 (C), 43.4 (CH_2_), 38.5 (CH_2_), 35.0 (CH_2_), 30.5 (CH_2_), 28.0 (CH_2_), 26.4 (CH_3_); ESI-LRMS *m*/*z*: 255 [M + H]^+^; ESI-HRMS *m*/*z* calcd for M + H^+^ 255.1492, found: 255.1489. The characterization data is in accordance with that reported in [45].

*11-Bromo-13b-methyl-5,6,7,13b-tetrahydro-1H-pyrrolo[2’,1’:3,4][1,4]diazepino[1,2-a]indol-3(2H)-one* (**SF13b**): pale yellow oil (89.3 mg, yield 54%). ^1^H-NMR (300 MHz, CDCl_3_) δ 1.71 (s, 3H), 2.15–1.91 (m, 3H), 2.51–2.40 (m, 2H), 2.77–2.66 (m, 1H), 3.16–3.03 (m, 1H), 4.18 (ddd, *J* = 14.8, 8.8, 2.7 Hz, 1H), 4.46–4.32 (m, 2H), 6.37 (s, 1H), 7.14 (d, *J* = 8.8 Hz, 1H), 7.30–7.26 (m, 1H), 7.66 (d, *J* = 1.9 Hz, 1H); ^13^C-NMR (100 MHz, CDCl_3_) δ 174.1 (CO), 143.7 (C, Ar), 136.4 (C, Ar), 128.5 (C, Ar), 124.8 (CH, Ar), 123.1 (CH, Ar), 112.9 (C, Ar), 110.6 (CH, Ar), 99.1 (CH, Ar), 62.3 (C), 43.7 (CH_2_), 38.5 (CH_2_), 34.9 (CH_2_), 30.4 (CH_2_), 27.9 (CH_2_), 26.3 (CH_3_); ESI-LRMS *m*/*z*: 335 ([M + H]^+^, Br^81^), 333 ([M + H]^+^, Br^79^); ESI-HRMS *m*/*z* calcd for M + H^+^ 333.0597, found: 333.0595. The characterization data is in accordance with that reported in [45].

*12,13b-Dimethyl-5,6,7,13b-tetrahydro-1H-pyrrolo[2’,1’:3,4][1,4]diazepino[1,2-a]indol-3(2H)-one* (**SF13c**): colorless oil (107.1 mg, yield 80%). ^1^H-NMR (300 MHz, CDCl_3_) δ 1.73 (s, 3H), 2.03–1.82 (m, 2H), 2.17–2.07 (m, 1H), 2.54–2.44 (m, 2H), 2.55 (s, 3H), 2.85–2.70 (m, 1H), 3.17–3.04 (m, 1H), 4.25–4.13 (m, 1H), 4.52–4.33 (m, 2H), 6.46 (s, 1H), 6.95–6.88 (m, 1H), 7.18–7.12 (m, 2H); ^13^C-NMR (100 MHz, CDCl_3_) δ 174.0 (CO), 141.8 (C, Ar), 137.3 (C, Ar), 130.1 (C, Ar), 126.5 (C, Ar), 122.1 (CH, Ar), 120.0 (CH, Ar), 106.6 (CH, Ar), 97.9 (CH, Ar), 62.3 (C), 43.5 (CH_2_), 38.4 (CH_2_), 34.8 (CH_2_), 30.4 (CH_2_), 28.0 (CH_2_), 26.3 (CH_3_), 18.6 (CH_3_); ESI-LRMS *m*/*z*: 269 [M + H]^+^; ESI-HRMS *m*/*z* calcd for M + H^+^ 269.1648, found: 269.1644. The characterization data is in accordance with that reported in [45].

*14b-Methyl-1,14b-dihydroindolo[1,2-a]pyrrolo[2,1-c]quinoxalin-3(2H)-one* (**SF14a**): pale yellow solid (123.5 mg, yield 86%), mp 179–180 °C. ^1^H-NMR (400 MHz, DMSO-*d*_6_) δ 1.39 (s, 3H), 2.69–2.45 (m, 3H), 2.95–2.81 (m, 1H), 6.64 (s, 1H), 7.25–7.17 (m, 1H), 7.35–7.26 (m, 2H), 7.46–7.39 (m, 1H), 7.67 (d, *J* = 7.7 Hz, 1H), 8.13–8.04 (m, 2H), 8.16 (d, *J* = 8.1 Hz, 1H); ^13^C-NMR (100 MHz, DMSO-*d*_6_) δ 172.2 (CO), 142.7 (C, Ar), 133.1 (C, Ar), 129.3 (C, Ar), 128.8 (C, Ar), 125.8 (CH, Ar), 125.7 (C, Ar), 124.1 (CH, Ar), 123.0 (CH, Ar), 122.7 (CH, Ar), 121.4 (CH, Ar), 121.2 (CH, Ar), 117.2 (CH, Ar), 111.8 (CH, Ar), 97.6 (CH, Ar), 59.4 (C), 31.2 (CH_2_), 30.0 (CH_2_), 25.9 (CH_3_); ESI-LRMS *m*/*z*: 289 [M + H]^+^; ESI-HRMS *m*/*z* calcd for M + H^+^ 289.1335, found: 289.1331. The characterization data is in accordance with that reported in [43].

*2-Hexyl-14b-methyl-1,14b-dihydroindolo[1,2-a]pyrrolo[2,1-c]quinoxalin-3(2H)-one* (**SF14b**): pale yellow oil (159.8 mg, yield 86% (dr = 1.5:1)), and the two diastereomers were inseparable by chromatography. ^1^H-NMR (600 MHz, CDCl_3_) δ 1.00–0.80 (m, 3.02H), 1.53–1.22 (m, 11.93H), 2.09–1.96 (m, 1.03H), 2.14–2.08 (m, 0.34H), 2.35–2.27 (m, 0.53H), 2.62–2.52 (m, 0.31H), 2.81–2.73 (m, 0.53H), 2.91–2.82 (m, 0.53H), 3.03–2.94 (m, 0.32H), 6.44 (s, 0.51H), 6.47 (s, 0.34H), 7.25–7.15 (m, 1.61H), 7.36–7.27 (m, 1.91H), 7.43–7.37 (m, 0.41H), 7.68–7.61 (m, 0.92H), 7.86 (dd, *J* = 7.9, 1.3 Hz, 0.34H), 8.06–7.95 (m, 1.91H), 8.28 (dd, *J* = 8.1, 1.4 Hz, 0.53H); ^13^C-NMR (150 MHz, CDCl_3_) δ 175.3 (CO), 174.1 (CO), 143.4 (C, Ar), 141.9 (C, Ar), 134.4 (C, Ar), 133.6 (C, Ar), 130.6 (C, Ar), 129.8 (C, Ar), 129.5 (C, Ar), 129.4 (C, Ar), 126.4 (CH, Ar), 126.2 (C, Ar), 125.6 (CH, Ar), 125.4 (CH, Ar), 124.3 (2 × CH, Ar), 123.1 (2 × CH, Ar), 122.8 (CH, Ar), 121.6 (CH, Ar), 121.3 (2 × CH, Ar), 117.2 (CH, Ar), 117.1 (CH, Ar), 111.9 (CH, Ar), 111.7 (CH, Ar), 97.4 (CH, Ar), 97.1 (CH, Ar), 58.3 (C), 41.9 (CH), 41.5 (CH), 39.5 (CH_2_), 37.7 (CH_2_), 32.1 (CH_2_), 31.9 (CH_2_), 31.8 (CH_2_), 30.9 (CH_2_), 29.3 (CH_2_), 29.3 (CH_2_), 28.9 (CH_3_), 27.6 (CH_2_), 27.2 (CH_2_), 26.2 (CH_3_), 22.8 (CH_2_), 22.7 (CH_2_), 14.2 (2 × CH_3_); ESI-LRMS *m*/*z*: 373 [M + H]^+^; ESI-HRMS *m*/*z* calcd for M + H^+^ 373.2274, found: 373.2273.

*15b-Methyl-2,3-dihydro-1H-indolo[1,2-a]pyrido[2,1-c]quinoxalin-4(15bH)-one* (**SF15**): pale yellow oil (89.4 mg, yield 59%). ^1^H-NMR (400 MHz, DMSO-*d*_6_) δ 1.29 (s, 3H), 1.97–1.75 (m, 2H), 2.42–2.28 (m, 2H), 2.71–2.56 (m, 2H), 6.60 (s, 1H), 7.23–7.17 (m, 1H), 7.33–7.24 (m, 2H), 7.47–7.40 (m, 1H), 7.71–7.64 (m, 2H), 8.08–8.01 (m, 2H); ^13^C-NMR (100 MHz, DMSO-*d*_6_) δ 168.2 (CO), 142.5 (C, Ar), 133.2 (C, Ar), 130.5 (C, Ar), 129.1 (C, Ar), 128.4 (CH, Ar), 127.8 (C, Ar), 126.6 (CH, Ar), 123.4 (CH, Ar), 122.8 (CH, Ar), 121.2 (2 × CH, Ar), 116.9 (CH, Ar), 111.5 (CH, Ar), 96.8 (CH, Ar), 57.3 (C), 33.3 (CH_2_), 32.9 (CH_2_), 27.7 (CH_3_), 17.1 (CH_2_); ESI-LRMS *m*/*z*: 303 [M + H]^+^; ESI-HRMS *m*/*z* calcd for M + H^+^ 303.1492, found: 303.1486. The characterization data is in accordance with that reported in [43].

*15b-Mmethylindolo[1,2-a]isoindolo[1,2-c]quinoxalin-11(15bH)-one* (**SF16**): white solid (100.5 mg, yield 60%), mp 149–150 °C. ^1^H-NMR (400 MHz, DMSO-*d*_6_) δ 1.65 (s, 3H), 6.77 (s, 1H), 7.24–7.18 (m, 1H), 7.35–7.29 (m, 1H), 7.47–7.40 (m, 1H), 7.57–7.51 (m, 1H), 7.65 (d, *J* = 7.5 Hz, 1H), 7.76–7.68 (m, 1H), 7.96–7.87 (m, 2H), 8.04 (dd, *J* = 7.9, 1.5 Hz, 1H), 8.15 (d, *J* = 8.4 Hz, 1H), 8.31–8.26 (m, 1H), 8.35 (d, *J* = 7.6 Hz, 1H); ^13^C-NMR (100 MHz, DMSO-*d*_6_) δ 164.1 (CO), 146.4 (C, Ar), 137.1 (C, Ar), 133.9 (C, Ar), 133.5 (CH, Ar), 129.7 (C, Ar), 129.5 (CH, Ar), 129.3 (C, Ar), 128.9 (C, Ar), 126.5 (CH, Ar), 124.6 (C, Ar), 124.4 (CH, Ar), 124.0 (CH, Ar), 123.9 (CH, Ar), 123.7 (CH, Ar), 123.5 (CH, Ar), 121.6 (CH, Ar), 121.3 (CH, Ar), 117.5 (CH, Ar), 111.9 (CH, Ar), 99.3 (CH, Ar), 61.2 (C), 26.3 (CH_3_); ESI-LRMS *m*/*z*: 337 [M + H]^+^; ESI-HRMS *m*/*z* calcd for M + H^+^ 337.1335, found: 337.1329. The characterization data is in accordance with that reported in [43].

*11b-Methyl-4,5-dihydro-3H-pyrrolo[3’,2’:3,4]pyrido[2,1-a]isoindol-7(11bH)-one* (**SF17**): pale yellow solid (77.1 mg, yield 65%), mp 237–238 °C. ^1^H-NMR (500 MHz, DMSO-*d*_6_) δ 1.66 (s, 3H), 2.66–2.54 (m, 2H), 3.41–3.27 (m, 1H), 4.48–4.37 (m, 1H), 6.31–6.26 (m, 1H), 6.62–6.55 (m, 1H), 7.49–7.42 (m, 1H), 7.68–7.60 (m, 2H), 7.97 (d, *J* = 7.6 Hz, 1H), 10.56 (s, 1H); ^13^C-NMR (100 MHz, DMSO-*d*_6_) δ 166.8 (CO), 152.2 (C, Ar), 131.9 (CH, Ar), 130.0 (C, Ar), 127.8 (CH, Ar), 122.7 (CH, Ar), 122.7 (C, Ar), 122.3 (CH, Ar), 119.9 (C, Ar), 116.8 (CH, Ar), 104.0 (CH, Ar), 62.3 (C), 34.5 (CH_2_), 27.7 (CH_3_), 22.6 (CH_2_); ESI-LRMS *m*/*z*: 239 [M + H]^+^; ESI-HRMS *m*/*z* calcd for M + H^+^ 239.1179, found: 239.1175. The characterization data is in accordance with that reported in [43].

*12b-Methyl-5,6-dihydropyrrolo[2’,1’:3,4]pyrazino[2,1-a]isoindol-8(12bH)-one* (**SF18**): pale yellow solid (110.9 mg, yield 93%), mp 156–157 °C. ^1^H-NMR (500 MHz, DMSO-*d*_6_) δ 1.76 (s, 3H), 3.69–3.60 (m, 1H), 3.78–3.70 (m, 1H), 4.08 (dd, *J* = 12.0, 3.6 Hz, 1H), 4.45 (dd, *J* = 13.3, 4.2 Hz, 1H), 6.06–5.98 (m, 1H), 6.39–6.31 (m, 1H), 6.67–6.60 (m, 1H), 7.55–7.47 (m, 1H), 7.75–7.65 (m, 2H), 8.06 (d, *J* = 7.9 Hz, 1H); ^13^C-NMR (100 MHz, DMSO-*d*_6_) δ 166.8 (CO), 151.1 (C, Ar), 132.6 (CH, Ar), 130.0 (C, Ar), 129.6 (C, Ar), 128.4 (CH, Ar), 122.9 (CH, Ar), 122.8 (CH, Ar), 119.4 (CH, Ar), 107.8 (CH, Ar), 104.2 (CH, Ar), 61.3 (C), 43.8 (CH_2_), 34.9 (CH_2_), 28.5 (CH_3_); ESI-LRMS *m*/*z*: 239 [M + H]^+^; ESI-HRMS *m*/*z* calcd for M + H^+^ 239.1179, found: 239.1175. The characterization data is in accordance with that reported in [43].

*9a-Methyl-4,5,9,9a-tetrahydrothieno[2,3-g]indolizin-7(8H)-one* (**SF19**): pale yellow solid (65.1 mg, yield 63%), mp 128–129 °C. ^1^H-NMR (400 MHz, DMSO-*d*_6_) δ 1.42 (s, 3H), 1.90–1.79 (m, 1H), 2.31–2.15 (m, 2H), 2.61–2.52 (m, 1H), 2.74–2.62 (m, 1H), 2.85–2.76 (m, 1H), 3.09–2.98 (m, 1H), 4.15 (dd, *J* = 13.2, 5.6 Hz, 1H), 6.99 (d, *J* = 5.2 Hz, 1H), 7.37 (d, *J* = 5.1 Hz, 1H); ^13^C-NMR (100 MHz, DMSO-*d*_6_) δ 171.6 (CO), 141.8 (C, Ar), 131.3 (C, Ar), 124.2 (CH, Ar), 124.1 (CH, Ar), 60.4 (C), 33.7 (CH_2_), 33.4 (CH_2_), 30.1 (CH_2_), 25.8 (CH_3_), 24.3 (CH_2_); ESI-LRMS *m*/*z*: 208 [M + H]^+^; ESI-HRMS *m*/*z* calcd for M + H^+^ 208.0791, found: 208.0788. The characterization data is in accordance with that reported in [43].

*11b-Methyl-4,5-dihydrothieno[3’,2’:3,4]pyrido[2,1-a]isoindol-7(11bH)-one* (**SF20**): yellow solid (105.6 mg, yield 83%), mp 199–200 °C. ^1^H-NMR (400 MHz, DMSO-*d*_6_) δ 1.75 (s, 3H), 2.82–2.71 (m, 1H), 2.93–2.84 (m, 1H), 3.46–3.38 (m, 1H), 4.47 (dd, *J* = 13.4, 5.7 Hz, 1H), 7.39 (d, *J* = 5.3 Hz, 1H), 7.56–7.48 (m, 2H), 7.74–7.63 (m, 2H), 8.16 (d, *J* = 7.6 Hz, 1H); ^13^C-NMR (100 MHz, DMSO-*d*_6_) δ 166.8 (CO), 150.2 (C, Ar), 137.8 (C, Ar), 132.8 (C, Ar), 132.3 (CH, Ar), 130.1 (C, Ar), 128.4 (CH, Ar), 125.3 (CH, Ar), 124.0 (CH, Ar), 123.0 (2 × CH, Ar), 63.4 (C), 34.7 (CH_2_), 27.0 (CH_3_), 24.8 (CH_2_); ESI-LRMS *m*/*z*: 256 [M + H]^+^; ESI-HRMS *m*/*z* calcd for M + H^+^ 256.0791, found: 256.0785. The characterization data is in accordance with that reported in [43].

*9a-Methyl-4,5,9,9a-tetrahydrothieno[3,2-g]indolizin-7(8H)-one* (**SF21**): colorless oil (93.3 mg, yield 90%). ^1^H-NMR (500 MHz, DMSO-*d*_6_) δ 1.50 (s, 3H), 2.03–1.92 (m, 1H), 2.32–2.19 (m, 2H), 2.61–2.51 (m, 2H), 2.72–2.63 (m, 1H), 3.07–2.96 (m, 1H), 4.11 (dd, *J* = 13.3, 6.2 Hz, 1H), 6.80 (d, *J* = 5.1 Hz, 1H), 7.40 (d, *J* = 5.0 Hz, 1H); ^13^C-NMR (100 MHz, DMSO-*d*_6_) δ 171.7 (CO), 141.8 (C, Ar), 132.3 (C, Ar), 127.1 (CH, Ar), 123.5 (CH, Ar), 60.4 (C), 35.1 (CH_2_), 33.6 (CH_2_), 30.2 (CH_2_), 27.9 (CH_3_), 25.1 (CH_2_); ESI-LRMS *m*/*z*: 208 [M + H]^+^; ESI-HRMS *m*/*z* calcd for M + H^+^ 208.0791, found: 208.0788. The characterization data is in accordance with that reported in [43].

*11b-Methyl-4,5-dihydrothieno[2’,3’:3,4]pyrido[2,1-a]isoindol-7(11bH)-one* (**SF22**): pale yellow solid (108.6 mg, yield 85%), mp 137–138 °C. ^1^H-NMR (500 MHz, DMSO-*d*_6_) δ 1.82 (s, 3H), 2.62 (ddd, *J* = 16.2, 11.7, 6.5 Hz, 1H), 2.76 (dd, *J* = 16.2, 4.5 Hz, 1H), 3.40 (ddd, *J* = 13.4, 12.0, 4.7 Hz, 1H), 4.43 (dd, *J* = 13.5, 6.2 Hz, 1H), 6.82 (d, *J* = 5.1 Hz, 1H), 7.45 (d, *J* = 5.1 Hz, 1H), 7.57–7.49 (m, 1H), 7.76–7.67 (m, 2H), 7.97–7.90 (m, 1H); ^13^C-NMR (100 MHz, DMSO-*d*_6_) δ 166.5 (CO), 150.2 (C, Ar), 138.0 (C, Ar), 133.4 (C, Ar), 132.5 (CH, Ar), 130.0 (C, Ar), 128.7 (CH, Ar), 127.1 (CH, Ar), 124.4 (CH, Ar), 123.1 (CH, Ar), 122.4 (CH, Ar), 63.1 (C), 34.3 (CH_2_), 28.8 (CH_3_), 25.6 (CH_2_); ESI-LRMS *m*/*z*: 256 [M + H]^+^; ESI-HRMS *m*/*z* calcd for M + H^+^ 256.0791, found: 256.0789. The characterization data is in accordance with that reported in [43].

*12b-Methyl-8,12b-dihydro-4H-thieno[2’,3’:3,4]pyrido[2,1-a]isoquinolin-7(5H)-one* (**SF23**): pale yellow solid (63.4 mg, yield 47%), mp 155–156 °C. ^1^H-NMR (500 MHz, DMSO-*d*_6_) δ 1.87 (s, 3H), 2.50–2.44 (m, 1H), 2.76–2.68 (m, 1H), 3.10–2.99 (m, 1H), 3.64 (d, *J* = 20.3 Hz, 1H), 3.97 (d, *J* = 20.2 Hz, 1H), 4.90–4.79 (m, 1H), 6.91 (d, *J* = 5.1 Hz, 1H), 7.32–7.22 (m, 3H), 7.55 (d, *J* = 5.1 Hz, 1H), 7.77–7.71 (m, 1H); ^13^C-NMR (100 MHz, DMSO-*d*_6_) δ 167.9 (CO), 139.0 (C, Ar), 138.8 (C, Ar), 136.4 (C, Ar), 131.3 (C, Ar), 128.0 (CH, Ar), 127.6 (CH, Ar), 127.4 (CH, Ar), 126.5 (CH, Ar), 124.8 (CH, Ar), 124.5 (CH, Ar), 62.6 (C), 37.0 (CH_2_), 36.7 (CH_2_), 31.7 (CH_3_), 25.2 (CH_2_); ESI-LRMS *m*/*z*: 270 [M + H]^+^; ESI-HRMS *m*/*z* calcd for M + H^+^ 270.0947, found: 270.0942. The characterization data is in accordance with that reported in [43].

*8,9-Dimethoxy-10b-methyl-1,5,6,10b-tetrahydropyrrolo[2,1-a]isoquinolin-3(2H)-one* (**SF24a**): white solid (114.5 mg, yield 88%), mp 53–54 °C. ^1^H-NMR (500 MHz, DMSO-*d*_6_) δ 1.45 (s, 3H), 1.93–1.82 (m, 1H), 2.25–2.15 (m, 1H), 2.45–2.37 (m, 1H), 2.59–2.47 (m, 1H), 2.70–2.62 (m, 2H), 3.05–2.96 (m, 1H), 3.71 (s, 3H), 3.75 (s, 3H), 4.08–4.00 (m, 1H), 6.66 (s, 1H), 6.78 (s, 1H); ^13^C-NMR (100 MHz, DMSO-*d*_6_) δ 171.3 (CO), 147.7 (C, Ar), 147.4 (C, Ar), 134.9 (C, Ar), 124.1 (C, Ar), 111.9 (CH, Ar), 108.7 (CH, Ar), 60.3 (C), 55.8 (OCH_3_), 55.5 (OCH_3_), 34.3 (CH_2_), 33.4 (CH_2_), 30.2 (CH_2_), 27.7 (CH_2_), 26.8 (CH_3_); ESI-LRMS *m*/*z*: 262 [M + H]^+^; ESI-HRMS *m*/*z* calcd for M + H^+^ 262.1438, found: 262.1434. The characterization data is in accordance with that reported in [43].

*2-Hexyl-8,9-dimethoxy-10b-methyl-1,5,6,10b-tetrahydropyrrolo[2,1-a]isoquinolin-3(2H)-one* (**SF24b**): white solid (81.2 mg, yield 47% (dr = 4:1)), mp 65–67 °C, and the two diastereomers were inseparable by chromatography. ^1^H-NMR (400 MHz, DMSO-*d*_6_) δ 0.85–0.72 (m, 3H), 1.37–0.99 (m, 10H), 1.47–1.35 (m, 3H), 1.73–1.57 (m, 1H), 2.43–2.35 (m, 1H), 2.52–2.44 (m, 1H), 2.70–2.52 (m, 2H), 3.12–2.88 (m, 1H), 3.65–3.59 (m, 3H), 3.68 (s, 3H), 4.00–3.89 (m, 1H), 6.61–6.51 (m, 1H), 6.77–6.69 (m, 1H); ^13^C-NMR (100 MHz, DMSO-*d*_6_) δ 174.9 (CO), 147.5 (C, Ar), 147.4 (C, Ar), 134.7 (C, Ar), 124.4 (C, Ar), 112.0 (CH, Ar), 108.8 (CH, Ar), 59.5 (C, Ar), 55.7 (OCH_3_), 55.4 (OCH_3_), 41.2 (CH), 39.5 (CH_2_), 34.2 (CH_2_), 31.8 (CH_2_), 31.2 (CH_2_), 30.8 (CH_3_), 28.6 (CH_2_), 27.0 (CH_2_), 26.9 (CH_2_), 22.0 (CH_2_), 14.0 (CH_3_); ESI-LRMS *m*/*z*: 346 [M + H]^+^; ESI-HRMS *m*/*z* calcd for M + H^+^ 346.2377, found: 346.2375.

*9,10-Dimethoxy-11b-methyl-2,3,6,7-tetrahydro-1H-pyrido[2,1-a]isoquinolin-4(11bH)-one* (**SF25**): white solid (84.4 mg, yield 61%), mp 75–76 °C. ^1^H-NMR (400 MHz, DMSO-*d*_6_) δ 1.55 (s, 3H), 1.64–1.57 (m, 1H), 1.75–1.64 (m, 1H), 1.96–1.81 (m, 1H), 2.37–2.16 (m, 2H), 2.49–2.41 (m, 1H), 2.68–2.53 (m, 2H), 2.87–2.75 (m, 1H), 3.71 (s, 3H), 3.74 (s, 3H), 4.75–4.63 (m, 1H), 6.65 (s, 1H), 6.84 (s, 1H); ^13^C-NMR (100 MHz, DMSO-*d*_6_) δ 167.7 (CO), 147.4 (C, Ar), 147.2 (C, Ar), 135.2 (C, Ar), 125.5 (C, Ar), 111.7 (CH, Ar), 109.3 (CH, Ar), 58.2 (C), 55.8 (OCH_3_), 55.4 (OCH_3_), 36.7 (CH_2_), 34.8 (CH_2_), 31.5 (CH_2_), 28.5 (CH_2_), 27.3 (CH_3_), 16.6 (CH_2_); ESI-LRMS *m*/*z*: 276 [M + H]^+^; ESI-HRMS *m*/*z* calcd for M + H^+^ 276.1594, found: 276.1589. The characterization data is in accordance with that reported in [43].

*2,3-Dimethoxy-12b-methyl-5,6-dihydroisoindolo[1,2-a]isoquinolin-8(12bH)-one* (**SF26**): white solid (118.8 mg, yield 77%), mp 187–188 °C. ^1^H-NMR (500 MHz, DMSO-*d*_6_) δ 1.82 (s, 3H), 2.83–2.67 (m, 2H), 3.42–3.34 (m, 1H), 3.69 (s, 3H), 3.83 (s, 3H), 4.39–4.31 (m, 1H), 6.69 (s, 1H), 7.39 (s, 1H), 7.53–7.47 (m, 1H), 7.72–7.65 (m, 2H), 8.30 (d, *J* = 7.7 Hz, 1H); ^13^C-NMR (125 MHz, DMSO-*d*_6_) δ 166.5 (CO), 151.1 (C, Ar), 147.8 (C, Ar), 147.5 (C, Ar), 132.2 (CH, Ar), 130.8 (C, Ar), 130.3 (C, Ar), 128.3 (CH, Ar), 125.3 (C, Ar), 123.4 (CH, Ar), 122.8 (CH, Ar), 112.2 (CH, Ar), 110.2 (CH, Ar), 63.4 (C), 56.1 (OCH_3_), 55.5 (OCH_3_), 34.6 (CH_2_), 28.7 (CH_2_), 28.2 (CH_3_); ESI-LRMS *m*/*z*: 310 [M + H]^+^; ESI-HRMS *m*/*z* calcd for M + H^+^ 310.1438, found: 310.1432. The characterization data is in accordance with that reported in [43].

*3a-Methyl-2,3,3a,4-tetrahydropyrrolo[1,2-a]quinazoline-1,5-dione* (**SF27a**): white solid (91.2 mg, yield 84%), mp 177–178 °C. ^1^H-NMR (500 MHz, DMSO-*d*_6_) δ 1.42 (s, 3H), 2.30–2.19 (m, 2H), 2.56–2.49 (m, 1H), 2.78–2.68 (m, 1H), 7.32–7.25 (m, 1H), 7.65–7.57 (m, 1H), 7.91 (dd, *J* = 7.7, 1.4 Hz, 1H), 8.07 (d, *J* = 8.0 Hz, 1H), 8.94 (s, 1H); ^13^C-NMR (100 MHz, DMSO-*d*_6_) δ 171.9 (CO), 161.2 (CO), 135.7 (C, Ar), 133.2 (CH, Ar), 127.7 (CH, Ar), 124.5 (CH, Ar), 119.9 (CH, Ar), 119.8 (C, Ar), 74.0 (C), 32.4 (CH_2_), 29.6 (CH_2_), 26.4 (CH_3_); ESI-LRMS *m*/*z*: 217 [M + H]^+^; ESI-HRMS *m*/*z* calcd for M + H^+^ 217.0972, found: 217.0969. The characterization data is in accordance with that reported in [43].

*3a,4-Dimethyl-2,3,3a,4-tetrahydropyrrolo[1,2-a]quinazoline-1,5-dione* (**SF27b**): white solid (97.5 mg, yield 85%), mp 105–107 °C. ^1^H-NMR (600 MHz, CDCl_3_) δ 1.46 (s, 3H), 2.30 (ddd, *J* = 12.0, 6.1, 4.0 Hz, 1H), 2.48–2.39 (m, 1H), 2.70–2.63 (m, 2H), 3.07 (s, 3H), 7.27–7.24 (m, 1H), 7.56–7.51 (m, 1H), 8.08 (dd, *J* = 7.8, 1.6 Hz, 1H), 8.26 (dd, *J* = 8.2, 0.8 Hz, 1H); ^13^C-NMR (150 MHz, CDCl_3_) δ 171.3 (CO), 162.1 (CO), 135.2 (C, Ar), 133.4 (CH, Ar), 128.6 (CH, Ar), 125.0 (CH, Ar), 119.7 (CH, Ar), 119.4 (C, Ar), 78.5 (C), 32.4 (CH_2_), 30.3 (CH_2_), 27.8 (CH_3_), 21.8 (CH_3_); ESI-LRMS *m*/*z*: 231 [M + H]^+^; ESI-HRMS *m*/*z* calcd for M + H^+^ 231.1128, found: 231.1127. The characterization data is in accordance with that reported in [43].

*4a-Methyl-3,4,4a,5-tetrahydro-1H-pyrido[1,2-a]quinazoline-1,6(2H)-dione* (**SF28a**): white solid (70.7 mg, yield 61%), mp 198–199 °C. ^1^H-NMR (500 MHz, DMSO-*d*_6_) δ 1.35 (s, 3H), 1.90–1.74 (m, 2H), 2.09–2.02 (m, 1H), 2.17–2.09 (m, 1H), 2.48–2.39 (m, 1H), 2.62–2.54 (m, 1H), 7.36–7.28 (m, 1H), 7.60–7.52 (m, 1H), 7.66 (d, *J* = 8.0 Hz, 1H), 7.86 (dd, *J* = 7.7, 1.4 Hz, 1H), 8.85 (s, 1H); ^13^C-NMR (100 MHz, DMSO-*d*_6_) δ 168.8 (CO), 162.3 (CO), 138.1 (C, Ar), 131.8 (CH, Ar), 126.5 (CH, Ar), 126.2 (CH, Ar), 125.2 (CH, Ar), 123.7 (C, Ar), 71.1 (C), 34.7 (CH_2_), 33.1 (CH_2_), 28.3 (CH_3_), 16.6 (CH_2_); ESI-LRMS *m*/*z*: 231 [M + H]^+^; ESI-HRMS *m*/*z* calcd for M + H^+^ 231.1128, found: 231.1126. The characterization data is in accordance with that reported in [43].

*4a,5-Dimethyl-3,4,4a,5-tetrahydro-1H-pyrido[1,2-a]quinazoline-1,6(2H)-dione* (**SF28b**): white solid (78.3 mg, yield 64%), mp 138–139 °C. ^1^H-NMR (600 MHz, CDCl_3_) δ 1.39 (s, 3H), 1.98–1.86 (m, 2H), 2.17–2.10 (m, 1H), 2.44–2.36 (m, 1H), 2.63–2.55 (m, 1H), 2.74–2.65 (m, 1H), 3.12 (s, 3H), 7.32–7.28 (m, 1H), 7.54–7.49 (m, 1H), 7.62 (dd, *J* = 8.1, 0.9 Hz, 1H), 8.02 (dd, *J* = 7.8, 1.6 Hz, 1H); ^13^C-NMR (150 MHz, CDCl_3_) δ 169.3 (CO), 163.6 (CO), 137.4 (C, Ar), 132.0 (CH, Ar), 127.8 (CH, Ar), 126.2 (CH, Ar), 126.0 (CH, Ar), 123.9 (C, Ar), 75.5 (C), 34.4 (CH_2_), 33.7 (CH_2_), 27.5 (CH_3_), 24.6 (CH_3_), 16.9 (CH_2_); ESI-LRMS *m*/*z*: 245 [M + H]^+^; ESI-HRMS *m*/*z* calcd for M + H^+^ 245.1285, found: 245.1284. The characterization data is in accordance with that reported in [43].

*6a-Methyl-6,6a-dihydroisoindolo[2,1-a]quinazoline-5,11-dione* (**SF29a**): white solid (99.3 mg, yield 75%), mp 219–220 °C. ^1^H-NMR (400 MHz, DMSO-*d*_6_) δ 1.72 (s, 3H), 7.42–7.36 (m, 1H), 7.70–7.64 (m, 1H), 7.77–7.71 (m, 1H), 7.85–7.78 (m, 1H), 7.89 (d, *J* = 7.5 Hz, 1H), 7.95 (d, *J* = 7.6 Hz, 1H), 8.04–7.98 (m, 2H), 9.48 (s, 1H); ^13^C-NMR (100 MHz, DMSO-*d*_6_) δ 164.4 (CO), 162.5 (CO), 146.0 (C, Ar), 135.2 (C, Ar), 133.7 (CH, Ar), 133.6 (CH, Ar), 130.1 (CH, Ar), 129.4 (C, Ar), 128.0 (CH, Ar), 125.1 (CH, Ar), 124.0 (CH, Ar), 122.9 (CH, Ar), 121.2 (CH, Ar), 119.8 (C, Ar), 74.2 (C), 27.3 (CH_3_); ESI-LRMS *m*/*z*: 265 [M + H]^+^; ESI-HRMS *m*/*z* calcd for M + H^+^ 265.0972, found: 265.0966. The characterization data is in accordance with that reported in [43].

*6,6a-Dimethyl-6,6a-dihydroisoindolo[2,1-a]quinazoline-5,11-dione* (**SF29b**): white solid (97.1 mg, yield 70%), mp 182–184 °C. ^1^H-NMR (600 MHz, CDCl_3_) δ 1.72 (s, 3H), 3.25 (s, 3H), 7.37–7.32 (m, 1H), 7.67–7.63 (m, 2H), 7.76–7.70 (m, 2H), 8.05–7.99 (m, 2H), 8.15 (dd, *J* = 8.1, 1.0 Hz, 1H); ^13^C-NMR (150 MHz, CDCl_3_) δ 165.0 (CO), 163.0 (CO), 143.3 (C, Ar), 134.8 (C, Ar), 133.5 (CH, Ar), 132.8 (CH, Ar), 131.4 (C, Ar), 130.5 (CH, Ar), 128.9 (CH, Ar), 125.5 (CH, Ar), 125.2 (CH, Ar), 124.6 (CH, Ar), 121.8 (CH, Ar), 120.6 (C, Ar), 78.1 (C), 29.6 (CH_3_), 23.3 (CH_3_); ESI-LRMS *m*/*z*: 279 [M + H]^+^; ESI-HRMS *m*/*z* calcd for M + H^+^ 279.1128, found: 279.1127. The characterization data is in accordance with that reported in [43].

*4b-Methyl-4bH-isoquinolino[2,1-a]quinazoline-6,12(5H,13H)-dione* (**SF30a**): yellow solid (91.5 mg, yield 66%), mp 225–226 °C. ^1^H-NMR (400 MHz, DMSO-*d*_6_) δ 1.71 (s, 3H), 3.84 (d, *J* = 21.2 Hz, 1H), 4.16 (d, *J* = 21.1 Hz, 1H), 7.30–7.23 (m, 1H), 7.45–7.36 (m, 3H), 7.68–7.61 (m, 1H), 7.79–7.71 (m, 1H), 7.82 (d, *J* = 7.8 Hz, 1H), 7.93 (dd, *J* = 7.7, 1.5 Hz, 1H), 8.89 (s, 1H); ^13^C-NMR (100 MHz, DMSO-*d*_6_) δ 165.5 (CO), 161.9 (CO), 137.6 (C, Ar), 133.1 (C, Ar), 132.1 (CH, Ar), 128.8 (CH, Ar), 128.6 (C, Ar), 127.5 (CH, Ar), 127.2 (CH, Ar), 126.6 (CH, Ar), 126.2 (CH, Ar), 126.1 (CH, Ar), 125.7 (CH, Ar), 123.3 (C, Ar), 73.9 (C), 35.2 (CH_2_), 30.5 (CH_3_); ESI-LRMS *m*/*z*: 279 [M + H]^+^; ESI-HRMS *m*/*z* calcd for M + H^+^ 279.1128, found: 279.1122. The characterization data is in accordance with that reported in [43].

*4b,5-Dimethyl-4bH-isoquinolino[2,1-a]quinazoline-6,12(5H,13H)-dione* (**SF30b**): yellow oil (29.1 mg, yield 20%). ^1^H-NMR (600 MHz, CDCl_3_) δ 1.86 (s, 3H), 2.77 (s, 3H), 3.91 (s, 2H), 7.28 (d, *J* = 7.5 Hz, 1H), 7.45–7.39 (m, 2H), 7.49–7.45 (m, 1H), 7.61–7.55 (m, 2H), 7.63 (d, *J* = 8.0 Hz, 1H), 8.08 (dd, *J* = 7.9, 1.3 Hz, 1H); ^13^C-NMR (150 MHz, DMSO-*d*_6_) δ 169.2 (CO), 162.1 (CO), 138.6 (C, Ar), 131.9 (CH, Ar), 131.8 (C, Ar), 130.6 (C, Ar), 129.9 (CH, Ar), 128.9 (CH, Ar), 128.0 (CH, Ar), 127.7 (CH, Ar), 127.0 (CH, Ar), 126.9 (CH, Ar), 126.7 (CH, Ar), 125.2 (C, Ar), 76.7 (C), 36.2 (CH_2_), 29.9 (CH_3_), 25.7 (CH_3_); ESI-LRMS *m*/*z*: 293 [M + H]^+^; ESI-HRMS *m*/*z* calcd for M + H^+^ 293.1285, found: 293.1284.

*3a-Methyl-2,3,3a,4-tetrahydropyrrolo[2,1-b]quinazolin-1(9H)-one* (**SF31**): white solid (69.4 mg, yield 69%), mp 141–143 °C. ^1^H-NMR (600 MHz, CDCl_3_) δ 1.54 (s, 3H), 2.15–2.05 (m, 2H), 2.52–2.45 (m, 1H), 2.60–2.52 (m, 1H), 4.17 (d, *J* = 16.7 Hz, 1H), 5.02 (d, *J* = 16.8 Hz, 1H), 6.58 (dd, *J* = 8.0, 0.8 Hz, 1H), 6.81–6.76 (m, 1H), 7.07–6.99 (m, 2H); ^13^C-NMR (150 MHz, CDCl_3_) δ 174.3 (CO), 141.9 (C, Ar), 127.6 (CH, Ar), 127.0 (CH, Ar), 119.3 (CH, Ar), 117.4 (C, Ar), 116.5 (CH, Ar), 71.9 (C), 38.6 (CH_2_), 33.0 (CH_2_), 29.6 (CH_2_), 25.6 (CH_3_); ESI-LRMS *m*/*z*: 203 [M + H]^+^; ESI-HRMS *m*/*z* calcd for M+H^+^ 203.1179, found: 203.1178. The characterization data is in accordance with that reported in [43].

*4b-Methyl-4b,5-dihydroisoindolo[1,2-b]quinazolin-12(10H)-one* (**SF32**): pale yellow solid (41.1 mg, yield 33%), mp 222–223 °C. ^1^H-NMR (600 MHz, CDCl_3_) δ 1.71 (s, 3H), 4.24 (s, 1H), 4.45 (d, *J* = 16.9 Hz, 1H), 5.32 (d, *J* = 17.0 Hz, 1H), 6.69 (d, *J* = 8.3 Hz, 1H), 6.90–6.85 (m, 1H), 7.12–7.08 (m, 1H), 7.14 (d, *J* = 7.5 Hz, 1H), 7.56–7.52 (m, 1H), 7.63 (d, *J* = 3.9 Hz, 2H), 7.88 (d, *J* = 7.6 Hz, 1H); ^13^C-NMR (150 MHz, CDCl_3_) δ 165.8 (CO), 147.8 (C, Ar), 140.2 (C, Ar), 132.3 (CH, Ar), 131.5 (C, Ar), 129.6 (CH, Ar), 127.9 (CH, Ar), 127.2 (CH, Ar), 124.4 (CH, Ar), 120.7 (CH, Ar), 120.5 (CH, Ar), 118.7 (C, Ar), 118.1 (CH, Ar), 71.5 (C), 38.0 (CH_2_), 23.9 (CH_3_); ESI-LRMS *m*/*z*: 251 [M + H]^+^; ESI-HRMS *m*/*z* calcd for M + H^+^ 251.1179, found: 251.1178. The characterization data is in accordance with that reported in [43].

*3a-Methyl-2,3,3a,4-tetrahydro-1H-benzo[d]pyrrolo[1,2-a]imidazol-1-one* (**SF33**): colorless oil (45.1 mg, yield 48%). ^1^H-NMR (600 MHz, CDCl_3_) δ 1.51 (s, 3H), 2.44–2.33 (m, 2H), 2.54 (ddd, *J* = 16.8, 8.5, 1.6 Hz, 1H), 2.78 (ddd, *J* = 16.8, 11.7, 8.5 Hz, 1H), 6.68 (dd, *J* = 7.7, 0.7 Hz, 1H), 6.84–6.79 (m, 1H), 6.98–6.93 (m, 1H), 7.43 (dd, *J* = 7.6, 1.1 Hz, 1H); ^13^C-NMR (150 MHz, CDCl_3_) δ 173.9 (CO), 142.8 (C, Ar), 128.7 (C, Ar), 125.4 (CH, Ar), 120.3 (CH, Ar), 115.5 (CH, Ar), 110.7 (CH, Ar), 85.7 (C), 37.8 (CH_2_), 33.7 (CH_2_), 26.3 (CH_3_); ESI-LRMS *m*/*z*: 189 [M + H]^+^; ESI-HRMS *m*/*z* calcd for M + H^+^ 189.1022, found: 189.1023. The characterization data is in accordance with that reported in [34].

*12a-Methyl-12,12a-dihydrobenzo[4,5]imidazo[2,1-a]isoquinolin-6(5H)-one* (**SF34**): colorless oil (26.1 mg, yield 21%). ^1^H-NMR (600 MHz, CDCl_3_) δ 1.69 (s, 3H), 3.71 (d, *J* = 19.0 Hz, 1H), 3.88 (d, *J* = 19.0 Hz, 1H), 4.48 (s, 1H), 6.85 (dd, *J* = 7.6, 0.5 Hz, 1H), 6.93–6.89 (m, 1H), 7.02–6.97 (m, 1H), 7.24 (d, *J* = 7.5 Hz, 1H), 7.33–7.30 (m, 1H), 7.37–7.33 (m, 1H), 7.41 (dd, *J* = 7.6, 0.9 Hz, 1H), 8.02–7.98 (m, 1H);^13^C-NMR (150 MHz, CDCl_3_) δ 165.5 (CO), 139.3 (C, Ar), 139.1 (C, Ar), 131.3 (C, Ar), 129.8 (C, Ar), 128.4 (CH, Ar), 128.0 (CH, Ar), 127.6 (CH, Ar), 125.0 (CH, Ar), 123.2 (CH, Ar), 121.6 (CH, Ar), 116.6 (CH, Ar), 112.0 (CH, Ar), 82.3 (C), 38.7 (CH_2_), 29.5 (CH_3_); ESI-LRMS *m*/*z*: 251 [M + H]^+^; ESI-HRMS *m*/*z* calcd for M + H^+^ 251.1179, found: 251.1178. The characterization data is in accordance with that reported in [43].

*3a-Methyl-3,3a-dihydro-1H-benzo[d]pyrrolo[2,1-b][1,3]oxazine-1,5(2H)-dione* (**SF35a**): white solid (98.9 mg, yield 91%), mp 114–115 °C. ^1^H-NMR (400 MHz, DMSO-*d*_6_) δ 1.63 (s, 3H), 2.48–2.41 (m, 2H), 2.68–2.59 (m, 1H), 2.82–2.71 (m, 1H), 7.44–7.36 (m, 1H), 7.83–7.76 (m, 1H), 8.08–7.92 (m, 2H); ^13^C-NMR (125 MHz, DMSO-*d*_6_) δ 171.7 (CO), 161.1 (CO), 136.1 (C, Ar), 135.7 (CH, Ar), 129.9 (CH, Ar), 125.5 (CH, Ar), 120.5 (CH, Ar), 115.7 (C, Ar), 95.4 (C), 31.7 (CH_2_), 29.1 (CH_2_), 24.0 (CH_3_); ESI-LRMS *m*/*z*: 218 [M + H]^+^; ESI-HRMS *m*/*z* calcd for M + H^+^ 218.0812, found: 218.0809. The characterization data is in accordance with that reported in [43].

*2-Hexyl-3a-methyl-3,3a-dihydro-1H-benzo[d]pyrrolo[2,1-b][1,3]oxazine-1,5(2H)-dione* (**SF35b**): colorless oil (75.4 mg, yield 50% (dr = 5.5:1)), and the two diastereomers were inseparable by chromatography. ^1^H-NMR (600 MHz, DMSO-*d*_6_) for the major isomer: δ 0.91–0.84 (m, 3H), 1.42–1.21 (m, 9H), 1.65–1.61 (m, 3H), 1.85–1.78 (m, 1H), 2.13–2.07 (m, 1H), 2.71–2.62 (m, 1H), 2.91–2.83 (m, 1H), 7.41–7.37 (m, 1H), 7.81–7.78 (m, 1H), 8.00 (dd, *J* = 7.8, 1.5 Hz, 1H), 8.10–8.07 (m, 1H); ^13^C-NMR (150 MHz, DMSO-*d*_6_) for major isomer: δ 173.0 (CO), 161.0 (CO), 136.0 (CH, Ar), 135.8 (CH, Ar), 129.9 (CH, Ar), 125.3 (CH, Ar), 119.8 (CH, Ar), 115.1 (C, Ar), 93.5 (C, Ar), 39.8 (CH), 38.5 (CH_2_), 31.2 (CH_2_), 29.6 (CH_2_), 28.6 (CH_2_), 26.3 (CH_2_), 23.5 (CH_3_), 22.1 (CH_2_), 14.0 (CH_3_); ESI-LRMS *m*/*z*: 302 [M + H]^+^; ESI-HRMS *m*/*z* calcd for M + H^+^ 302.1751, found: 302.1750.

*4a-Methyl-2,3,4,4a-tetrahydrobenzo[d]pyrido[2,1-b][1,3]oxazine-1,6-dione* (**SF36**): colorless oil (88.5 mg, yield 77%). ^1^H-NMR (500 MHz, DMSO-*d*_6_) δ 1.55 (s, 3H), 1.86–1.77 (m, 1H), 1.95–1.86 (m, 1H), 2.31–2.21 (m, 2H), 2.55–2.48 (m, 1H), 2.68–2.58 (m, 1H), 7.46–7.39 (m, 1H), 7.77–7.70 (m, 2H), 7.98–7.93 (m, 1H); ^13^C-NMR (125 MHz, DMSO-*d*_6_) δ 169.0 (CO), 161.8 (CO), 138.6 (C, Ar), 134.1 (CH, Ar), 128.7 (CH, Ar), 126.1 (2 × CH, Ar), 119.9 (C, Ar), 92.7 (C), 34.9 (CH_2_), 32.9 (CH_2_), 25.9 (CH_3_), 16.1 (CH_2_); ESI-LRMS *m*/*z*: 232 [M + H]^+^; ESI-HRMS *m*/*z* calcd for M + H^+^ 232.0968, found: 232.0965. The characterization data is in accordance with that reported in ref.43. The characterization data is in accordance with that reported in [43].

*6a-Methyl-5H-benzo[4,5][1,3]oxazino[2,3-a]isoindole-5,11(6aH)-dione* (**SF37**): pale yellow solid (83.6 mg, yield 63%), mp 138–139 °C. ^1^H-NMR (400 MHz, DMSO-*d*_6_) δ 1.94 (s, 3H), 7.51–7.45 (m, 1H), 7.79–7.73 (m, 1H), 7.93–7.85 (m, 2H), 7.95 (d, *J* = 7.5 Hz, 1H), 8.06–8.00 (m, 2H), 8.08 (dd, *J* = 7.8, 1.2 Hz, 1H); ^13^C-NMR (125 MHz, DMSO-*d*_6_) δ 164.1 (CO), 161.3 (CO), 143.8 (C, Ar), 136.0 (CH, Ar), 135.7 (C, Ar), 134.4 (CH, Ar), 131.3 (CH, Ar), 130.2 (CH, Ar), 129.4 (C, Ar), 125.7 (CH, Ar), 124.2 (CH, Ar), 123.2 (CH, Ar), 121.2 (CH, Ar), 115.3 (C, Ar), 92.5 (C), 23.4 (CH_3_); ESI-LRMS *m*/*z*: 266 [M + H]^+^; ESI-HRMS *m*/*z* calcd for M + H^+^ 266.0812, found: 266.0807. The characterization data is in accordance with that reported in [43].

*3a-Methyl-3,3a-dihydro-1H-naphtho[2,3-d]pyrrolo[2,1-b][1,3]oxazine-1,5(2H)-dione* (**SF38**): yellow solid (128.1 mg, yield 96%), mp 228–230 °C. ^1^H-NMR (600 MHz, CDCl_3_) δ 1.72 (s, 3H), 2.50–2.42 (m, 1H), 2.68–2.61 (m, 1H), 2.76–2.68 (m, 1H), 2.84–2.77 (m, 1H), 7.56–7.51 (m, 1H), 7.67–7.62 (m, 1H), 7.91 (d, *J* = 8.3 Hz, 1H), 7.95 (d, *J* = 8.2 Hz, 1H), 8.44 (s, 1H), 8.72 (s, 1H); ^13^C-NMR (150 MHz, CDCl_3_) δ 171.7 (CO), 162.3 (CO), 136.7 (C, Ar), 133.2 (CH, Ar), 131.1 (C, Ar), 130.5 (C, Ar), 129.9 (CH, Ar), 129.7 (CH, Ar), 128.0 (CH, Ar), 126.9 (CH, Ar), 119.4 (CH, Ar), 115.7 (C, Ar), 95.7 (C), 32.3 (CH_2_), 29.6 (CH_2_), 25.7 (CH_3_); ESI-LRMS *m*/*z*: 268 [M + H]^+^; ESI-HRMS *m*/*z* calcd for M + H^+^ 268.0968, found: 268.0962. The characterization data is in accordance with that reported in [35].

*4a-Methyl-2,3,4,4a-tetrahydronaphtho[2,3-d]pyrido[2,1-b][1,3]oxazine-1,6-dione* (**SF39**): pale yellow oil (103.7 mg, yield 74%). ^1^H-NMR (600 MHz, CDCl_3_) δ 1.64 (s, 3H), 1.94–1.87 (m, 1H), 2.22–2.12 (m, 2H), 2.52–2.46 (m, 1H), 2.74–2.61 (m, 2H), 7.57–7.53 (m, 1H), 7.66–7.61 (m, 1H), 7.90 (d, *J* = 8.3 Hz, 1H), 7.96 (d, *J* = 8.2 Hz, 1H), 8.22 (s, 1H), 8.67 (s, 1H); ^13^C-NMR (150 MHz, CDCl_3_) δ 169.3 (CO), 163.3 (CO), 135.9 (C, Ar), 133.0 (C, Ar), 131.8 (CH, Ar), 130.8 (C, Ar), 129.6 (CH, Ar), 129.5 (CH, Ar), 128.2 (CH, Ar), 127.1 (CH, Ar), 124.9 (CH, Ar), 119.2 (C, Ar), 92.5 (C), 36.3 (CH_2_), 33.7 (CH_2_), 27.7 (CH_3_), 16.7 (CH_2_); ESI-LRMS *m*/*z*: 282 [M + H]^+^; ESI-HRMS *m*/*z* calcd for M + H^+^ 282.1125, found: 282.1117. The characterization data is in accordance with that reported in [43].

*4b-Methyl-4bH-naphtho[2’,3’:4,5][1,3]oxazino[2,3-a]isoindole-6,14-dione* (**SF40**): white solid (113.2 mg, yield 72%), mp 210–212 °C. ^1^H-NMR (600 MHz, CDCl_3_) δ 1.98 (s, 3H), 7.58–7.54 (m, 1H), 7.69–7.64 (m, 2H), 7.78–7.74 (m, 1H), 7.81–7.79 (m, 1H), 8.02–7.95 (m, 3H), 8.49 (s, 1H), 8.79 (s, 1H); ^13^C-NMR (150 MHz, CDCl_3_) δ 164.7 (CO), 162.4 (CO), 144.0 (C, Ar), 136.9 (C, Ar), 133.9 (CH, Ar), 133.5 (CH, Ar), 131.1 (CH, Ar), 130.9 (C, Ar), 130.4 (C, Ar), 130.3 (C, Ar), 130.0 (CH, Ar), 129.9 (CH, Ar), 128.0 (CH, Ar), 126.8 (CH, Ar), 124.8 (CH, Ar), 122.6 (CH, Ar), 119.4 (CH, Ar), 115.0 (C, Ar), 92.7 (C), 25.0 (CH_3_); ESI-LRMS *m*/*z*: 316 [M + H]^+^; ESI-HRMS *m*/*z* calcd for M + H^+^ 316.0968, found: 316.0959. The characterization data is in accordance with that reported in [43].

*6a-Methyl-7,8-dihydro-5H-pyrido[2,3-d]pyrrolo[2,1-b][1,3]oxazine-5,9(6aH)-dione* (**SF41**): colorless oil (65.1 mg, yield 60%). ^1^H-NMR (600 MHz, CDCl_3_) δ 1.72 (s, 3H), 2.52–2.41 (m, 1H), 2.68–2.60 (m, 1H), 2.86–2.69 (m, 2H), 7.36 (dd, *J* = 7.7, 4.9 Hz, 1H), 8.42 (dd, *J* = 7.7, 1.9 Hz, 1H), 8.81 (dd, *J* = 4.9, 1.9 Hz, 1H); ^13^C-NMR (150 MHz, CDCl_3_) δ 171.6 (CO), 161.3 (CO), 155.1 (CH, Ar), 149.1 (C, Ar), 139.4 (CH, Ar), 122.0 (CH, Ar), 113.0 (C, Ar), 96.1 (C), 32.3 (CH_2_), 29.8 (CH_2_), 25.4 (CH_3_); ESI-LRMS *m*/*z*: 219 [M + H]^+^; ESI-HRMS *m*/*z* calcd for M + H^+^ 219.0764, found: 219.0763. The characterization data is in accordance with that reported in [35].

*3a-Methyl-2,3,3a,5-tetrahydro-1H-benzo[d]pyrrolo[2,1-b][1,3]oxazin-1-one* (**SF42**): pale yellow oil (33.8 mg, yield 33%). ^1^H-NMR (500 MHz, DMSO-*d*_6_) δ 1.45 (s, 3H), 2.07–1.97 (m, 1H), 2.29–2.19 (m, 1H), 2.43 (ddd, *J* = 17.1, 10.0, 2.1 Hz, 1H), 2.76–2.65 (m, 1H), 4.88 (d, *J* = 16.0 Hz, 1H), 4.99 (d, *J* = 16.0 Hz, 1H), 7.16–7.10 (m, 1H), 7.21–7.16 (m, 1H), 7.32–7.25 (m, 1H), 8.20 (d, *J* = 8.1 Hz, 1H); ^13^C-NMR (125 MHz, DMSO-*d*_6_) δ 170.9 (CO), 132.6 (C, Ar), 127.1 (CH, Ar), 124.7 (CH, Ar), 123.7 (CH, Ar), 123.2 (C, Ar), 119.2 (CH, Ar), 89.6 (C), 62.0 (CH_2_), 32.5 (CH_2_), 29.7 (CH_2_), 20.7 (CH_3_); ESI-LRMS *m*/*z*: 204 [M + H]^+^; ESI-HRMS *m*/*z* calcd for M + H^+^ 204.1019, found: 204.1017. The characterization data is in accordance with that reported in [43].

*6a-Methyl-5H-benzo[4,5][1,3]oxazino[2,3-a]isoindol-11(6aH)-one* (**SF43**): colorless oil (22.4 mg, yield 18%). ^1^H-NMR (600 MHz, CDCl_3_) δ 1.74 (s, 3H), 4.98 (d, *J* = 15.3 Hz, 1H), 5.20 (d, *J* = 15.3 Hz, 1H), 7.14–7.10 (m, 1H), 7.19–7.15 (m, 1H), 7.40–7.35 (m, 1H), 7.59–7.54 (m, 1H), 7.68–7.60 (m, 2H), 7.91 (d, *J* = 7.5 Hz, 1H), 8.25 (d, *J* = 8.2 Hz, 1H); ^13^C-NMR (150 MHz, CDCl_3_) δ 165.2 (CO), 146.1 (C, Ar), 133.1 (CH, Ar), 132.6 (C, Ar), 131.2 (C, Ar), 130.2 (CH, Ar), 127.9 (CH, Ar), 124.3 (2 × CH, Ar), 124.2 (CH, Ar), 123.0 (C, Ar), 121.8 (CH, Ar), 121.5 (CH, Ar), 88.1 (C), 63.3 (CH_2_), 20.8 (CH_3_); ESI-LRMS *m*/*z*: 252 [M + H]^+^; ESI-HRMS *m*/*z* calcd for M + H^+^ 252.1019, found: 252.1018. The characterization data is in accordance with that reported in [43].

### 3.4. General Procedure of the Reductive Preparation of Compounds **SF47**–**SF55**

To a solution of substrates (0.3 mmol) in dry THF was added AlCl_3_ (0.6 mmol), then LiAlH_4_ (0.6 mmol) was added portionwise at 0 °C. After that, the mixture was heated to reflux for 4 h. After the reaction was cooled, the reaction mixture was diluted with dichloromethane (120.0 mL), and then water was added dropwise at 0 °C to quench the reaction under vigorous stirring conditions. The solid which precipitated out was removed by filtration, and the organic layer obtained was dried over Na_2_SO_4_. After the removal of the solvents in vacuo, the residue was purified to give **SF47**–**SF55**.

*11b-Methyl-2,3,5,6,11,11b-hexahydro-1H-indolizino[8,7-b]indole* (**SF47**): pale yellow oil (40.5 mg, yield 60%). ^1^H-NMR (400 MHz, DMSO-*d*_6_) δ 1.67–1.54 (m, 1H), 1.80 (s, 3H), 2.04–1.93 (m, 1H), 2.20–2.08 (m, 1H), 2.94–2.85 (m, 1H), 3.07–2.96 (m, 1H), 3.30–3.22 (m, 2H), 3.56–3.47 (m, 3H), 7.05–6.99 (m, 1H), 7.15–7.09 (m, 1H), 7.35 (d, *J* = 8.1 Hz, 1H), 7.47 (d, *J* = 7.8 Hz, 1H), 11.28 (s, 1H); ^13^C-NMR (125 MHz, DMSO-*d*_6_) δ 136.3 (C, Ar), 125.7 (C, Ar), 121.8 (CH, Ar), 119.0 (CH, Ar), 118.3 (CH, Ar), 111.3 (CH, Ar), 104.5 (C, Ar), 48.9 (CH_2_), 42.5 (CH_2_), 36.4 (CH_2_), 24.1 (CH_3_), 21.0 (CH_2_), 15.3 (CH_2_); ESI-LRMS *m*/*z*: 227 [M + H]^+^; ESI-HRMS *m*/*z* calcd for M + H^+^ 227.1543, found: 227.1540.

*13b-Methyl-7,8,13,13b-tetrahydro-5H-benzo[1,2]indolizino[8,7-b]indole* (**SF48a**): pale yellow solid (52.3 mg, yield 64%), mp 204–206 °C. ^1^H-NMR (400 MHz, CDCl_3_) δ 1.95 (s, 3H), 2.62–2.53 (m, 1H), 3.28–3.13 (m, 1H), 3.54–3.38 (m, 2H), 4.27–4.18 (m, 2H), 7.13–7.03 (m, 2H), 7.21–7.13 (m, 2H), 7.31–7.22 (m, 2H), 7.85–7.74 (m, 2H), 7.93 (s, 1H); ^13^C-NMR (125 MHz, DMSO-*d*_6_) δ 135.5 (C, Ar), 131.8 (C, Ar), 126.5 (CH, Ar), 126.4 (CH, Ar), 124.9 (C, Ar), 122.8 (CH, Ar), 122.4 (CH, Ar), 120.2 (CH, Ar), 119.0 (CH, Ar), 118.3 (CH, Ar), 110.8 (CH, Ar), 52.7 (CH_2_), 41.0 (CH_2_), 26.2 (CH_3_), 17.1 (CH_2_); ESI-LRMS *m*/*z*: 275 [M + H]^+^; ESI-HRMS *m*/*z* calcd for M + H^+^ 275.1543, found: 275.1541. The characterization data is in accordance with that reported in [43].

*10-Methoxy-13b-methyl-7,8,13,13b-tetrahydro-5H-benzo[1,2]indolizino[8,7-b]indole* (**SF48b**): pale yellow oil (59.2 mg, yield 65%). ^1^H-NMR (400 MHz, CDCl_3_) δ 1.86 (s, 3H), 2.60 (ddd, *J* = 15.8, 4.3, 1.4 Hz, 1H), 3.13 (ddd, *J* = 16.2, 11.1, 6.4 Hz, 1H), 3.54–3.43 (m, 2H), 3.84 (s, 3H), 4.26–4.15 (m, 2H), 6.78 (dd, *J* = 8.7, 2.5 Hz, 1H), 6.93 (d, *J* = 2.4 Hz, 1H), 7.15 (d, *J* = 8.7 Hz, 1H), 7.25–7.18 (m, 2H), 7.33–7.27 (m, 1H), 7.45 (d, *J* = 7.4 Hz, 1H), 7.60 (s, 1H); ^13^C-NMR (100 MHz, CDCl_3_) δ 154.2 (C, Ar), 144.9 (C, Ar), 139.0 (C, Ar), 137.8 (C, Ar), 131.3 (C, Ar), 127.7 (CH, Ar), 127.7 (C, Ar), 127.4 (CH, Ar), 123.4 (CH, Ar), 121.1 (CH, Ar), 111.7 (CH, Ar), 111.7 (CH, Ar), 106.9 (C, Ar), 100.9 (CH, Ar), 65.0 (C), 56.1 (OCH_3_), 53.2 (CH_2_), 41.7 (CH_2_), 26.0 (CH_3_), 16.4 (CH_2_); ESI-LRMS *m*/*z*: 305 [M + H]^+^; ESI-HRMS *m*/*z* calcd for M + H^+^ 305.1648, found: 305.1645. The characterization data is in accordance with that reported in [43].

*14b-Methyl-5,6,8,9,14,14b-hexahydroindolo[2’,3’:3,4]pyrido[2,1-a]isoquinoline* (**SF49**): pale yellow oil (60.2 mg, yield 70%). ^1^H-NMR (400 MHz, CDCl_3_) δ 1.95 (s, 3H), 2.85–2.66 (m, 2H), 3.07–2.91 (m, 2H), 3.14–3.07 (m, 1H), 3.19–3.15 (m, 1H), 3.26 (ddd, *J* = 13.8, 6.1, 2.6 Hz, 1H), 3.76 (ddd, *J* = 13.9, 10.0, 5.8 Hz, 1H), 7.19–7.05 (m, 3H), 7.25–7.20 (m, 1H), 7.31–7.27 (m, 1H), 7.37–7.31 (m, 1H), 7.49 (d, *J* = 7.6 Hz, 1H), 7.65 (d, *J* = 7.8 Hz, 1H), 7.87 (s, 1H); ^13^C-NMR (100 MHz, CDCl_3_) δ140.0 (C, Ar), 137.5 (C, Ar), 135.8 (C, Ar), 134.5 (C, Ar), 130.1 (CH, Ar), 127.7 (C, Ar), 126.8 (CH, Ar), 126.6 (CH, Ar), 126.0 (CH, Ar), 121.8 (CH, Ar), 119.6 (CH, Ar), 118.5 (CH, Ar), 111.0 (CH, Ar), 105.9 (C, Ar), 58.3 (C), 46.8 (CH_2_), 45.5 (CH_2_), 30.4 (CH_3_), 28.8 (CH_2_), 17.9 (CH_2_); ESI-LRMS *m*/*z*: 289 [M + H]^+^; ESI-HRMS *m*/*z* calcd for M + H^+^ 289.1699, found: 289.1695. The characterization data is in accordance with that reported in [43].

*11c-Methyl-2,3,5,6,7,11c-hexahydro-1H-indolizino[7,8-b]indole* (**SF50**): pale yellow solid (62.2 mg, yield 92%), mp 249–250 °C. ^1^H-NMR (400 MHz, DMSO-*d*_6_) δ 1.66–1.51 (m, 1H), 1.82 (s, 3H), 2.04–1.92 (m, 1H), 2.27–2.15 (m, 1H), 2.61–2.52 (m, 1H), 3.02–2.87 (m, 1H), 3.24–3.13 (m, 1H), 3.31–3.25 (m, 1H), 3.60–3.44 (m, 3H), 7.04–6.97 (m, 1H), 7.13–7.07 (m, 1H), 7.35 (d, *J* = 8.0 Hz, 1H), 7.51 (d, *J* = 7.9 Hz, 1H), 11.24 (s, 1H); ^13^C-NMR (125 MHz, DMSO-*d*_6_) δ 136.2 (C, Ar), 129.5 (C, Ar), 123.3 (C, Ar), 121.3 (CH, Ar), 119.0 (CH, Ar), 118.0 (CH, Ar), 111.4 (CH, Ar), 48.7 (CH_2_), 41.8 (CH_2_), 36.1 (CH_2_), 24.3 (CH_3_), 21.2 (CH_2_), 16.8 (CH_2_); ESI-LRMS *m*/*z*: 227 [M + H]^+^; ESI-HRMS *m*/*z* calcd for M + H^+^ 227.1543, found: 227.1541.

*14c-Methyl-5,6,8,9,10,14c-hexahydroindolo[3’,2’:3,4]pyrido[2,1-a]isoquinoline* (**SF51**): pale yellow oil (63.5 mg, yield 73%). ^1^H-NMR (600 MHz, CDCl_3_) δ 2.21 (s, 3H), 2.82–2.75 (m, 1H), 2.97 (dd, *J* = 17.9, 6.5 Hz, 1H), 3.23–3.17 (m, 1H), 3.32–3.27 (m, 2H), 3.42–3.38 (m, 1H), 3.50–3.43 (m, 1H), 4.15–4.07 (m, 1H), 7.10–7.05 (m, 2H), 7.14–7.10 (m, 2H), 7.20–7.16 (m, 1H), 7.31 (d, *J* = 8.0 Hz, 1H), 7.65 (d, *J* = 7.9 Hz, 1H), 7.79 (d, *J* = 7.9 Hz, 1H), 9.64 (s, 1H); ^13^C-NMR (150 MHz, CDCl_3_) δ 142.9 (C, Ar), 136.6 (C, Ar), 132.7 (C, Ar), 131.2 (C, Ar), 129.2 (CH, Ar), 128.1 (CH, Ar), 127.3 (C, Ar), 126.3 (2 × CH, Ar), 120.8 (CH, Ar), 120.7 (CH, Ar), 119.5 (CH, Ar), 115.3 (C, Ar), 111.2 (CH, Ar), 59.8 (C), 45.8 (CH_2_), 45.3 (CH_2_), 29.9 (CH_3_), 23.6 (CH_2_), 23.1 (CH_2_); ESI-LRMS *m*/*z*: 289 [M + H]^+^; ESI-HRMS *m*/*z* calcd for M + H^+^ 289.1699, found: 289.1698.

*15b-Methyl-6,8,9,15b-tetrahydro-5H-indolo[2’,1’:3,4]pyrazino[2,1-a]isoquinoline* (**SF52**): pale yellow oil (34.3 mg, yield 40%). ^1^H-NMR (600 MHz, CDCl_3_) δ 1.94 (s, 3H), 2.92–2.83 (m, 1H), 3.07–2.99 (m, 1H), 3.17–3.09 (m, 1H), 3.40–3.32 (m, 1H), 3.55–3.47 (m, 1H), 3.63–3.56 (m, 1H), 4.08–4.01 (m, 1H), 4.15–4.10 (m, 1H), 6.55 (s, 1H), 7.15–7.10 (m, 2H), 7.20–7.15 (m, 3H), 7.27 (d, *J* = 8.0 Hz, 1H), 7.63–7.57 (m, 2H); ^13^C-NMR (150 MHz, CDCl_3_) δ 140.9 (C, Ar), 139.8 (C, Ar), 136.7 (C, Ar), 132.8 (C, Ar), 129.5 (CH, Ar), 128.0 (C, Ar), 127.8 (CH, Ar), 126.6 (CH, Ar), 126.1 (CH, Ar), 121.2 (CH, Ar), 120.3 (CH, Ar), 120.1 (CH, Ar), 109.1 (CH, Ar), 100.6 (CH, Ar), 59.2 (C), 45.7 (CH_2_), 45.6 (CH_2_), 40.4 (CH_2_), 31.5 (CH_3_), 25.3 (CH_2_); ESI-LRMS *m*/*z*: 289 [M + H]^+^; ESI-HRMS *m*/*z* calcd for M + H^+^ 289.1699, found: 289.1696.

*11b-Methyl-4,5,7,11b-tetrahydro-3H-pyrrolo[3’,2’:3,4]pyrido[2,1-a]isoindole* (**SF53**): pale yellow oil (57.2 mg, yield 85%). ^1^H-NMR (600 MHz, CDCl_3_) δ 1.78 (s, 3H), 3.07–2.97 (m, 1H), 3.49–3.36 (m, 2H), 3.72–3.65 (m, 1H), 4.18–4.11 (m, 1H), 4.28–4.20 (m, 1H), 6.08–6.04 (m, 1H), 6.62–6.58 (m, 1H), 7.19–7.14 (m, 2H), 7.29–7.23 (m, 1H), 7.45–7.40 (m, 1H), 7.83 (s, 1H); ^13^C-NMR (150 MHz, CDCl_3_) δ 148.1 (C, Ar), 137.8 (C, Ar), 127.2 (CH, Ar), 126.8 (CH, Ar), 122.9 (C, Ar), 122.7 (CH, Ar), 121.9 (1CH + 1C, Ar), 116.6 (CH, Ar), 105.2 (CH, Ar), 65.8 (C), 53.5 (CH_2_), 42.1 (CH_2_), 28.2 (CH_3_), 17.2 (CH_2_); ESI-LRMS *m*/*z*: 225 [M + H]^+^; ESI-HRMS *m*/*z* calcd for M + H^+^ 225.1386, found: 225.1385.

*11b-Methyl-4,5,7,11b-tetrahydrothieno[3’,2’:3,4]pyrido[2,1-a]isoindole* (**SF54**): pale yellow solid (57.9 mg, yield 80%), mp 73–74 °C. ^1^H-NMR (600 MHz, CDCl_3_) δ 1.76 (s, 3H), 2.60 (ddd, *J* = 16.5, 4.4, 1.5 Hz, 1H), 3.22–3.13 (m, 1H), 3.49–3.38 (m, 2H), 4.20–4.12 (m, 2H), 6.94 (d, *J* = 5.2 Hz, 1H), 7.00 (dd, *J* = 5.3, 0.7 Hz, 1H), 7.21–7.17 (m, 2H), 7.28–7.25 (m, 1H), 7.45 (d, *J* = 7.6 Hz, 1H); ^13^C-NMR (150 MHz, CDCl_3_) δ 146.7 (C, Ar), 139.5 (C, Ar), 138.6 (C, Ar), 132.4 (C, Ar), 127.1 (2 × CH, Ar), 125.6 (CH, Ar), 122.8 (CH, Ar), 122.3 (CH, Ar), 122.1 (CH, Ar), 67.0 (C, Ar), 53.6 (CH_2_), 42.1 (CH_2_), 27.9 (CH_3_), 19.2 (CH_2_); ESI-LRMS *m*/*z*: 242 [M + H]^+^; ESI-HRMS *m*/*z* calcd for M + H^+^ 242.0998, found: 242.0997.

*11b-Methyl-4,5,7,11b-tetrahydrothieno[2’,3’:3,4]pyrido[2,1-a]isoindole* (**SF55**): yellow oil (68.6 mg, yield 95%). ^1^H-NMR (600 MHz, CDCl_3_) δ 1.85 (s, 3H), 2.47 (ddd, *J* = 16.4, 4.5, 1.4 Hz, 1H), 3.07–2.97 (m, 1H), 3.45–3.30 (m, 2H), 4.22–4.12 (m, 2H), 6.69 (d, *J* = 5.1 Hz, 1H), 7.11 (d, *J* = 5.0 Hz, 1H), 7.18–7.16 (m, 1H), 7.21–7.18 (m, 1H), 7.30–7.26 (m, 1H), 7.46 (d, *J* = 7.6 Hz, 1H); ^13^C-NMR (150 MHz, CDCl_3_) δ 147.2 (C, Ar), 142.4 (C, Ar), 138.4 (C, Ar), 132.5 (C, Ar), 127.3 (2 × CH, Ar), 126.9 (CH, Ar), 123.1 (CH, Ar), 122.8 (CH, Ar), 122.1 (CH, Ar), 67.0 (C), 53.7 (CH_2_), 41.8 (CH_2_), 29.8 (CH_3_), 20.2 (CH_2_); ESI-LRMS *m*/*z*: 242 [M + H]^+^; ESI-HRMS *m*/*z* calcd for M + H^+^ 242.0998, found: 242.0997.

## 4. Conclusions

In conclusion, a green and general tandem reaction between alkynoic acids and amine nucleophiles through gold catalysis in water has been developed. This process proceeds with high efficiency leading to the formation of two rings and three new bonds in a single operation. This approach features low catalyst loading, good to excellent yields, high efficiency in bond formation, high step economy, excellent selectivity, great functional group tolerance, and extraordinarily broad substrate scope, and has been successfully employed to construct a high-quality library of indole/thiophene/pyrrole/pyridine/naphthalene/benzene-fused *N*-heterocycles. In addition, five antimicrobial compounds were discovered from the library, suggesting the value of our strategy to identify APIs. This is the first example of the generation of pDOS compound library encompassing skeletal diversity, molecular complexity, and drug-like properties from readily available materials through gold catalysis in water. We anticipate that these valuable N-heterocycles will find more pharmaceutical applications after our further investigations.

## Figures and Tables

**Table 1:**
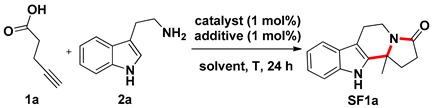
Reaction condition optimization for the tandem synthesis of compound **SF1a** ^a^.

Entry	Catalyst/Additive	Solvent	T (°C)	Yield (%) ^b^
1	-	H_2_O	100	17
2	PdCl_2_(CH_3_CN)_2_	H_2_O	100	26
3	Pd(PPh_3_)_2_Cl_2_	H_2_O	100	31
4	Pd(PPh_3_)_4_	H_2_O	100	0
5	Pd_2_(dba)_3_	H_2_O	100	0
6	Cu(OAc)_2_	H_2_O	100	trace
7	Cu(OH)_2_	H_2_O	100	5
8	CuCl	H_2_O	100	trace
9	NiOAc	H_2_O	100	33
10	Ni(PPh_3_)_2_Cl	H_2_O	100	11
11	Mn(OAc)_2_	H_2_O	100	25
12	Ag_2_CO_3_	H_2_O	100	51
13	AgOAc	H_2_O	100	57
14	[RuCl_2_(*p*-cym)]_2_	H_2_O	100	48
15	CoCl_2_	H_2_O	100	38
16	Co(acac)_2_	H_2_O	100	50
17	AuPPh_3_Cl	H_2_O	100	70
18 ^c^	AuPPh_3_Cl	H_2_O	100	76
19	AuPPh_3_Cl	H_2_O	120	83
20	AuBr_3_	H_2_O	120	52
21	AuI	H_2_O	120	46
22	Au^1^ catalyst ^d^	H_2_O	120	81
23	Au^2^ catalyst ^e^	H_2_O	120	78
24	Au^3^ catalyst ^f^	H_2_O	120	80
25	AuPPh_3_Cl/Ag_2_CO_3_	H_2_O	120	85
26	AuPPh_3_Cl/AgOAc	H_2_O	120	86
27	AuPPh_3_Cl/AgOTf	H_2_O	120	89
28	AuPPh_3_Cl/AgSbF_6_	H_2_O	120	91

^a^ Reaction conditions: 4-pentynoic acid **1a** (0.6 mmol), tryptamine **2a** (0.5 mmol), catalyst/additive (0.005 mmol), and solvent (4.0 mL). ^b^ Yield refers to isolated yield. ^c^ The reaction was performed for 48 h. ^d^ Au^1^ catalyst = Chloro[(1,1′-biphenyl-2-yl)di-*tert*-butylphosphine]gold(I). ^e^ Au^2^ catalyst = Chloro[1,3-bis(2,6-diisopropylphenyl)imidazol-2-ylidene]gold(I). ^f^ Au^3^ catalyst = (Acetonitrile)[(2-biphenyl)di-*tert*-butylphosphine]gold(I) hexafluoroantimonate.

**Table 2:** MIC_90_ of compounds **SF9d**, **SF29b**, **SF33**, **SF36**, and **SF41** against the *S. aureus* strain.

Compound	MIC_90_ (μg/mL)
**SF9d**	100–200
**SF29b**	50
**SF33**	100–200
**SF36**	10–25
**SF41**	100

## Supplementary Materials

The following are available online. Table S1: Survey of the solvents on the yield of product **SF1a**, Figures S1–S12: NMR (^1^H-NMR, ^13^C-NMR, HSQC, HMBC, and ^1^H-^1^H COSY) and ESI(+)MS spectrum of **SF5a**, **[D]_n_-SF5a**, **SF5b**, **[D]_n_-SF5b**, **SF1a**, and **[D]_n_-SF1a**. Figure S13: preliminary screening of antibacterial activities of compounds at 100 μg/mL. Figures S14–S19: time-kill results of **SF9d**, **SF29b**, **SF33**, **SF36**, and **SF41** again*st S. aureus* strain. Figures S20–S24: CFU results of compounds **SF9d**, **SF29b**, **SF33**, **SF36** and **SF41**; copies of ^1^H and ^13^C-NMR spectra of new compounds.
