# Supplementary material for: Green and Facile Assembly of Diverse Fused N-Heterocycles Using Gold-Catalyzed Cascade Reactions in Water
Source: Molecules. 2019 Mar 11;24(5):988. doi: 10.3390/molecules24050988 (PMC6429411; doi:10.3390/molecules24050988)

# Green and Facile Assembly of Diverse Fused N-Heterocycles using Gold-Catalyzed Cascade Reactions in Water

## Supplementary Materials

Xiuwen Jia[1], Pinyi Li[1], Xiaoyan Liu[1], Jiafu Lin[1*], Yiwen Chu[1], Jinhai Yu[2], Jiang Wang[3,4], Hong Liu[3,4*] and Fei Zhao[1*]

[1] Antibiotics Research and Re-evaluation Key Laboratory of Sichuan Province, Sichuan Industrial Institute of Antibiotics, Chengdu University, Chengdu 610052, China; jiaxiuwen2018@126.com (X.J.); pinyiLi19950206@126.com (P.L.); 19940826097@163.com (X.L.); siiakyb@139.com (Y.C.)

[2] School of Biological Science and Technology, University of Jinan, Jinan 250022, China; bio_yujh@ujn.edu.cn

[3] State Key Laboratory of Drug Research and CAS Key Laboratory of Receptor Research, Shanghai Institute of Materia Medica, Chinese Academy of Sciences, Shanghai 201203, China; jwang@simm.ac.cn

[4] University of Chinese Academy of Sciences, Beijing 100049, China.

***** Correspondence: linjiafu@cdu.edu.cn (J.L.); hliu@simm.ac.cn (H.L.); zhaofei@cdu.edu.cn (F.Z.); Tel.: +86-17360061902 (J.L.); Tel.: +86-021-5080-7042 (H.L.); Tel.: +86-18780255276 (F.Z.)

**Survey of the solvents on the yield of product SF1a……………………..………..2**

**NMR and ESI(+)MS spectrum of SF5a, [D]$_n$-SF5a, SF5b, [D]$_n$-SF5b, SF1a, [D]$_n$-SF1a…………………………………………………………………...…3**

**Antibacterial bioassay…………………………………………………….…..10**

**Antibacterial results and discussion……………………………………..…...11**

**Copies of $^1$H and $^{13}$C NMR spectra of new compounds…………..……….......…17**

**Table S1. Survey of the solvents on the yield of product SF1a [a].**

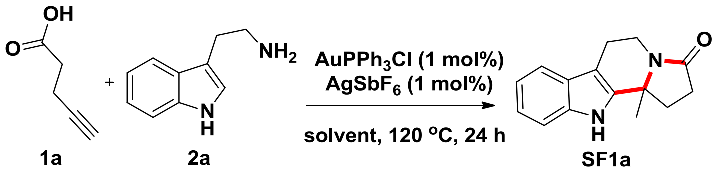


| Entry | Solvent | Yield (%) [b] |
|---|---|---|
| 1 | $H_2O$ | 91 |
| 2 | Toluene | 86 |
| 3 | Xylene | 88 |
| 4 | DCE | 90 |
| 5 | THF | 75 |
| 6 | $CH_3CN$ | 63 |
| 7 | DMSO | 41 |
| 8 | MeOH | 35 |

[a] Reaction conditions: 4-pentynoic acid **1a** (0.6 mmol), tryptamine **2a** (0.5 mmol), $AuPPh_3Cl/AgSbF_6$ (0.005 mmol), solvent (4.0 ml), 120 °C, 24 h. [b] Yield refers to isolated yield.

**NMR and ESI(+)MS spectrum of SF5a, [D]$_n$-SF5a, SF5b, [D]$_n$-SF5b, SF1a, [D]$_n$-SF1a**

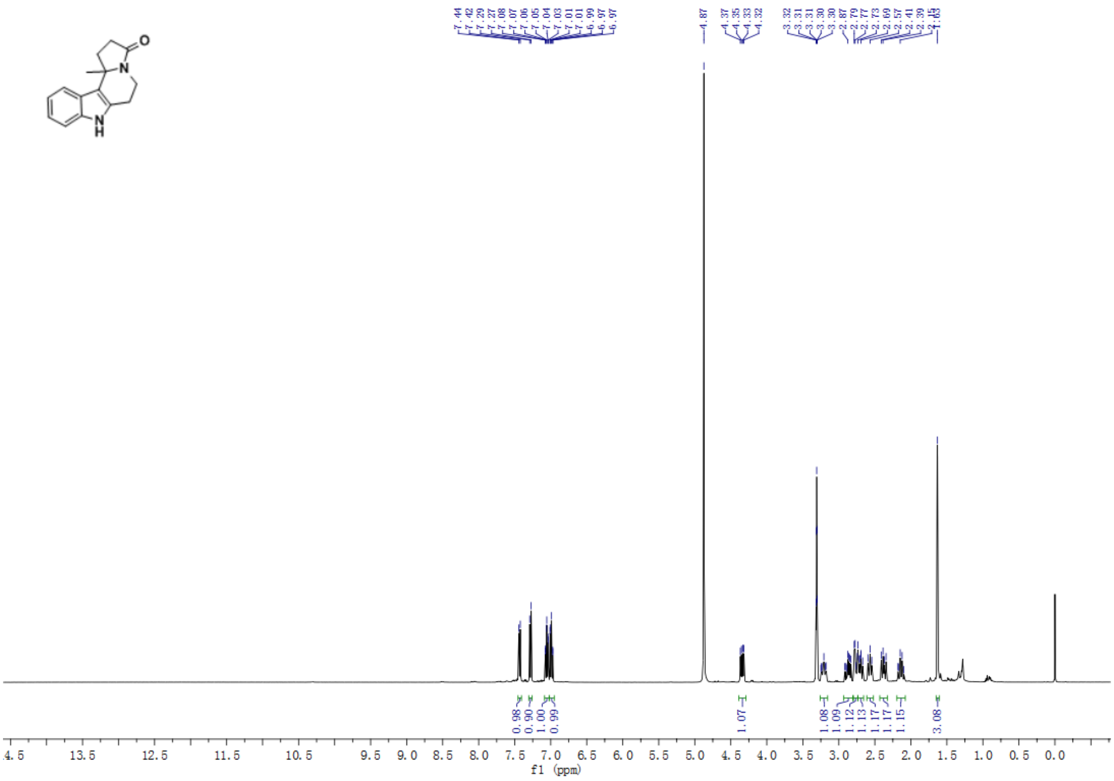


**Figure S1** $^{1}$H NMR spectrum of **SF5a** in methanol-$d_4$.

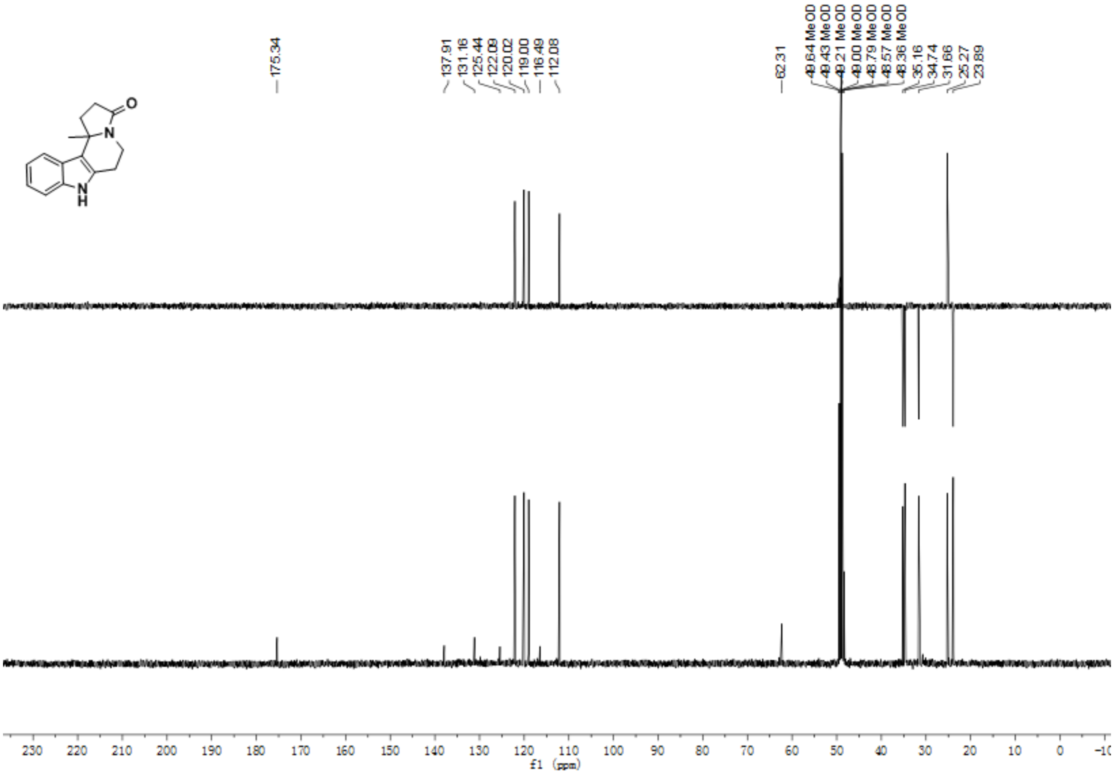


**Figure S2** $^{13}$C NMR spectrum of **SF5a** in methanol-$d_4$.

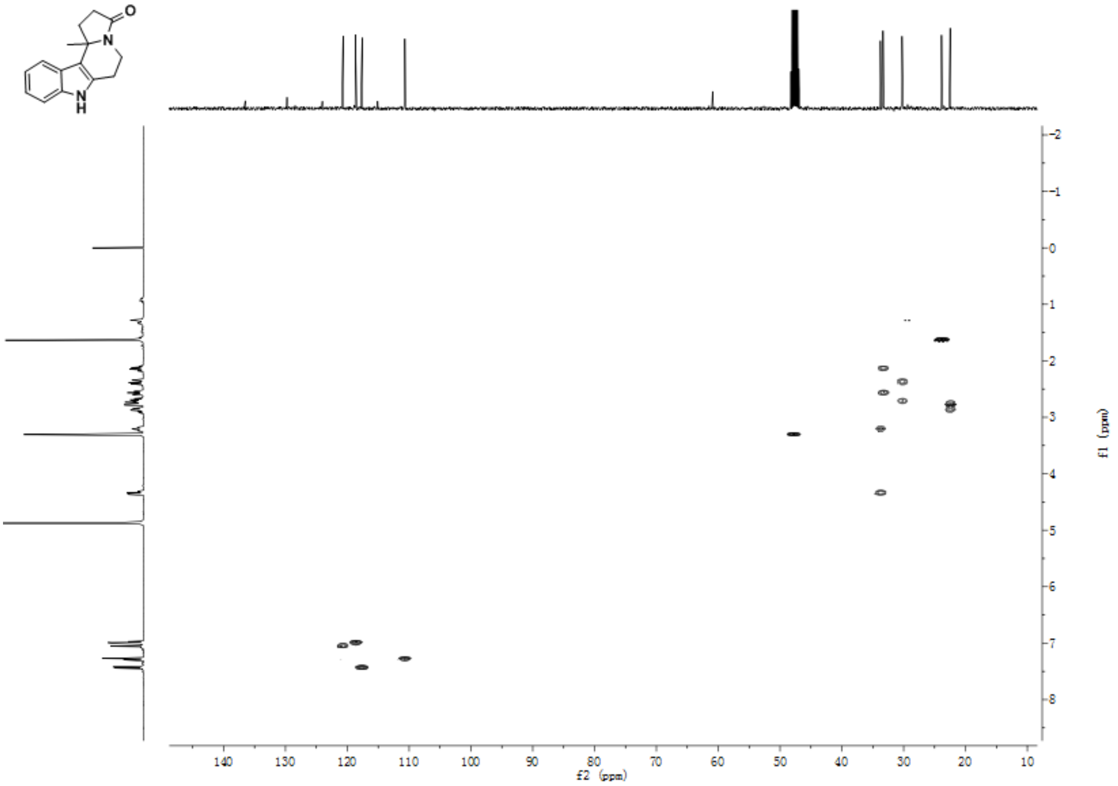


**Figure S3** HSQC spectrum of **SF5a** in methanol-$d_4$.

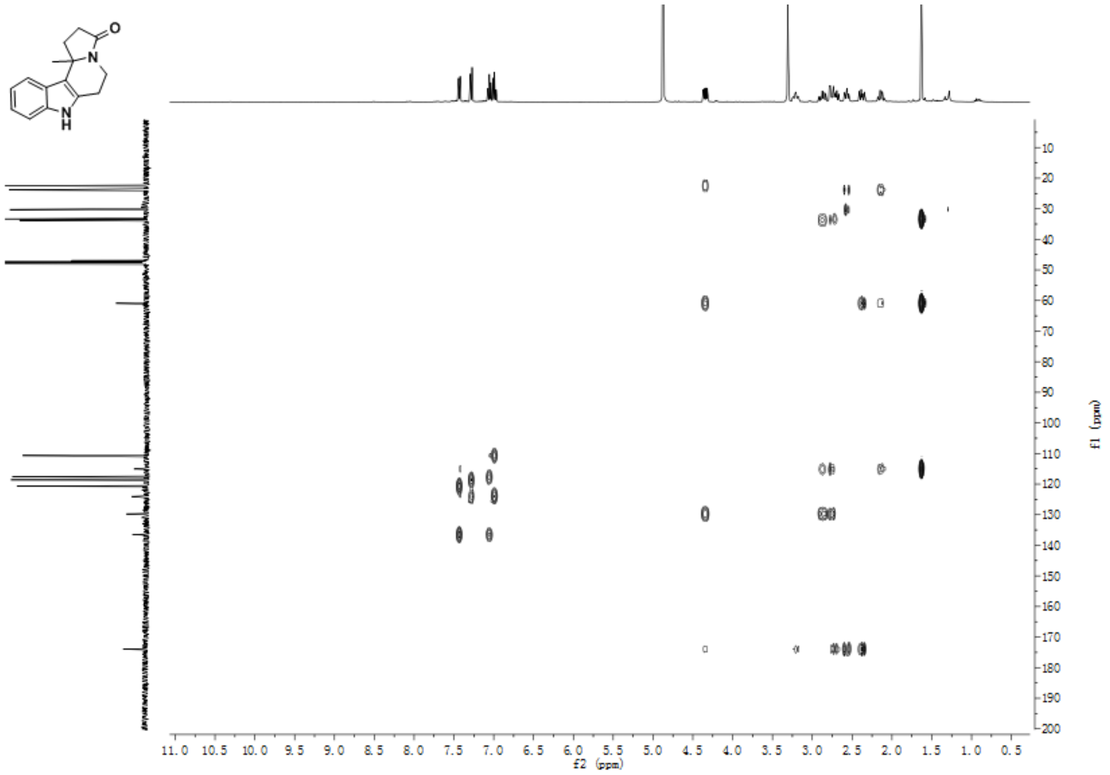


**Figure S4** HMBC spectrum of **SF5a** in methanol-$d_4$.

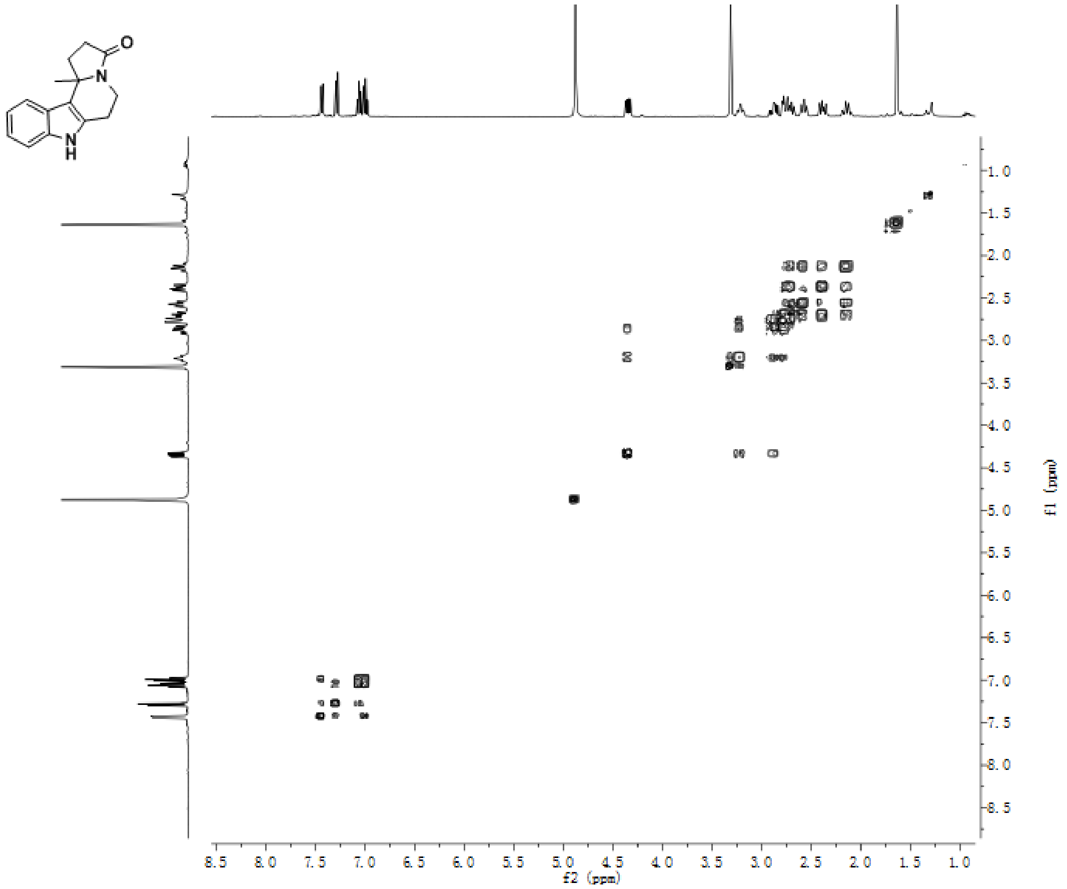

**Figure S5** $^{1}H$-$^{1}H$ COSY spectrum of **SF5a** in methanol-$d_4$.

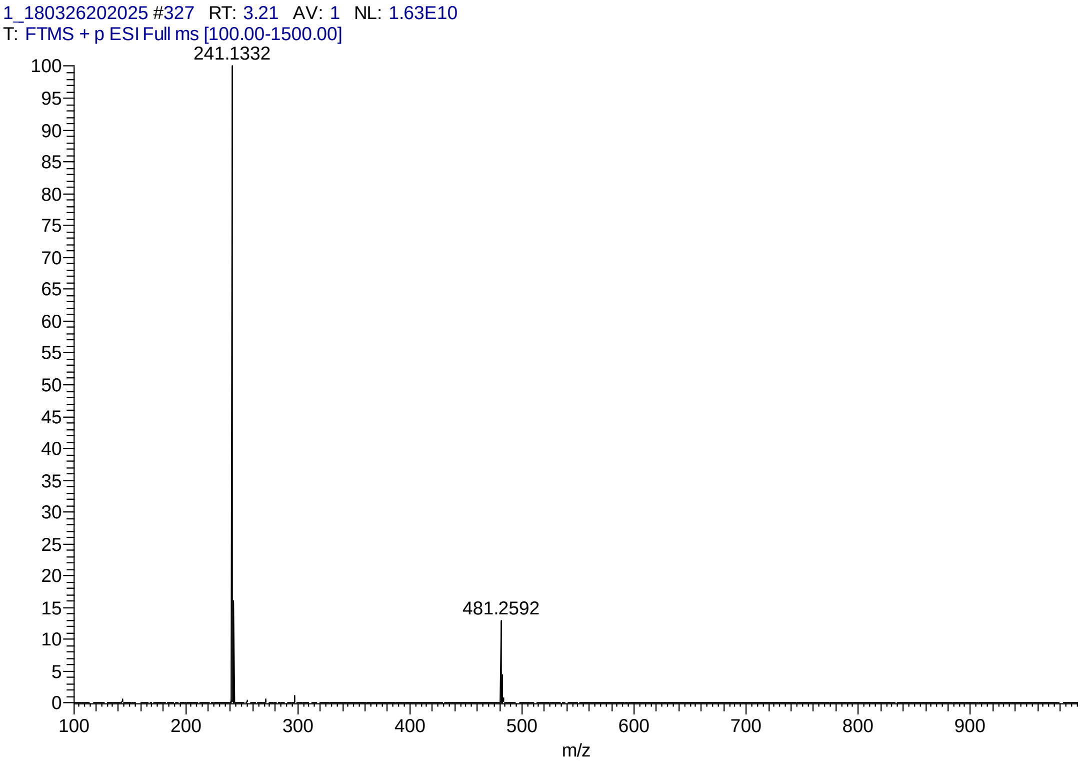


**Figure S6** ESI(+)MS spectrum of **SF5a**.

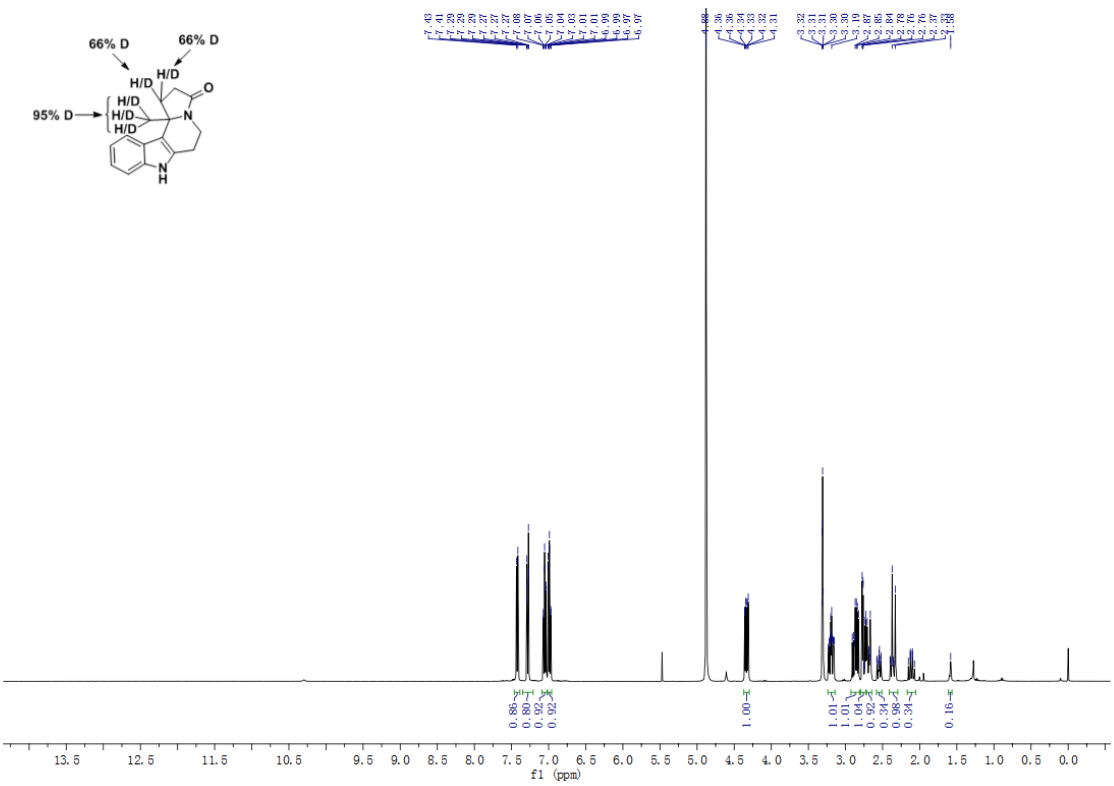

**Figure S7** $^{1}$H NMR spectrum of [D]$_n$-**SF5a** in methanol-$d_4$.

## Qualitative Analysis Report

| | | | |
|---|---|---|---|
| **Data Filename** | ESIH_20180323_LH_DWH_01.d | **Sample Name** | B6-ZF-0321 |
| **Sample Type** | Sample | **Position** | P1-B1 |
| **Instrument Name** | Agilent G6520 Q-TOF | **User Name** | |
| **Acq Method** | 20160322_MS_ESIH_POS_1min.m | **Acquired Time** | 3/23/2018 12:03:23 PM |
| **IRM Calibration Status** | Success | **DA Method** | small molecular data analysis method.m |
| **Comment** | ESIH by ZZY | | |

**Sample Group** Info.
**Acquisition SW** 6200 series TOF/6500 series
**Version** Q-TOF B.05.01 (B5125.3)

### User Spectra

| Fragmentor Voltage | Collision Energy | Ionization Mode |
|---|---|---|
| 100 | 0 | ESI |

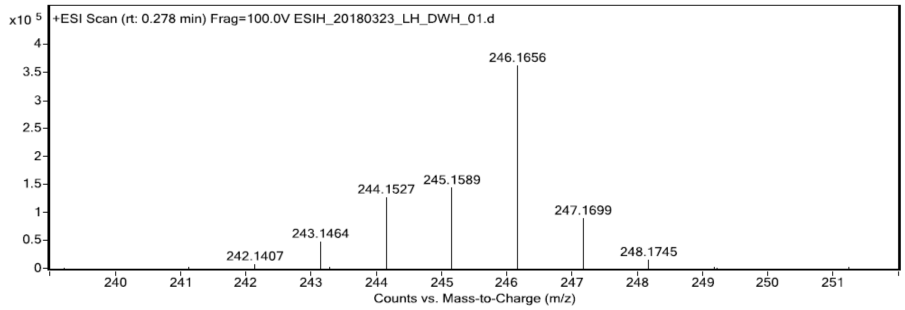


**Formula Calculator Element Limits**

| Element | Min | Max |
|---|---|---|
| C | 3 | 17 |
| H | 0 | 100 |
| O | 0 | 1 |
| N | 0 | 2 |
| S | 0 | 0 |
| D | 0 | 10 |



## Qualitative Analysis Report

**Formula Calculator Results**

| m/z | Calc m/z | Diff (mDa) | Diff (ppm) | Ion Formula | Score |
|---|---|---|---|---|---|
| 242.1407 | 242.1398 | -0.86 | -3.57 | C15 H16 D N2 O | 44.58 |
| 242.1407 | 242.1416 | 0.88 | 3.64 | C16 H4 D8 N O | 44.48 |
| 243.1464 | 243.1461 | -0.35 | -1.46 | C15 H15 D2 N2 O | 47.09 |
| 243.1464 | 243.1478 | 1.38 | 5.71 | C16 H3 D9 N O | 40.2 |
| 243.1464 | 243.1492 | 2.74 | 11.32 | C15 H19 N2 O | 24.48 |
| 244.1527 | 244.1524 | -0.36 | -1.49 | C15 H14 D3 N2 O | 47.07 |
| 244.1527 | 244.1541 | 1.38 | 5.66 | C16 H2 D10 N O | 40.3 |
| 244.1527 | 244.1555 | 2.73 | 11.24 | C15 H18 D N2 O | 24.63 |
| 245.1589 | 245.1586 | -0.22 | -0.9 | C15 H13 D4 N2 O | 47.42 |
| 245.1589 | 245.1617 | 2.88 | 11.78 | C15 H17 D2 N2 O | 23.02 |
| 245.1589 | 245.1556 | -3.32 | -13.59 | C15 H9 D6 N2 O | 18.1 |
| 246.1656 | 246.1649 | -0.99 | -4.06 | C15 H12 D5 N2 O | 82.39 |
| 246.1656 | 246.168 | 2.1 | 8.58 | C15 H16 D3 N2 O | 71.18 |

--- End Of Report ---



**Figure S8** ESI(+)MS spectrum of $[D]_n$-**SF5a**.

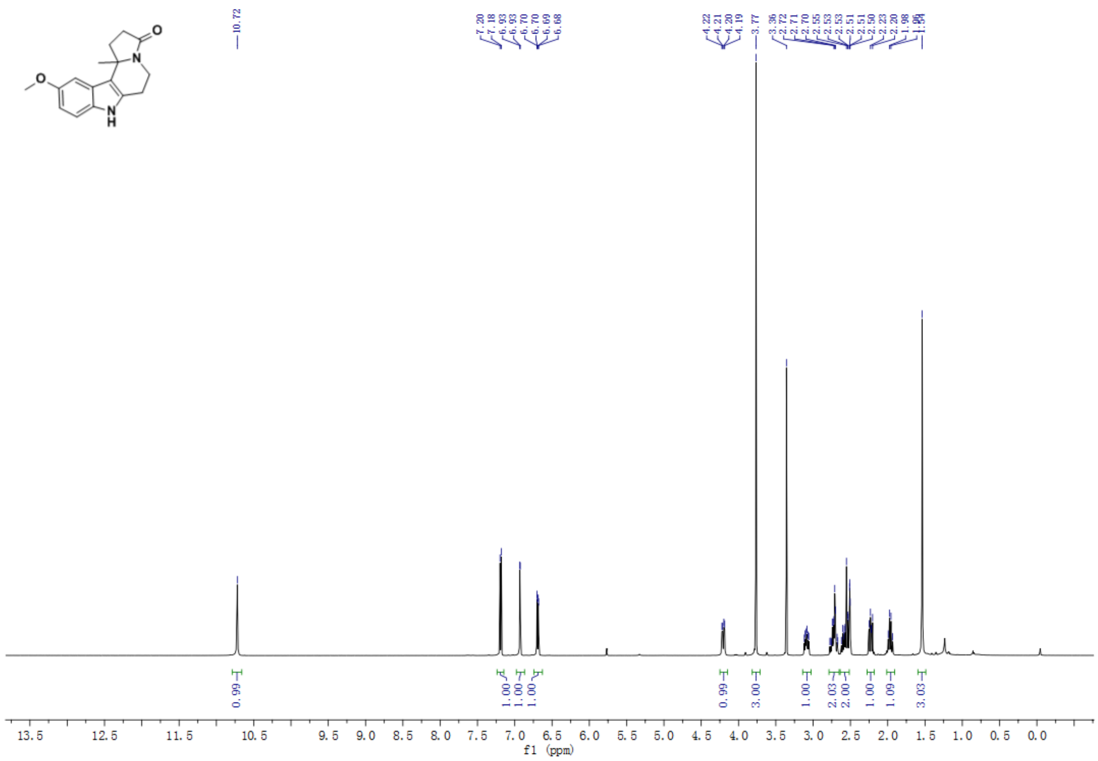

**Figure S9** $^{1}$H NMR spectrum of **SF5b** in dimethyl sulfoxide-$d_6$.

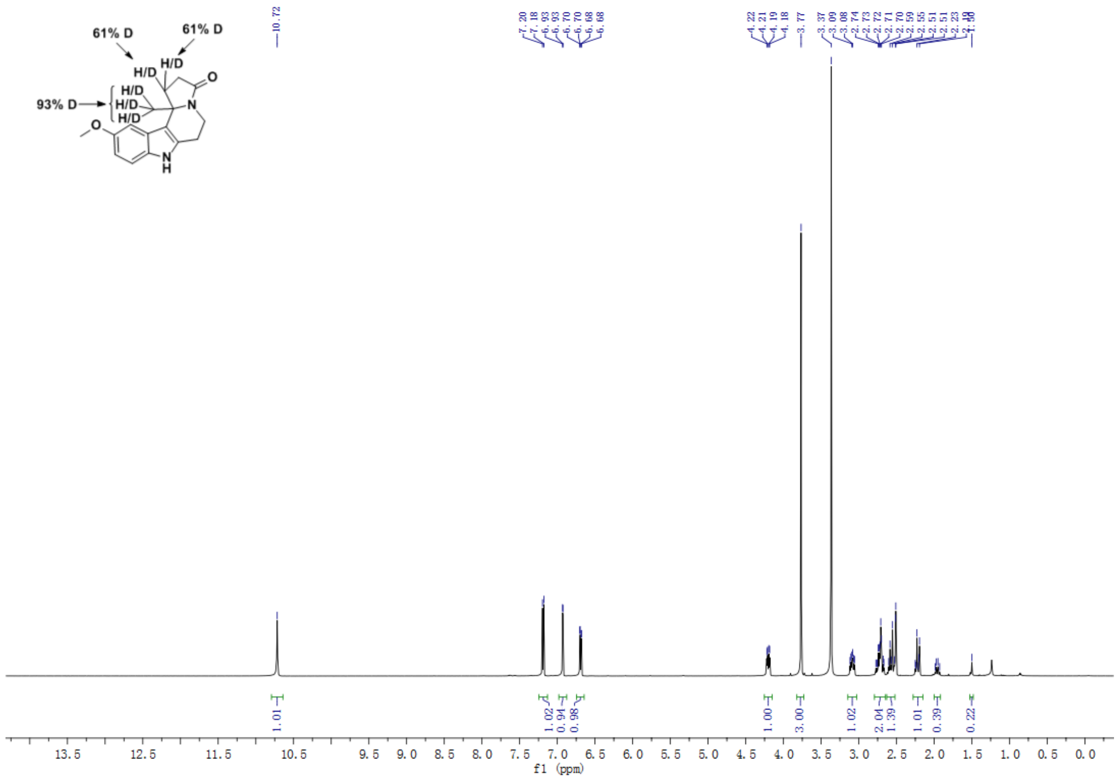

**Figure S10** $^{1}$H NMR spectrum of $[D]_n$-**SF5b** in dimethyl sulfoxide-$d_6$.

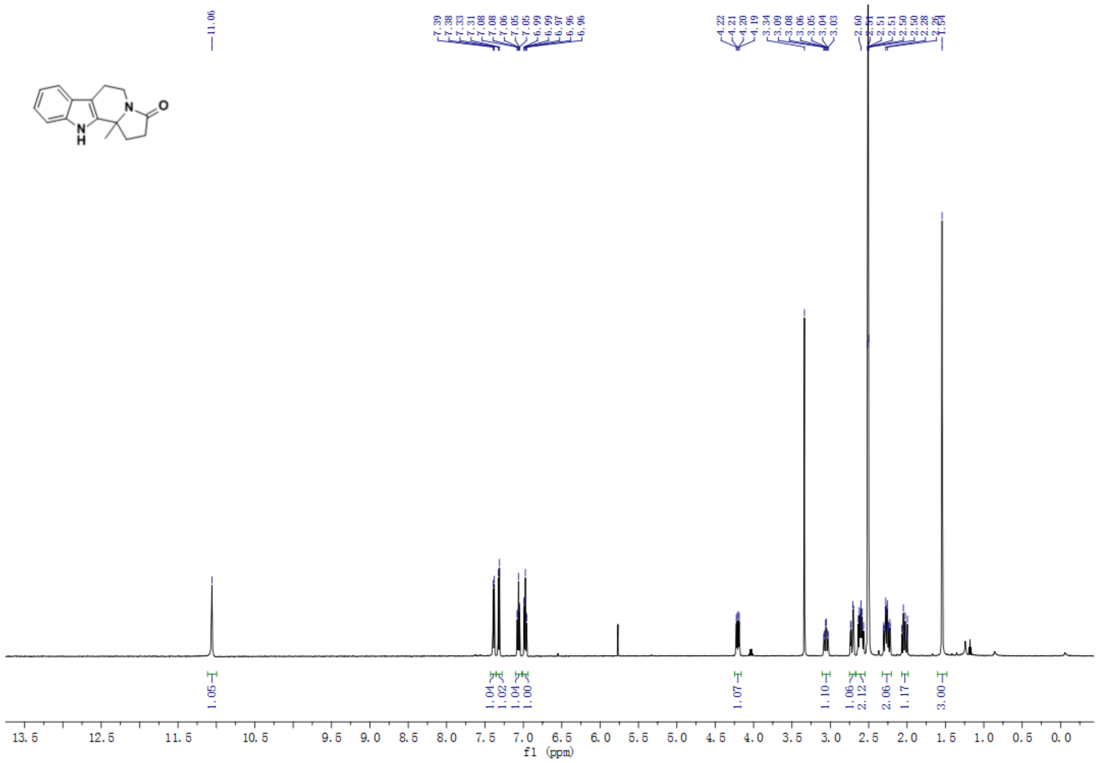

**Figure S11** $^{1}$H NMR spectrum of **SF1a** in dimethyl sulfoxide-$d_6$.

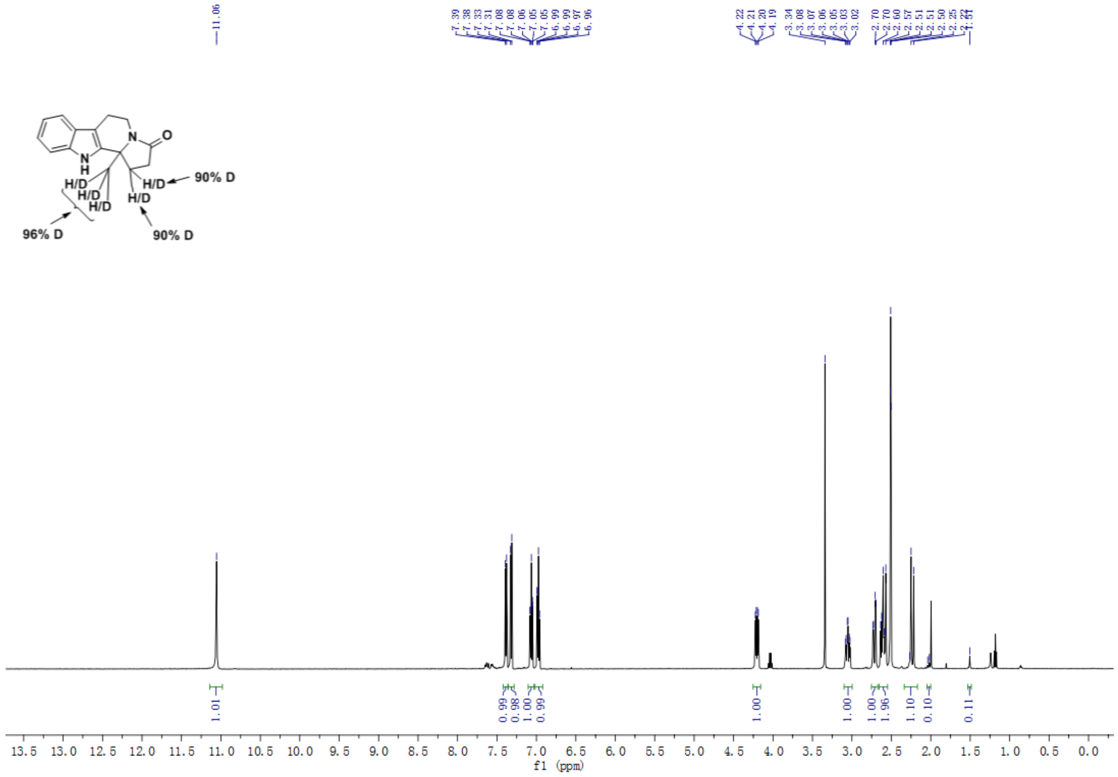

**Figure S12** $^{1}$H NMR spectrum of $[D]_n$-**SF1a** in dimethyl sulfoxide-$d_6$.

## Antibacterial bioassay

**Bacterial strains, culture and growth conditions, and sample preparation**

*Staphylococcus aureus (S. aureus)* was used in this study and cultured at 37 °C in Mueller-Hinton broth (MH broth). 5 mg compounds were dissolved in 100 μl DMSO, and the resulting solution was used as the sample stock. All the experiments were repeated at least three times.

**Preliminary screening of antibacterial activities**

The preliminary antibacterial activities against *S. aureus* strain were investigated in 96-well plates, and DMSO was used as the blank control. Briefly, *S. aureus* strain was seeded into 200 μl MH broth per well to make a density of $1 \times 10^5$ CFU/ml. Subsequently, an aliquot of the sample stock was added to make a final compound concentration of 100 μg/ml. After that, the optical density (OD) of the mixture in each well at 600 nm wavelength was immediately measured by a spectrometer, and recorded as $OD_0$. Then the plate was incubated at 37 °C for 24 h, after that, the OD of the mixture in each well was immediately measured again and recorded as $OD_{24}$. ΔOD ($\Delta OD = OD_{24} - OD_0$) was calculated and used to evaluate the antibacterial potency of the compounds. Finally, compounds with ΔOD lower than 0.1 were selected out for further study.

**Minimal inhibitory concentration (MIC) study**

The determination of minimal inhibitory concentration (MIC) of tested compounds was carried out in 96-well plates with DMSO as the blank control. Briefly, *S. aureus* strain was seeded into 200 μl MH broth per well to make a density of $1 \times 10^5$ CFU/ml. Subsequently, an aliquot of the sample stock was added to make 5 final compound concentrations (5 μg/ml, 10 μg/ml, 25 μg/ml, 50 μg/ml, 100 μg/ml and 200 μg/ml). After that, the OD of the mixture in each well at 600 nm wavelength was immediately measured by a spectrometer, and recorded as $OD_0$. Then the plates were incubated at 37 °C for 24 h, after that, the OD of the mixture in each well was immediately measured again and recorded as $OD_{24}$. ΔOD ($\Delta OD = OD_{24} - OD_0$) was calculated and used to determine the $MIC_{90}$. $MIC_{90}$ was determined as the lowest concentration that inhibited 90% bacteria growth as compared with DMSO control group.

**Time-Kill Assays**

Time-kill assays were performed in 96-well plates, and DMSO was used as the blank control. Briefly, *S. aureus* strain was seeded into 200 μl MH broth per well to make a density of $1 \times 10^5$ CFU/ml. Subsequently, an aliquot of the sample stock was added to make 5 final compound concentrations (5 μg/ml, 10 μg/ml, 25 μg/ml, 50 μg/ml, 100 μg/ml and 200 μg/ml). After that, the OD of the mixture in each well at 600 nm wavelength was instantly measured by a spectrometer, and recorded as $OD_0$. Then the plate was incubated at 37 °C for 2 h, 4 h, 6 h, 8 h, 10 h, 12 h and 24 h, after that, the OD of the mixture in each well was immediately measured again and recorded as $OD_t$. ΔOD ($\Delta OD = OD_t - OD_0$) was calculated and used to draw the time-kill curves.

**Colony-forming units (CFU) study**

At the end of the time-kill assays, the plates were used for CFU study. Concisely, parallel wells were randomly selected and diluted by $10^5$ times with MH broth. Then 100 μl diluent was taken and spread on Mueller-Hinton agar. The agar plates were incubated at 37 °C for 24 h. After that, the agar plates were recorded.

**Statistical analysis:** Statistical calculations were processed with Origin Pro 7.5 and Excel 2016.

## Antibacterial results and discussion

### Preliminary screening results

Preliminary screening disclosed that 21 compounds from the library showed antibacterial activities against the growth of *S. aureus* strain at the concentration of 100 μg/ml (Figure S13), and five of them (compounds **SF9d**, **SF29b**, **SF33**, **SF36** and **SF41**) showed good antibacterial activities, which were selected for further study.

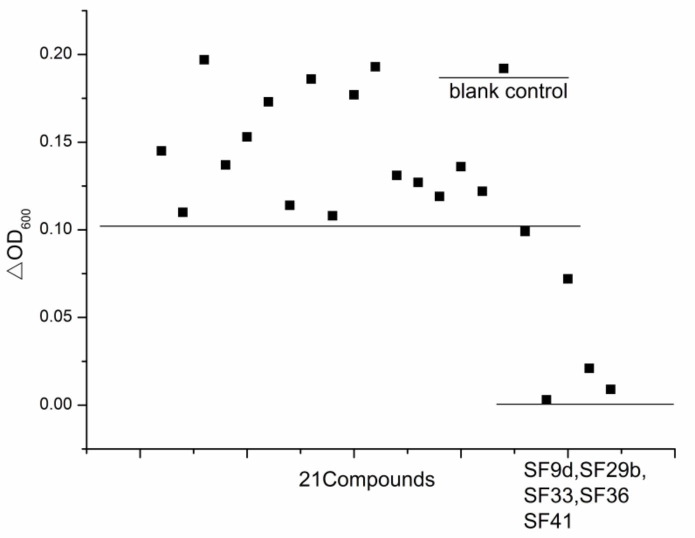


**Figure S13** Preliminary screening of antibacterial activities of compounds at 100 μg/ml.

### Time-kill assays and colony-forming unit (CFU) studies of compounds SF9d, SF29b, SF33, SF36 and SF41

As shown in Figure S14-S24, time-kill assays and colony-forming units (CFU) studies were also conducted with compounds **SF9d**, **SF29b**, **SF33, SF36** and **SF41**. Among them, compound **SF36** displayed the most potent antibacterial activity against *S. aureus* strain. Time-kill assay showed that **SF36** was bactericidal within 2-24 h at the concentration of 25 μg/ml, preventing bacterial growth of *S. aureus* strain completely (Figure 17). Colony-forming units (CFU) study of **SF36** was also carried out (Figure 23). The results showed that the number of clones on the agar plate decreased significantly in a dose-dependent manner, and only few clone was observed at the concentration of 50 μg/ml, indicating the antibacterial potency of this compound intuitively.

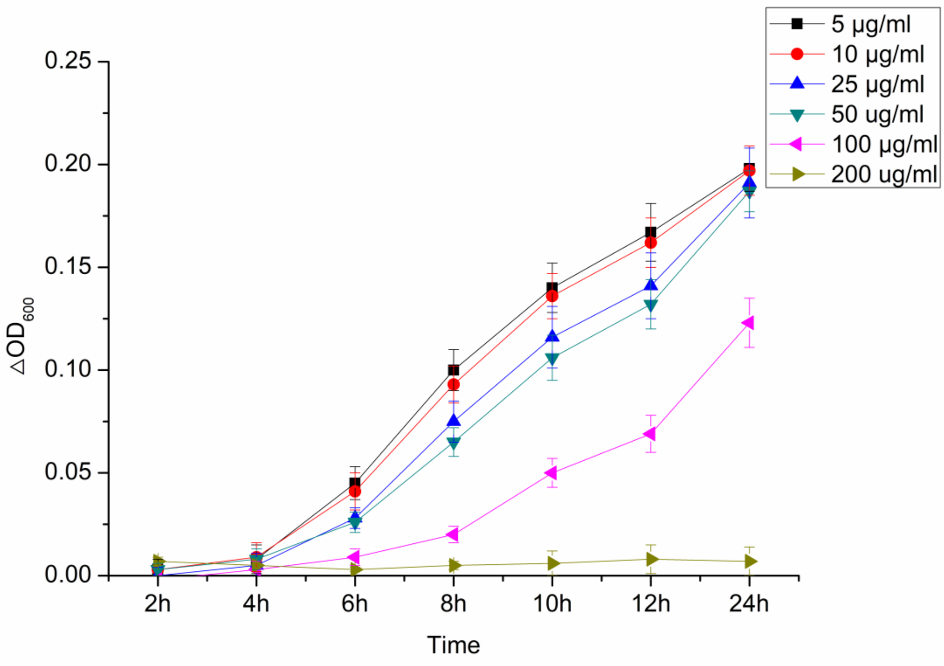


**Figure S14** Time-kill results of compound **SF9d** against *S. aureus* strain.

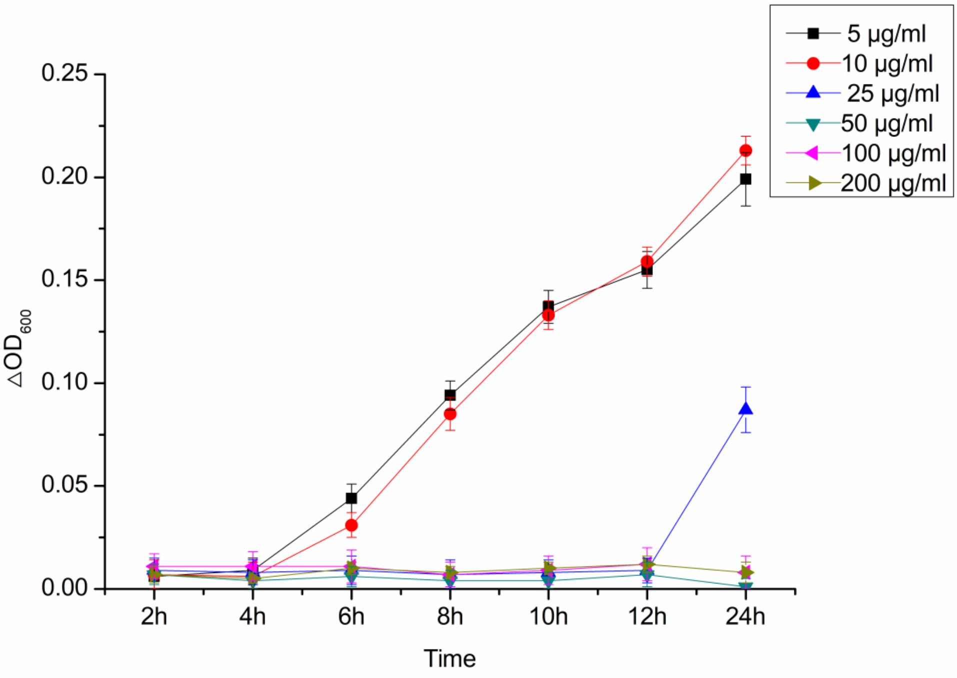


**Figure S15** Time-kill results of compound **SF29b** against *S. aureus* strain.

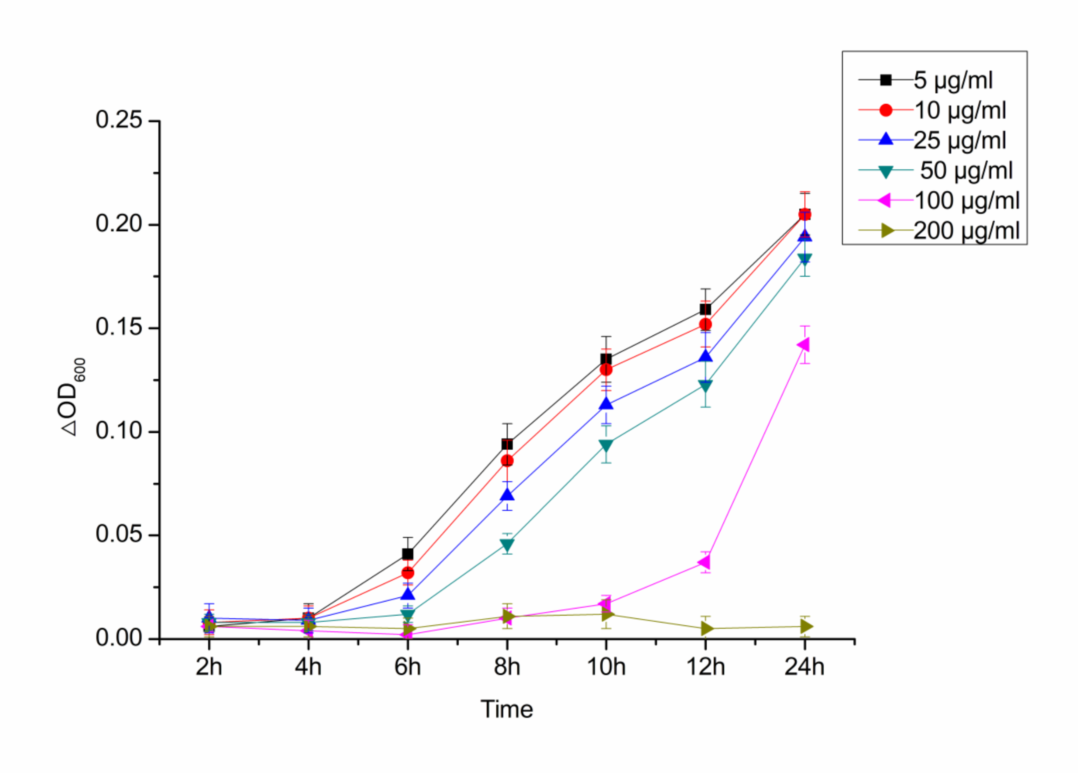


**Figure S16** Time-kill results of compound **SF33** again*st S. aureus* strain.

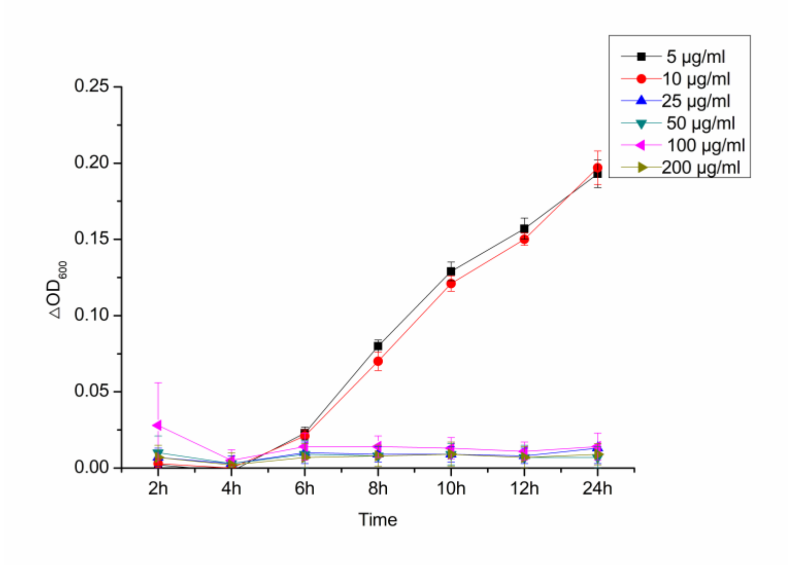


**Figure S17** Time-kill results of compound **SF36** against *S. aureus* strain.

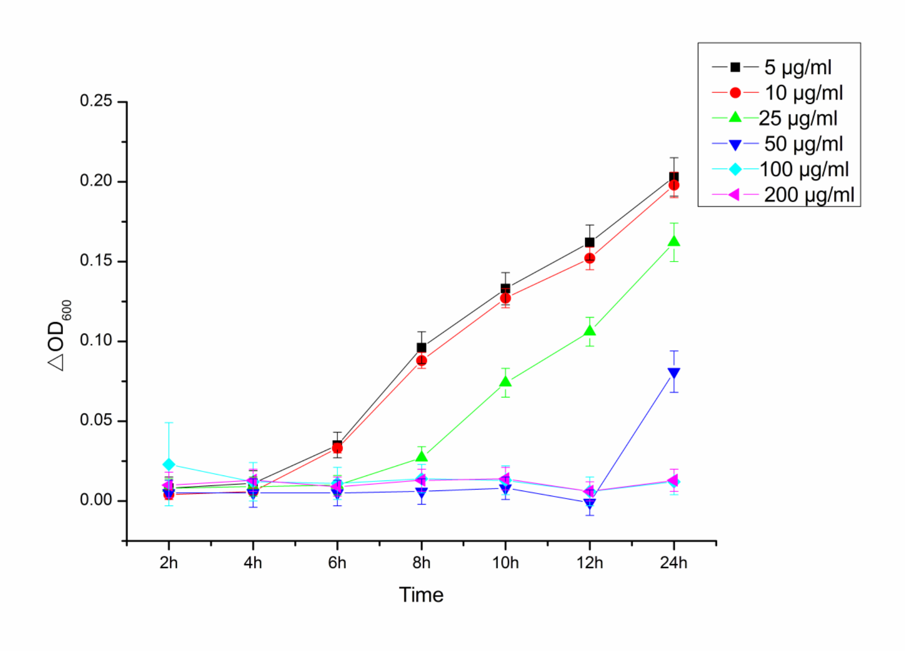


**Figure S18** Time-kill results of compound **SF41** against *S. aureus* strain.

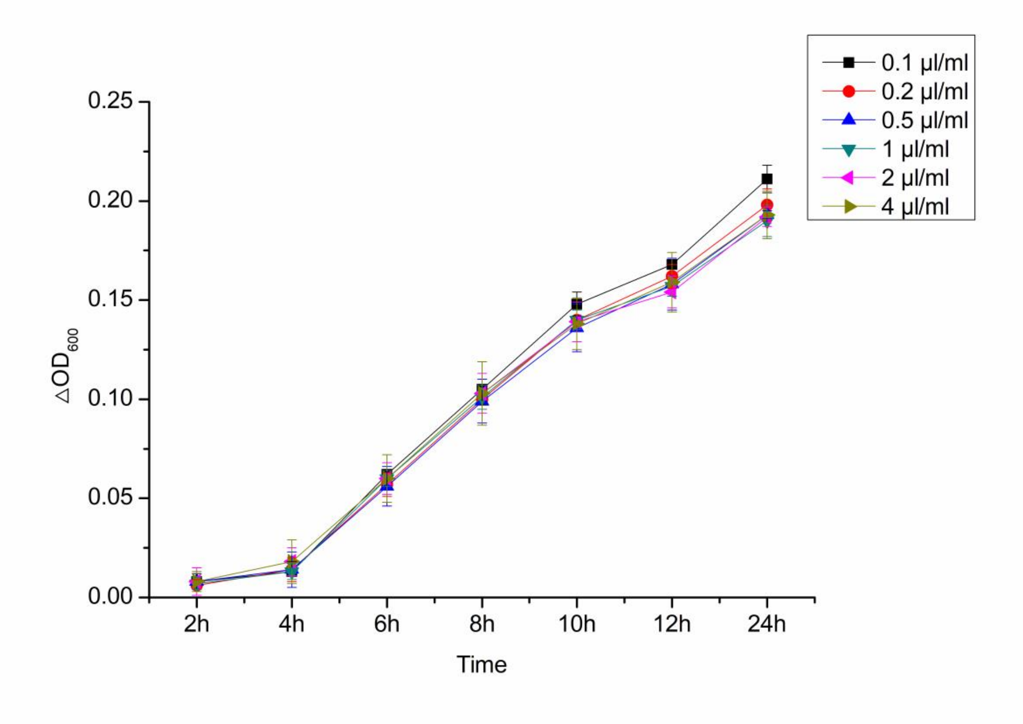


**Figure S19** Time-kill results of DMSO against *S. aureus* strain.

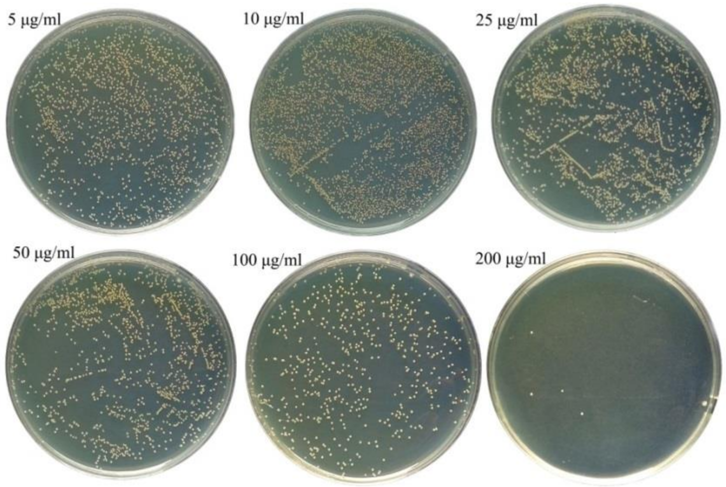


**Figure S20** CFU results of compound **SF9d**.

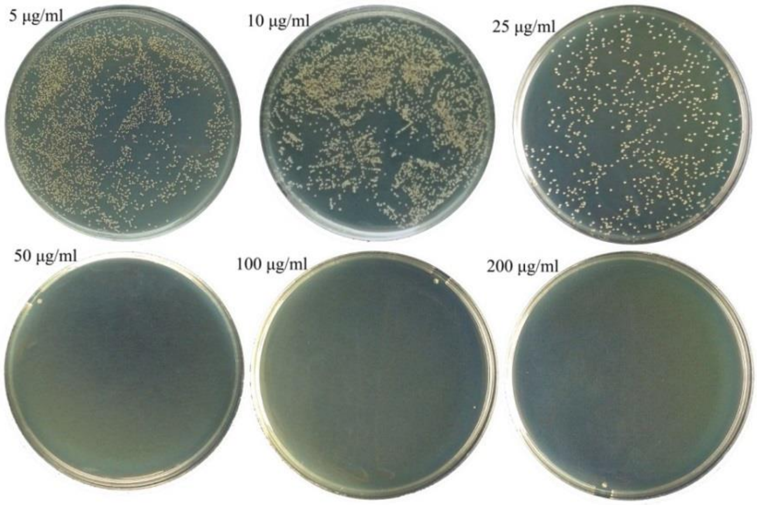


**Figure S21** CFU results of compound **SF29b**.

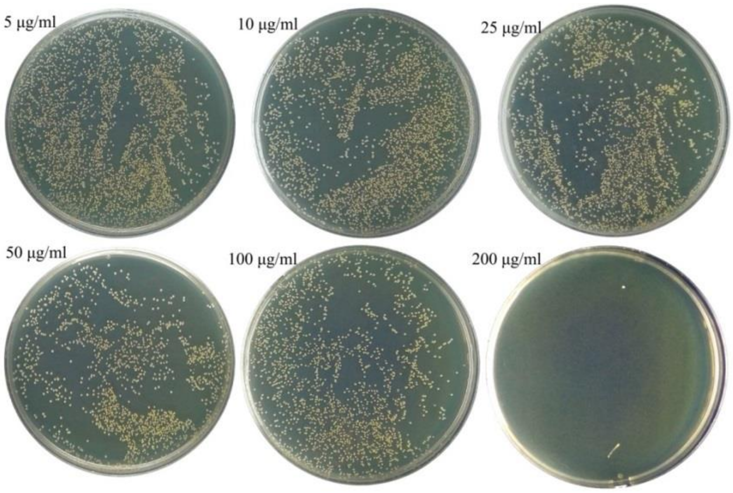


**Figure S22** CFU results of compound **SF33**.

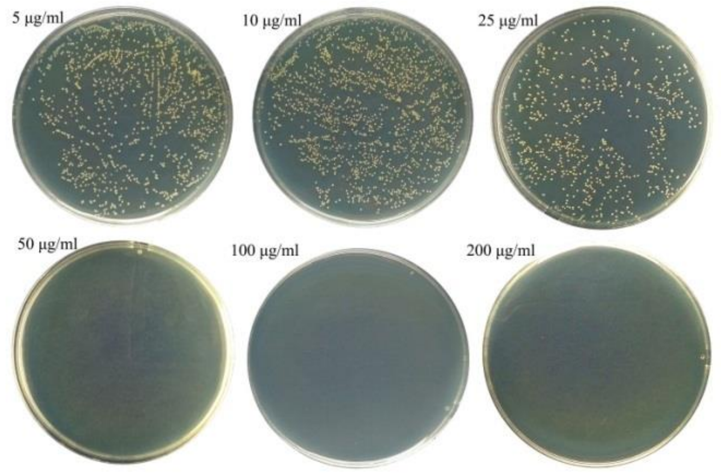


**Figure S23** CFU results of compound **SF36**.

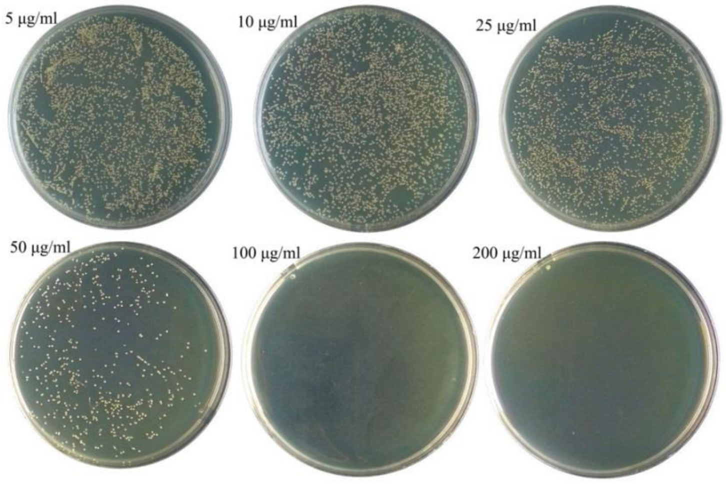


**Figure S24** CFU results of compound **SF41**.

## Copies of $^{1}H$ and $^{13}C$ NMR spectra of new compounds

**9-fluoro-12b-methyl-1,5,6,12b-tetrahydropyrrolo[2',1':3,4]pyrazino[1,2-*a*]indol-3(2*H*)-one (SF9d)**

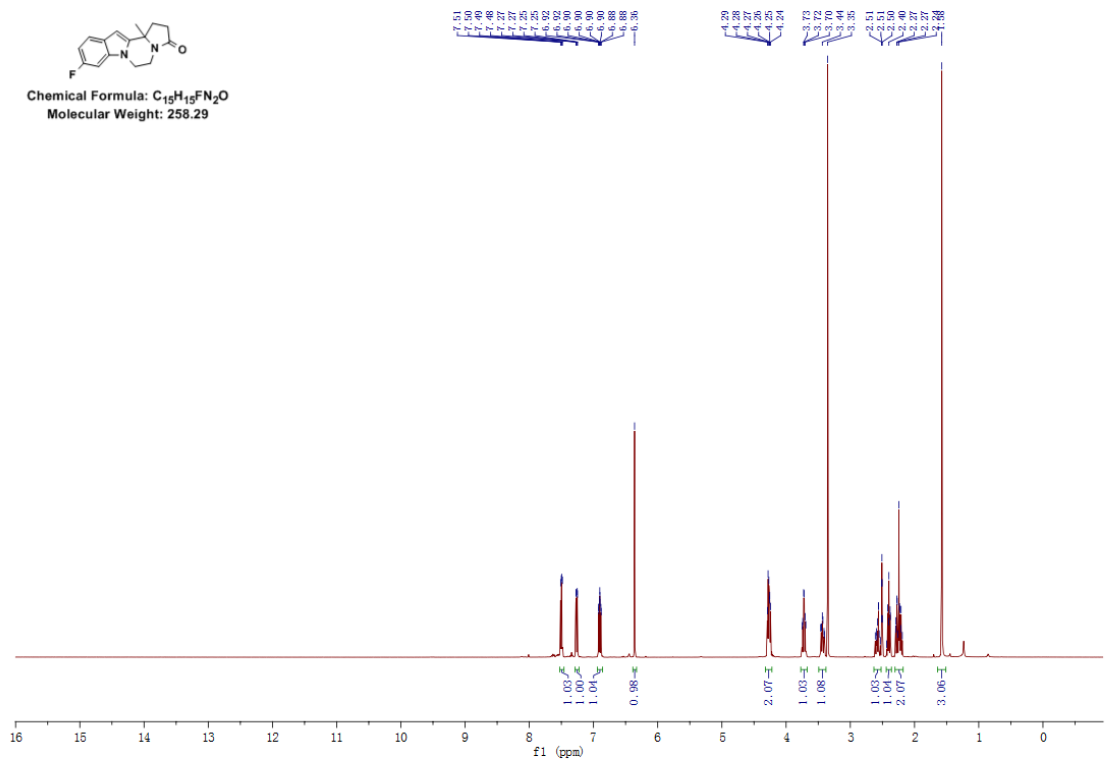


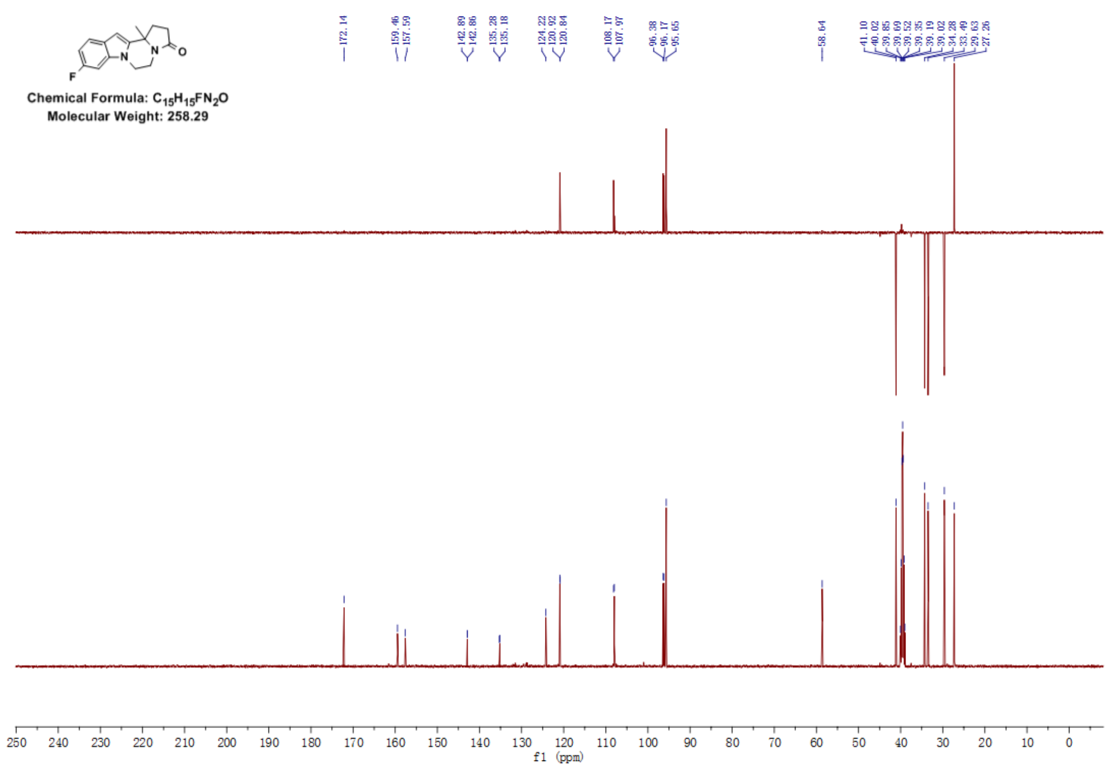

**2-hexyl-14b-methyl-1,14b-dihydroindolo[1,2-*a*]pyrrolo[2,1-*c*]quinoxalin-3(2*H*)-one (SF14b)**

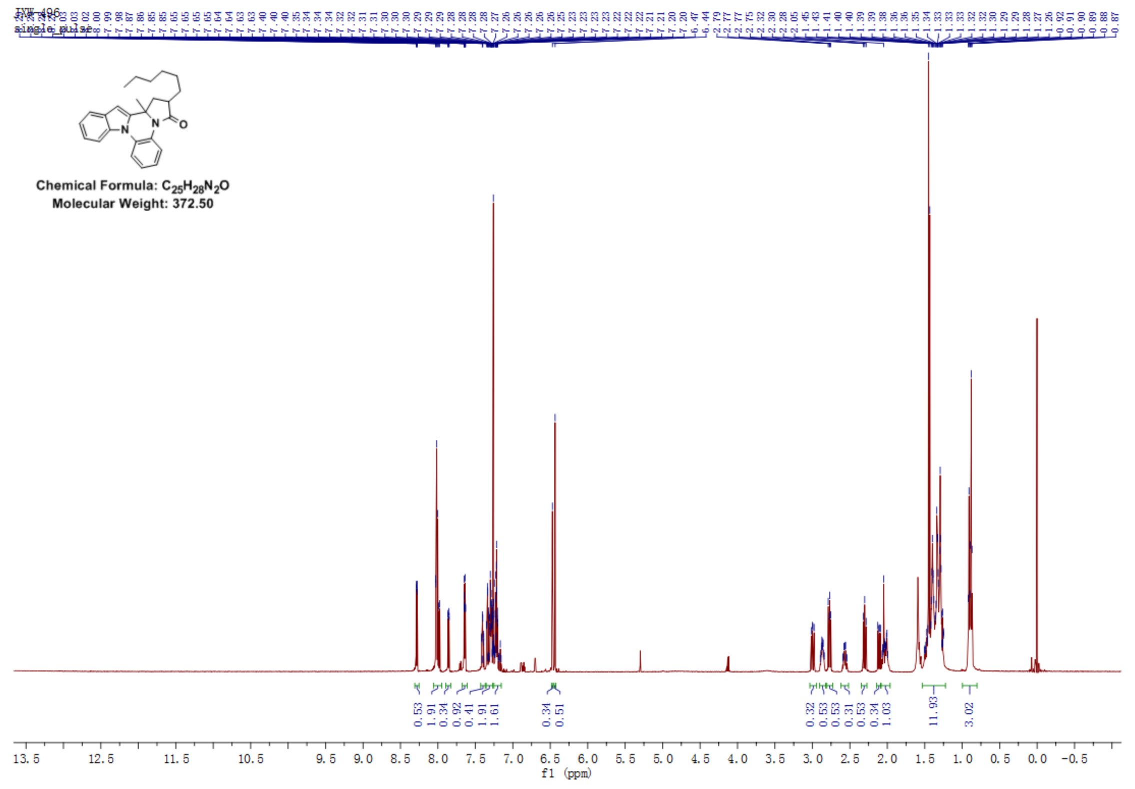


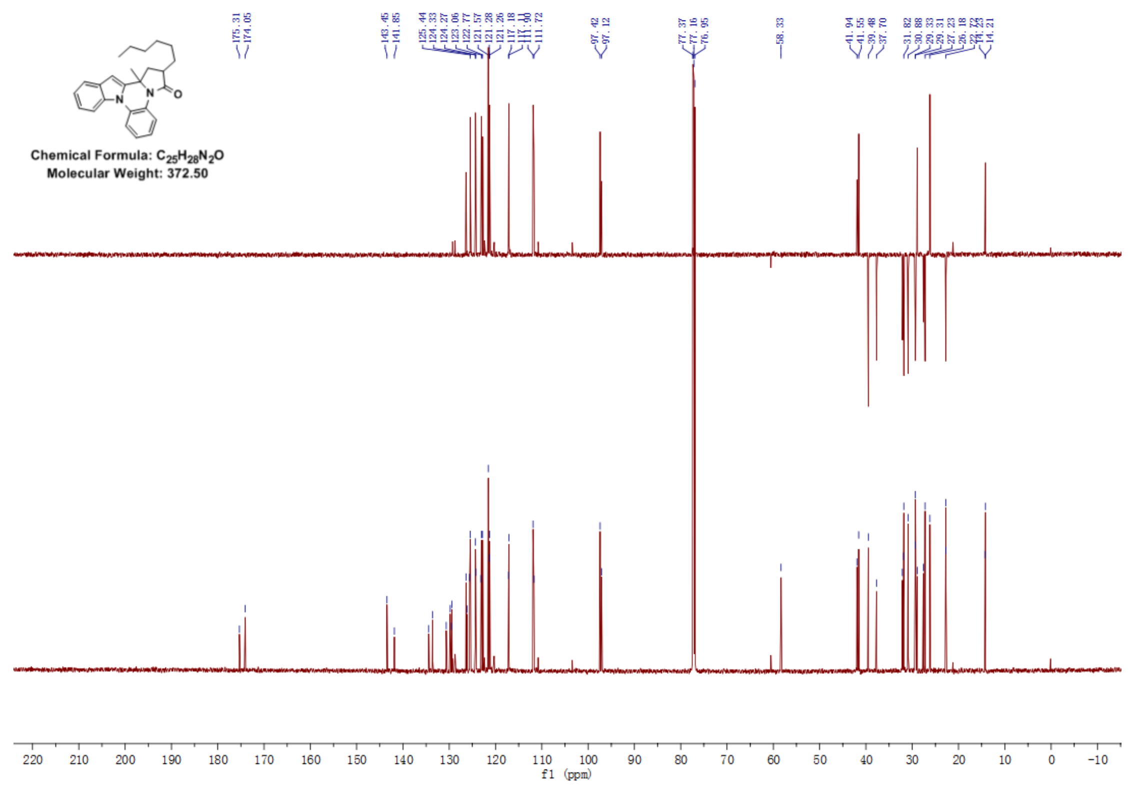

**2-hexyl-8,9-dimethoxy-10b-methyl-1,5,6,10b-tetrahydropyrrolo[2,1-*a*]isoquinolin-3(2*H*)-one (SF24b)**

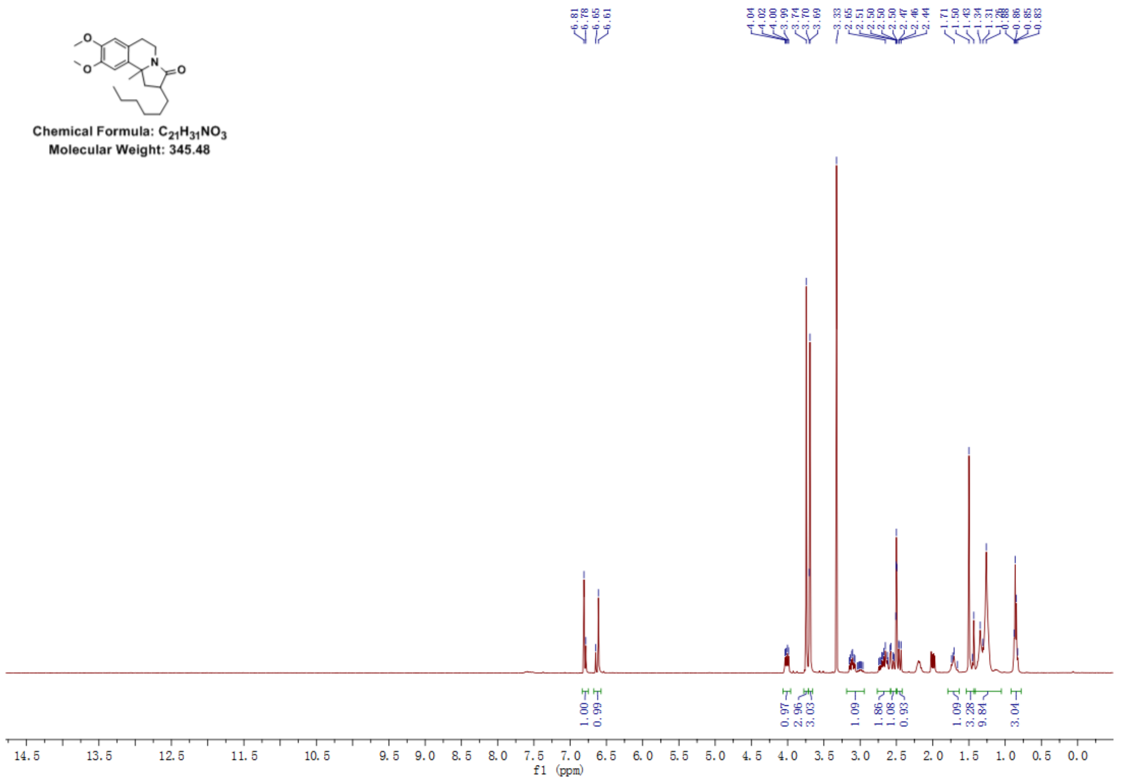


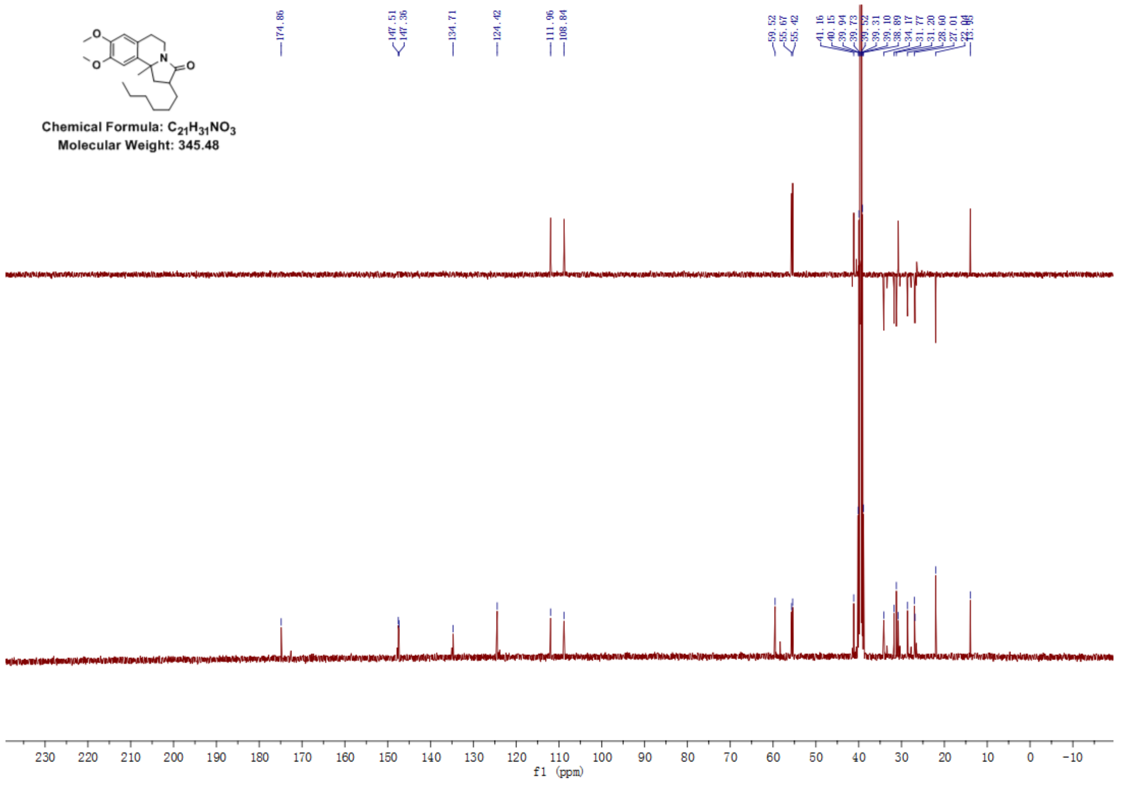

**2-hexyl-3a-methyl-3,3a-dihydro-1*H*-benzo[*d*]pyrrolo[2,1-*b*][1,3]oxazine-1,5(2*H*)-dione (SF35b)**

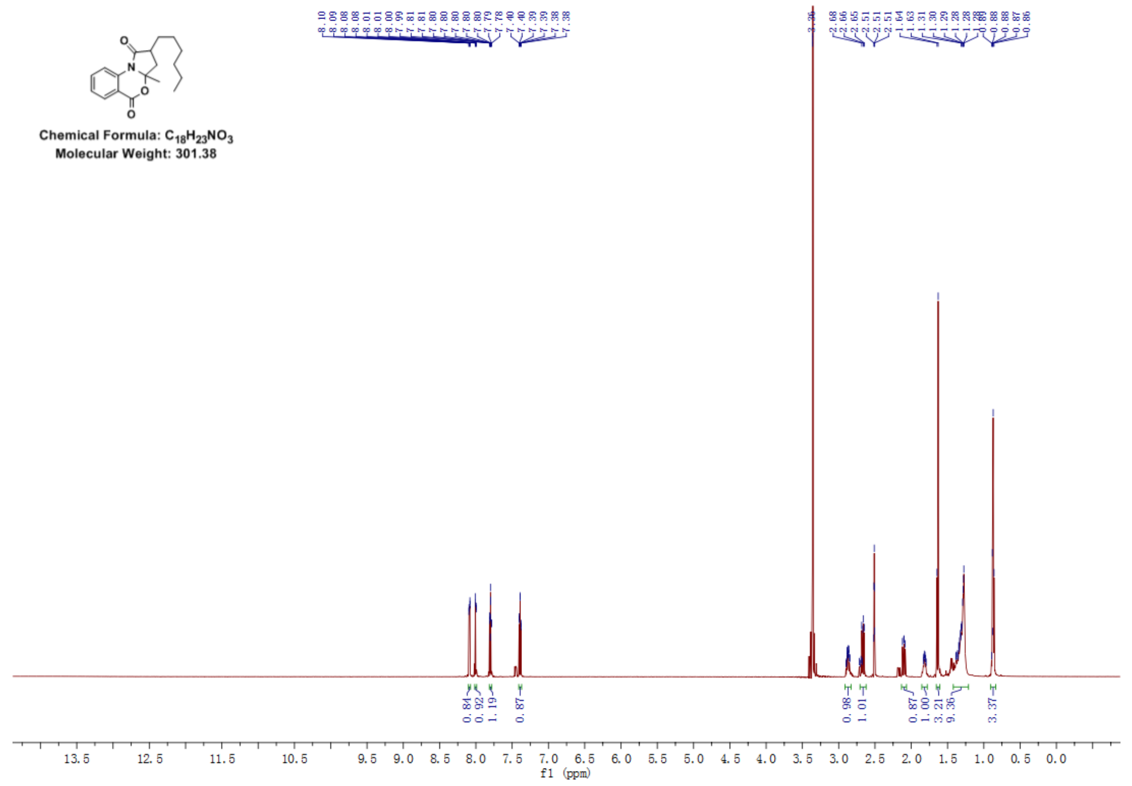


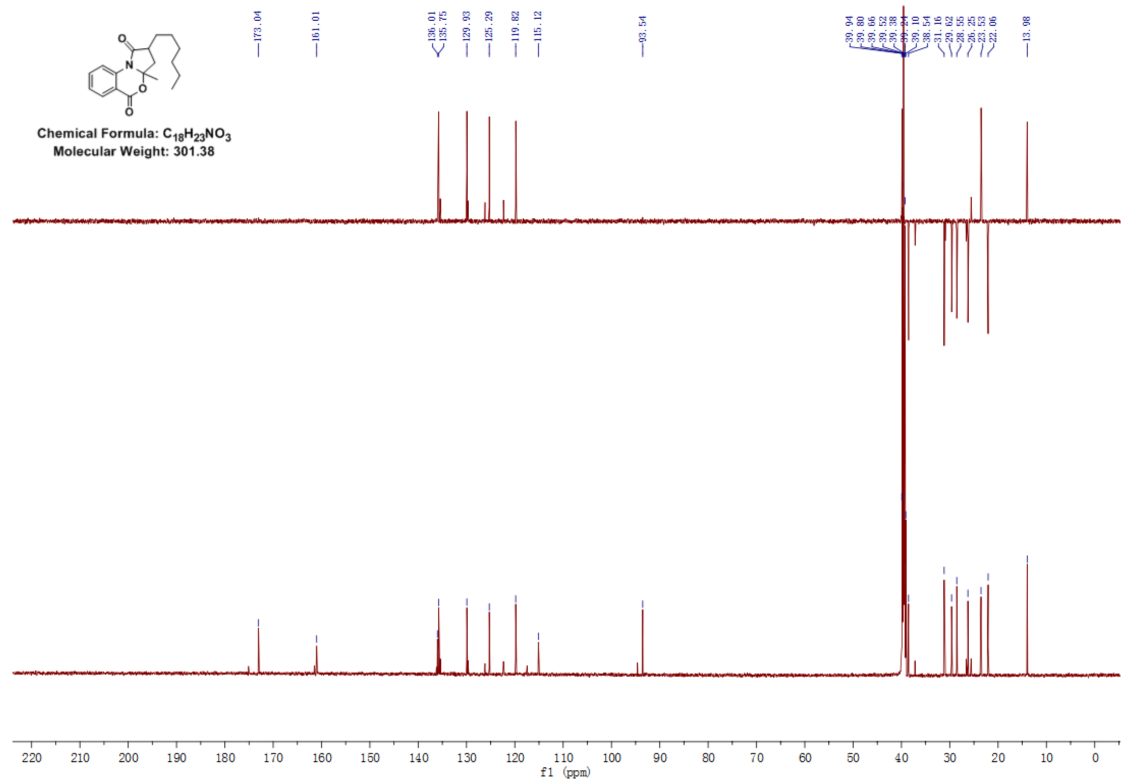

**11b-methyl-2,3,5,6,11,11b-hexahydro-1*H*-indolizino[8,7-*b*]indole (SF47)**

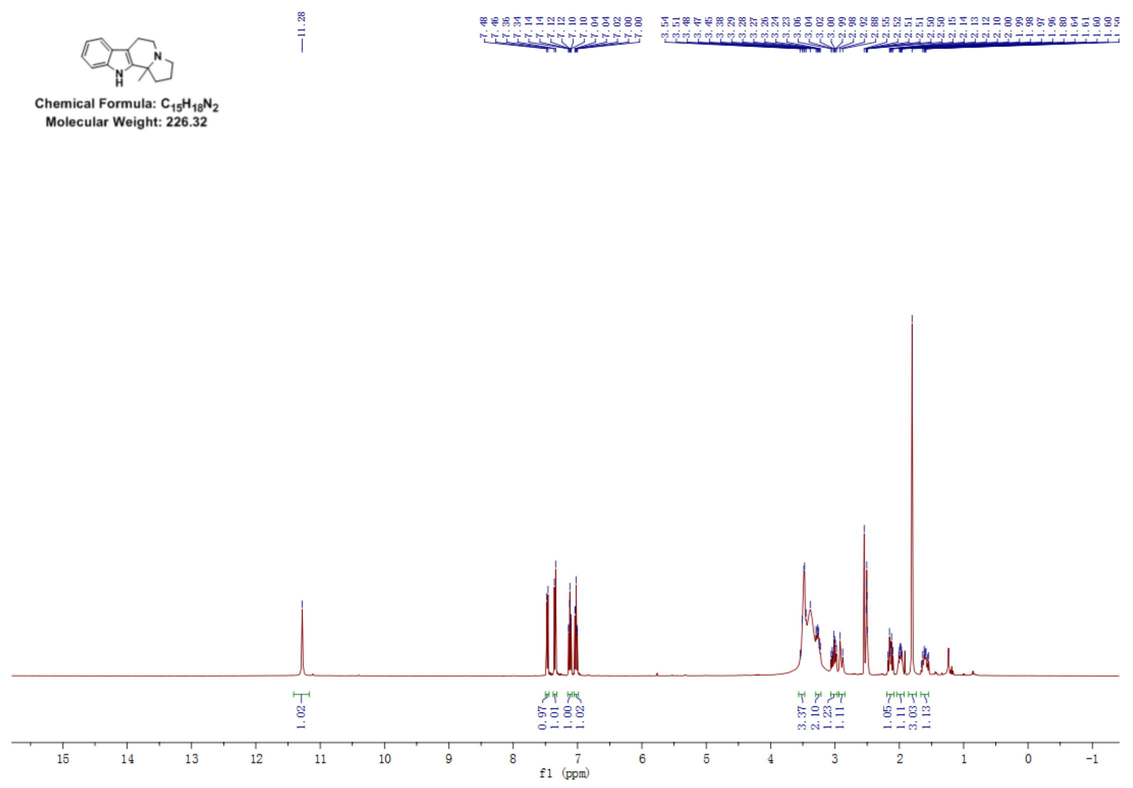


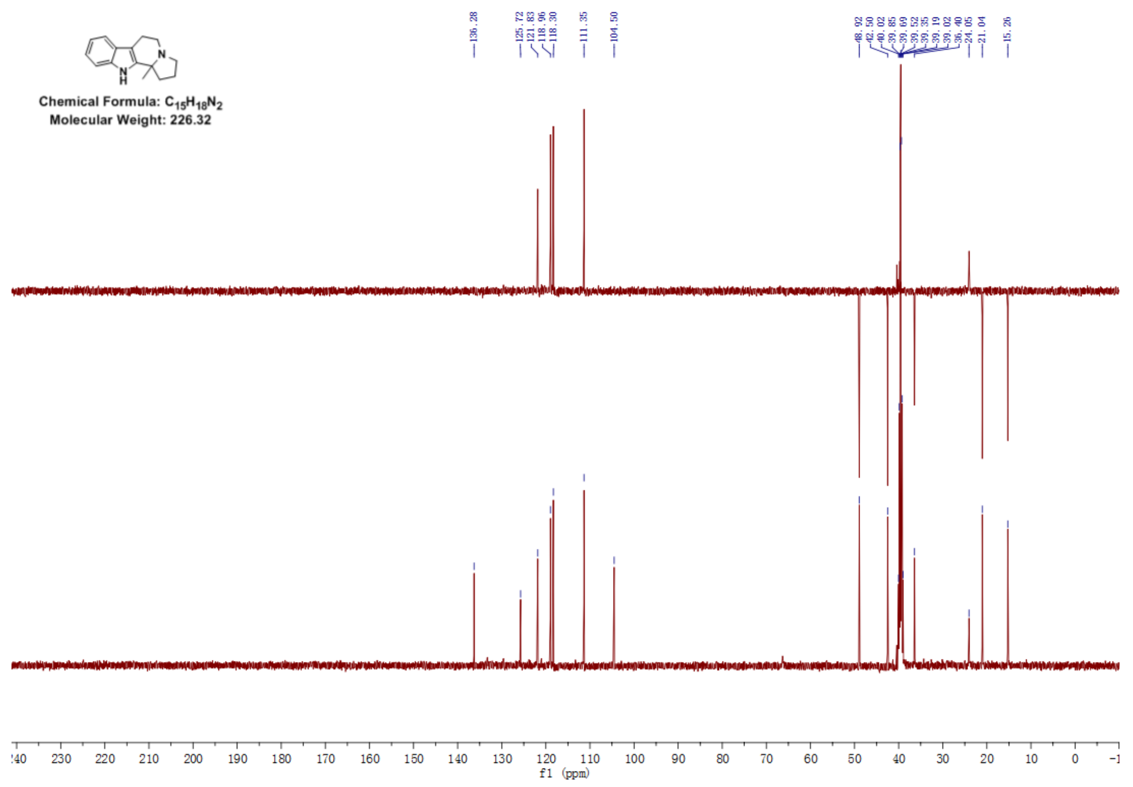

## 11c-methyl-2,3,5,6,7,11c-hexahydro-1*H*-indolizino[7,8-*b*]indole (SF50)

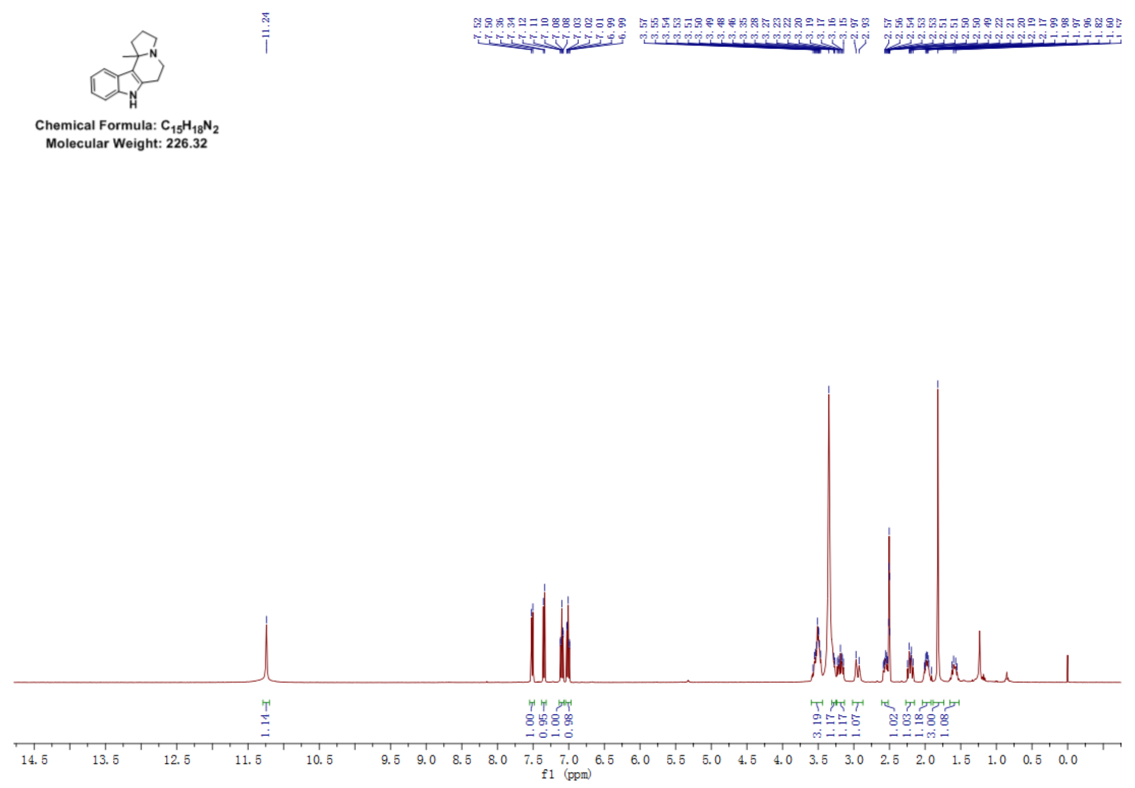

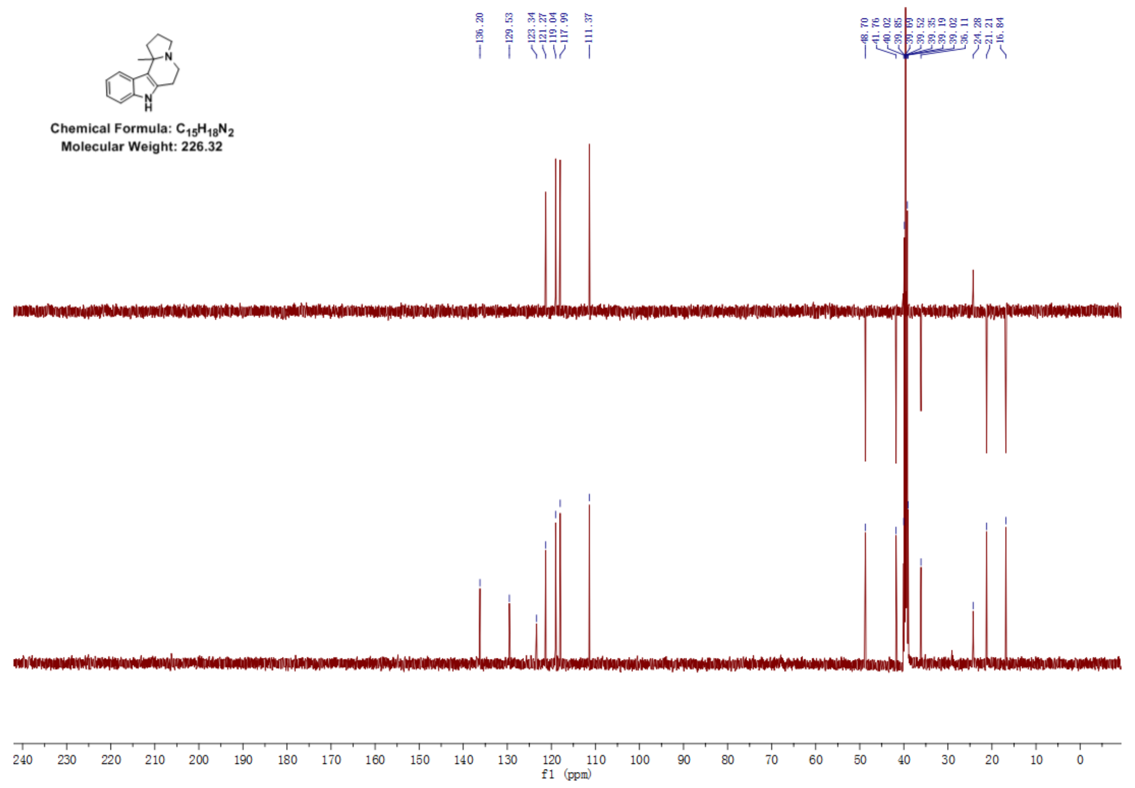

**14c-methyl-5,6,8,9,10,14c-hexahydroindolo[3',2':3,4]pyrido[2,1-*a*]isoquinoline (SF51)**

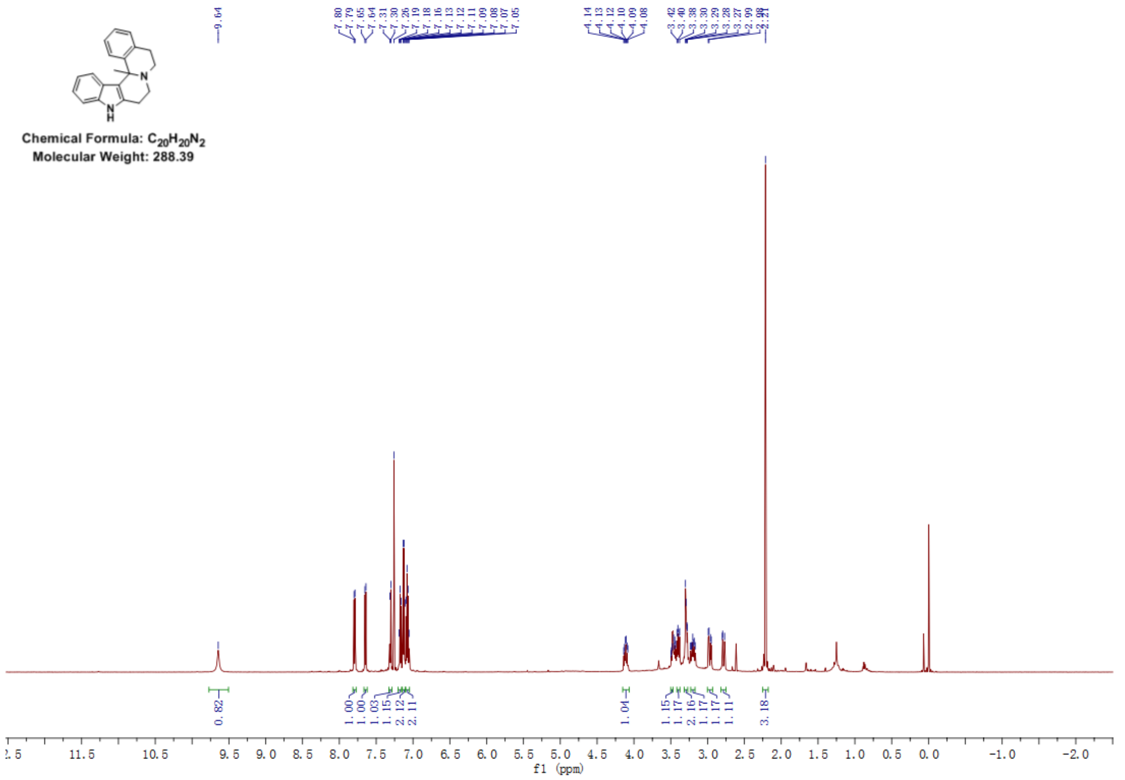


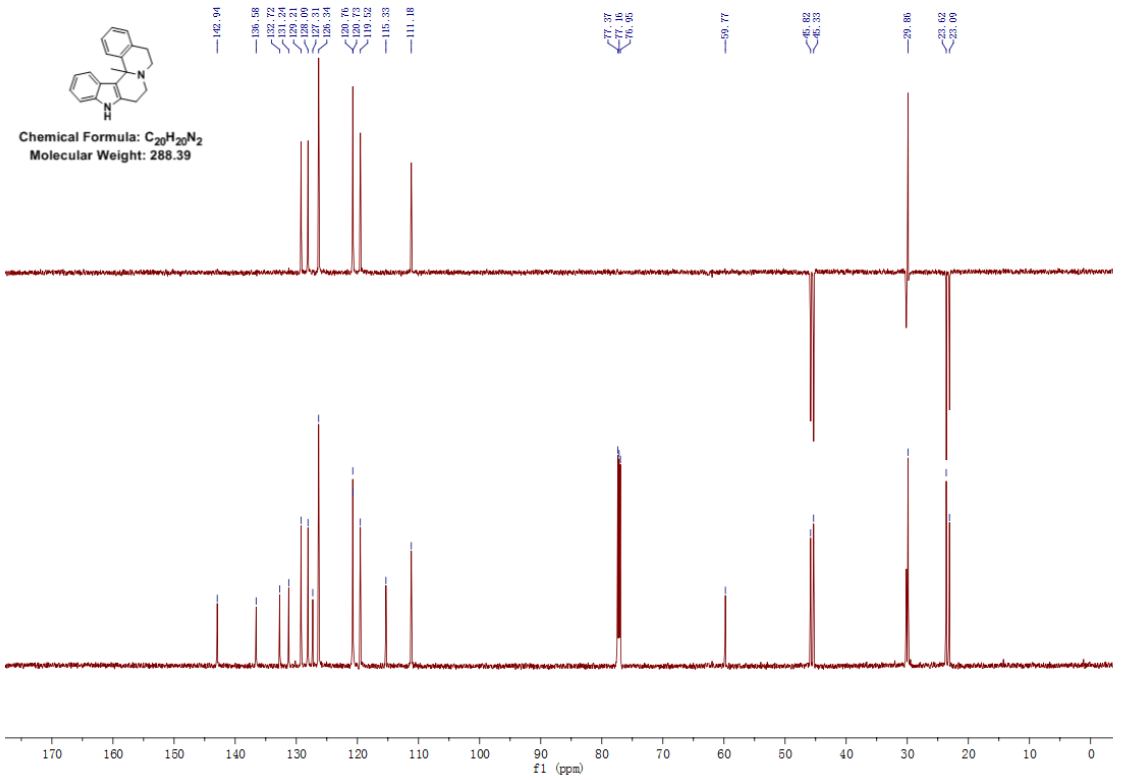

**15b-methyl-6,8,9,15b-tetrahydro-5*H*-indolo[2',1':3,4]pyrazino[2,1-*a*]isoquinoline (SF52)**

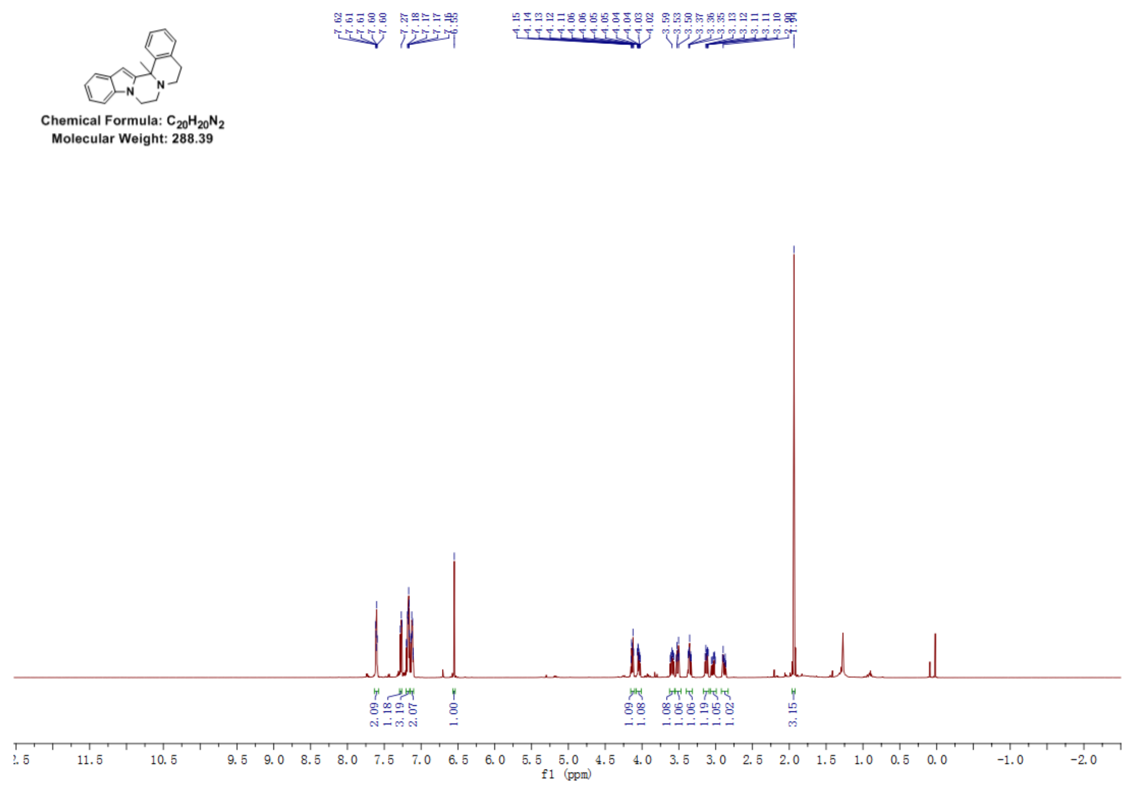


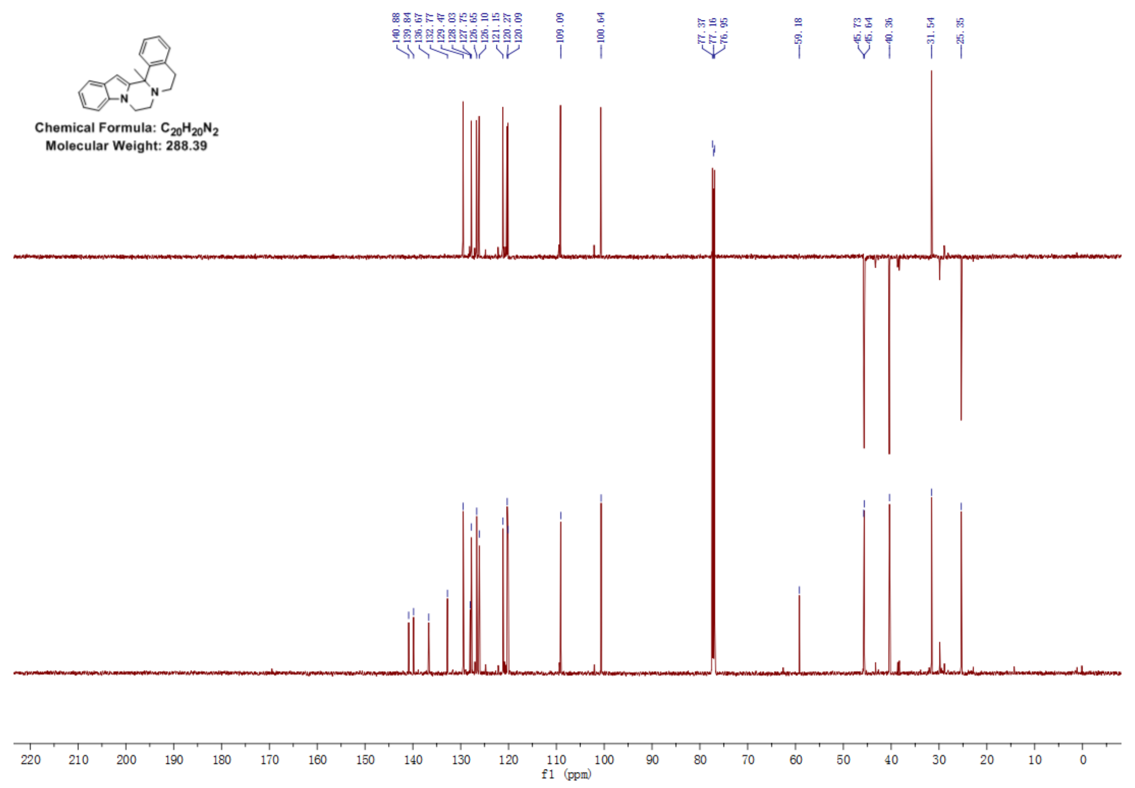

**11b-methyl-4,5,7,11b-tetrahydro-3*H*-pyrrolo[3',2':3,4]pyrido[2,1-*a*]isoindole (SF53)**

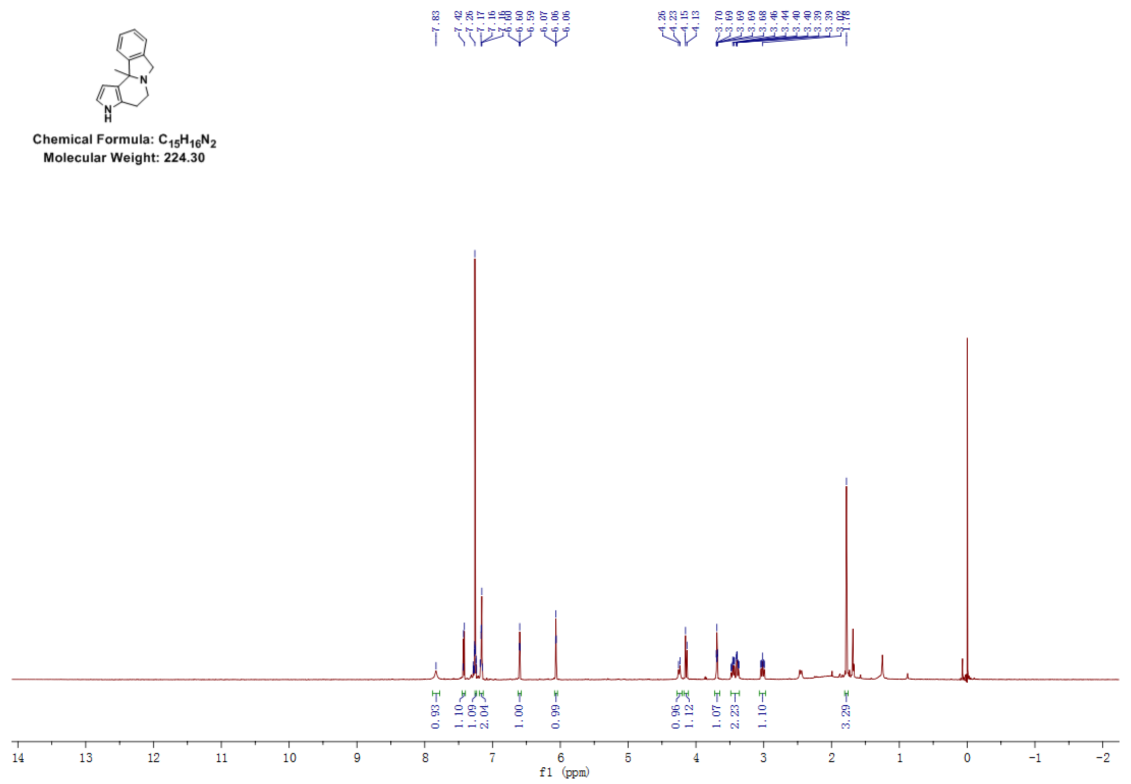


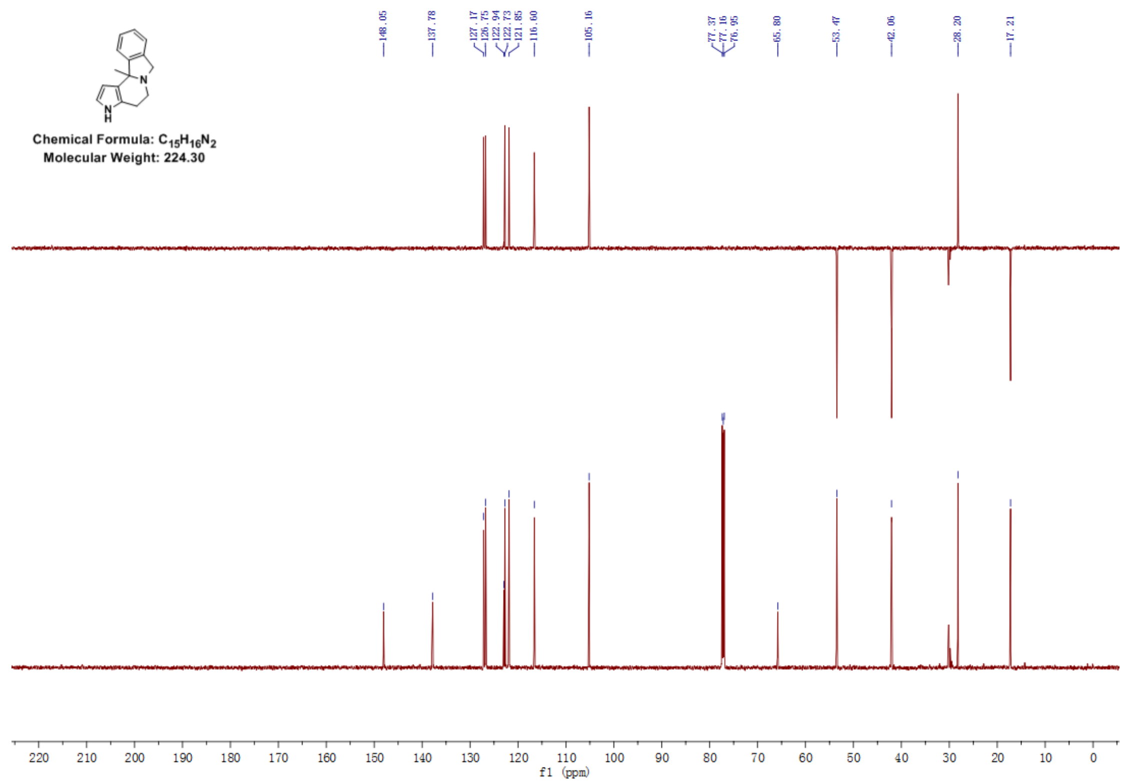

**11b-methyl-4,5,7,11b-tetrahydrothieno[3',2':3,4]pyrido[2,1-*a*]isoindole (SF54)**

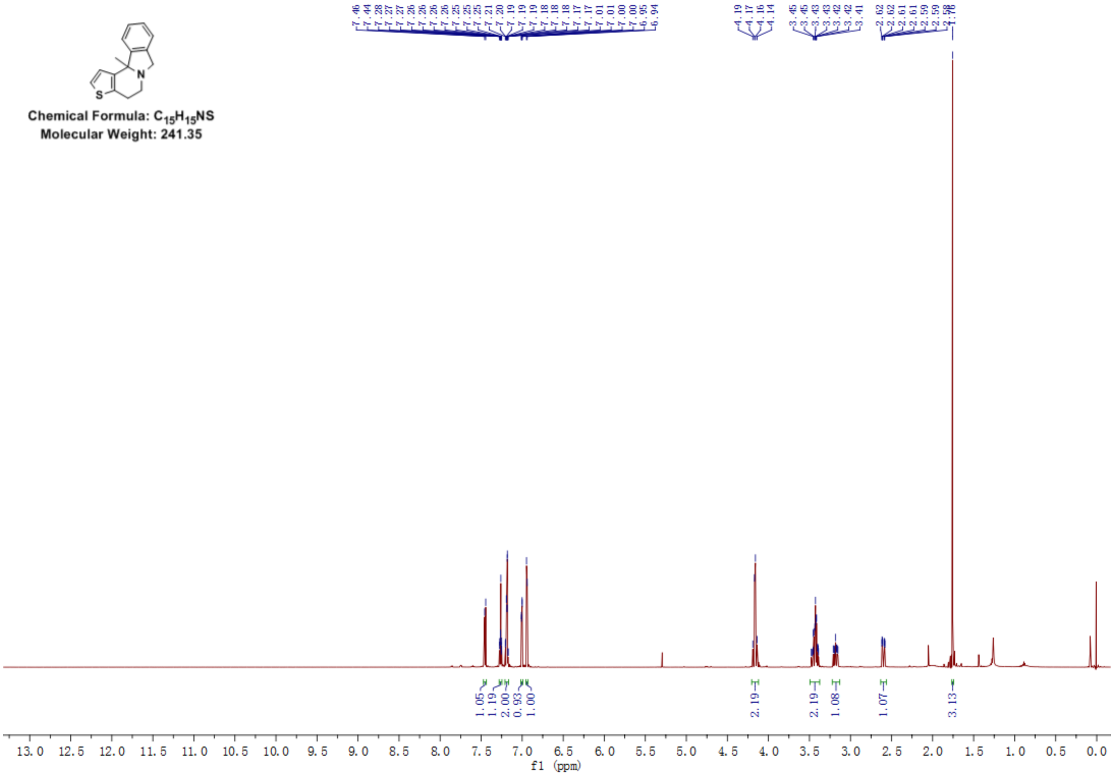


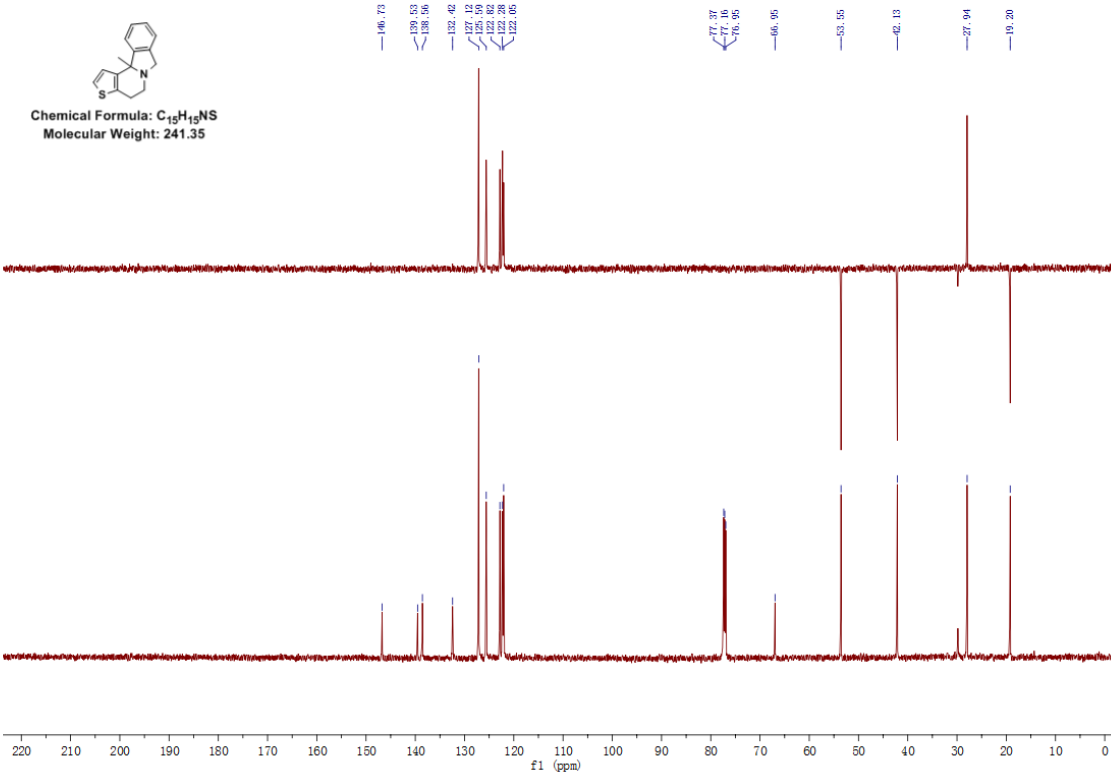

**11b-methyl-4,5,7,11b-tetrahydrothieno[2',3':3,4]pyrido[2,1-*a*]isoindole (SF55)**

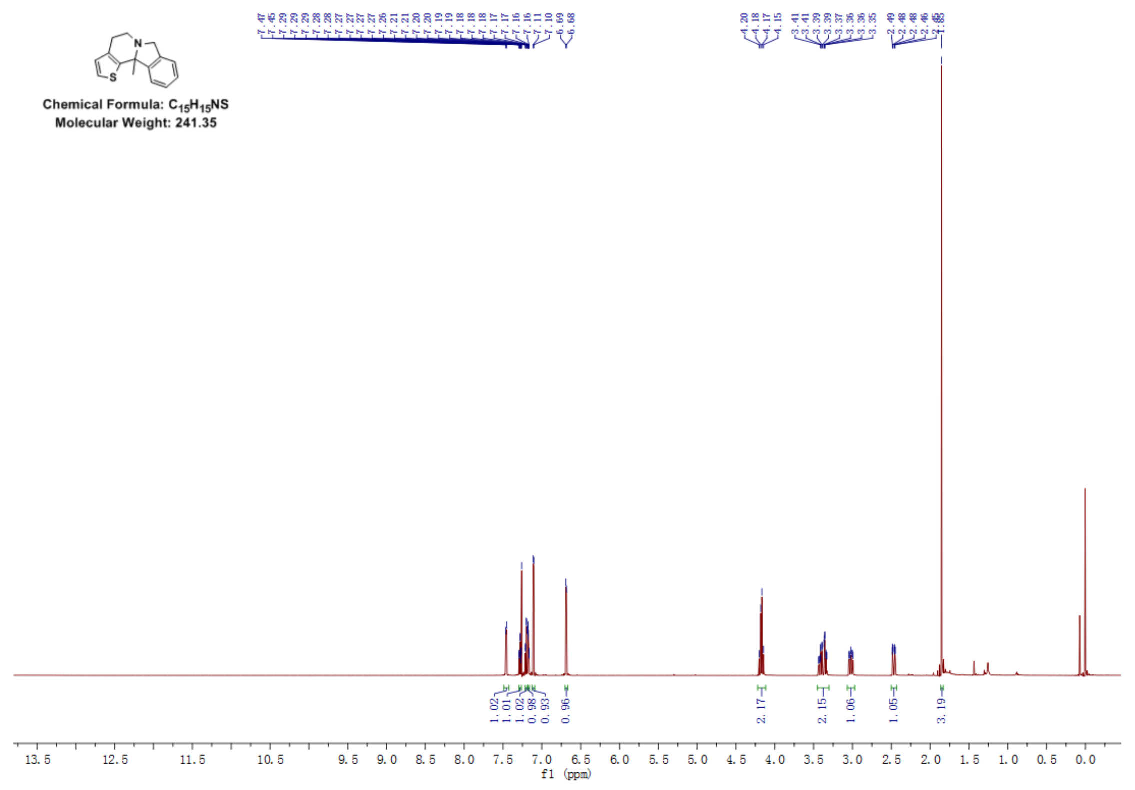


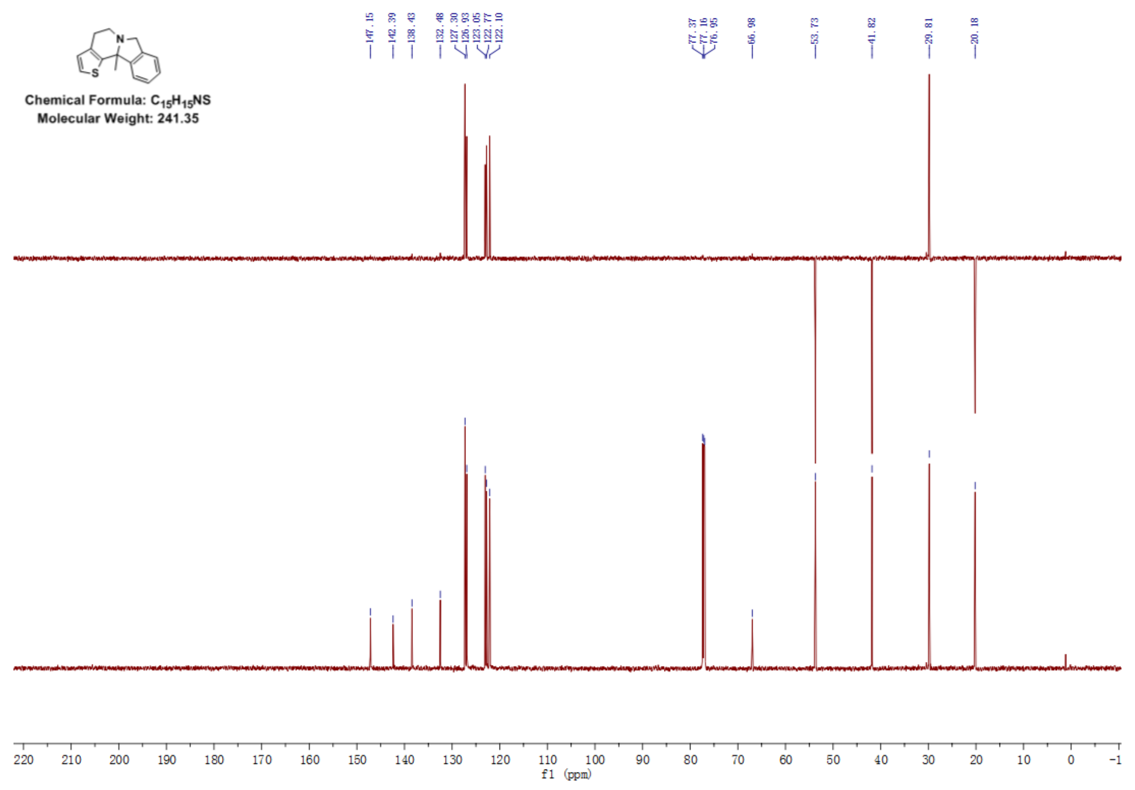

Supplement: Supplementary file 1 [file molecules-24-00988-s001.pdf]
